# The Src–ZNRF1 axis controls TLR3 trafficking and interferon responses to limit lung barrier damage

**DOI:** 10.1084/jem.20220727

**Published:** 2023-05-09

**Authors:** You-Sheng Lin, Yung-Chi Chang, Tai-Ling Chao, Ya-Min Tsai, Shu-Jhen Jhuang, Yu-Hsin Ho, Ting-Yu Lai, Yi-Ling Liu, Chiung-Ya Chen, Ching-Yen Tsai, Yi-Ping Hsueh, Sui-Yuan Chang, Tsung-Hsien Chuang, Chih-Yuan Lee, Li-Chung Hsu

**Affiliations:** 1 Institute of Molecular Medicine, College of Medicine, National Taiwan University, Taipei, Taiwan; 2 Department of Clinical Laboratory Sciences and Medical Biotechnology, College of Medicine, National Taiwan University, Taipei, Taiwan; 3 Genomics Research Center, Academia Sinica, Taipei, Taiwan; 4 Department of Pathology and Laboratory Medicine, Kaohsiung Veterans General Hospital, Kaohsiung, Taiwan; 5 Immunology Research Center, National Health Research Institutes, Zhunan, Taiwan; 6 Institute of Molecular Biology, Academia Sinica, Taipei, Taiwan; 7 Department of Laboratory Medicine, National Taiwan University Hospital, Taipei, Taiwan; 8 Department of Surgery, National Taiwan University Hospital, Taipei City, Taiwan; 9 Graduate Institute of Immunology, College of Medicine, National Taiwan University, Taipei, Taiwan; 10 Center of Precision Medicine, College of Medicine, National Taiwan University, Taipei, Taiwan

## Abstract

Type I interferons are important antiviral cytokines, but prolonged interferon production is detrimental to the host. The TLR3-driven immune response is crucial for mammalian antiviral immunity, and its intracellular localization determines induction of type I interferons; however, the mechanism terminating TLR3 signaling remains obscure. Here, we show that the E3 ubiquitin ligase ZNRF1 controls TLR3 sorting into multivesicular bodies/lysosomes to terminate signaling and type I interferon production. Mechanistically, c-Src kinase activated by TLR3 engagement phosphorylates ZNRF1 at tyrosine 103, which mediates K63-linked ubiquitination of TLR3 at lysine 813 and promotes TLR3 lysosomal trafficking and degradation. ZNRF1-deficient mice and cells are resistant to infection by encephalomyocarditis virus and SARS-CoV-2 because of enhanced type I interferon production. However, *Znrf1*^−/−^ mice have exacerbated lung barrier damage triggered by antiviral immunity, leading to enhanced susceptibility to respiratory bacterial superinfections. Our study highlights the c-Src–ZNRF1 axis as a negative feedback mechanism controlling TLR3 trafficking and the termination of TLR3 signaling.

## Teaser

E3 ubiquitin ligase ZNRF1 is activated by c-Src kinase following TLR3 activation and, in turn, promotes TLR3 ubiquitination and trafficking to lysosomes for degradation. This leads to properly regulated interferon production and alveolar barrier repair to prevent secondary bacterial superinfection.

## Introduction

Type I IFNs play a crucial role in antiviral immunity (McNab et al., 2015; Mesev et al., 2019). Upon viral infection, host cells sense viruses through various pattern-recognition receptors, including TLRs, by recognizing conserved viral molecules, mostly nucleic acids, and subsequently produce type I IFNs (Mesev et al., 2019). Type I IFNs not only induce transcription of many IFN-stimulated genes in infected and neighboring cells to suppress viral propagation but also are involved in the activation of adaptive immunity by promoting B and T cell responses (McNab et al., 2015). Despite its many beneficial effects in antiviral immunity, dysregulation of the type I IFN response contributes to the development and progression of inflammatory and autoimmune diseases (McNab et al., 2015). In addition, accumulated evidence demonstrates the detrimental effects of type I IFNs on the host in the late phase of infection by respiratory viruses, such as severe acute respiratory syndrome coronavirus 2 (SARS-CoV-2) and influenza viruses, by enhancing inflammation and tissue damage (McNab et al., 2015). Recent studies further revealed that type I IFNs impair lung tissue repair by suppressing alveolar epithelial cell proliferation through p53 signaling, thereby increasing susceptibility to opportunistic bacterial infections, termed bacterial superinfections (Broggi et al., 2020; Major et al., 2020). Therefore, production of type I IFNs must be tightly controlled in a spatiotemporal manner.

TLR3, a member of the endosomal TLR family, is expressed abundantly in immune and many non-immune cells and detects double-stranded RNA (dsRNA) derived from viruses or generated during viral replication (Brubaker et al., 2015). Results from human genetic and animal studies imply that TLR3-mediated signaling and type I IFNs are required for the control of influenza viruses, SARS-CoV-2, HSV-1, and encephalomyocarditis virus (EMCV; Casanova and Abel, 2021; Hardarson et al., 2007; Perales-Linares and Navas-Martin, 2013). TLR3 is the only TLR that signals exclusively through the adaptor protein, TIR domain-containing adapter-inducing IFN-β (TRIF); however, unlike TLR4, TLR3-activated TRIF signaling does not require the adaptor protein, TRIF-related adaptor molecule (Brubaker et al., 2015). Upon engaging with its ligand, TLR3 undergoes dimerization and conformational changes, then recruits TRIF and initiates a serial of signaling cascades that lead to activation of the transcriptional factors IFN regulatory factor 3 (IRF3) and NF-κB to promote type I IFN and proinflammatory cytokine production (Takeuchi and Akira, 2010).

Endosomal trafficking of TLRs is critical for their downstream signaling (Lee and Barton, 2014). Endosomal nucleic acid–sensing TLR3, TLR7, TLR8, and TLR9 must be delivered to the endocytic compartments, facilitated by the chaperone Unc-93 Homolog B1 (UNC93B1), and undergo proteolytic cleavage by cathepsins and asparagine endopeptidase to produce functional receptors, which are able to transmit signals to downstream molecules, upon ligand binding (Ewald et al., 2011; Rael and Barton, 2021; Tabeta et al., 2006). Recent studies have indicated that both TLR3 and TLR9, but not TLR7, have to be released from UNC93B1 in the endolysosomal compartment to enable binding to their ligands (Majer et al., 2019b). In addition, TLR3 has been reported to be ubiquitinated by TRIM3 to facilitate TLR3 trafficking to the endolysosomal compartment and initiate downstream signaling (Li et al., 2020). However, the process of termination of endosomal nucleic acid–sensing TLR signaling upon arrival in the endolysosomal compartment remains enigmatic. Interestingly, UNC93B1 was recently shown to undergo K63-linked polyubiquitination upon TLR7 activation and then to recruit Syntenin-1 to facilitate TLR7-UNC93B1 containing vesicle sorting into the intraluminal vesicles (ILVs) within the multivesicular bodies (MVBs) and terminate the signaling (Majer et al., 2019a). It remains to be determined whether other endosomal TLR signaling is terminated by a similar mechanism and, if so, by which E3 ubiquitin ligase.

Zinc and RING finger 1 (ZNRF1) belongs to the largest class of RING-finger E3 ligases in mammals and were originally identified in injury-induced nerve cells (Araki et al., 2001). ZNRF1 has been reported to localize in the endosome–lysosome compartments (Araki and Milbrandt, 2003). It has been shown that ZNRF1 promotes Wallerian degeneration through AKT degradation via the ubiquitin-proteasome system in response to oxidative stress (Wakatsuki et al., 2011). We revealed previously that ZNRF1 controls caveolin-1 ubiquitination and degradation to positively regulate TLR4-activated immune responses in vitro and in vivo (Lee et al., 2017). In addition, we showed recently that ZNRF1 mediates the ubiquitination of epidermal growth factor receptor (EGFR) and endocytic sorting to regulate EGFR signaling (Shen et al., 2021). In this study, we surprisingly found that ZNRF1 negatively regulates the endosomal TLR3-driven immune response by controlling TLR3 endocytic trafficking and degradation. We demonstrated that, upon TLR3 activation, c-Src activates ZNRF1 through phosphorylation of its 103rd tyrosine residue, and activated ZNRF1 associates with TLR3 and mediates K63-linked polyubiquitination at TLR3 K813 to promote receptor degradation via the lysosomal pathway. Mice and cells deficient in ZNRF1 are resistant to EMCV and SARS-CoV-2 infection because of prolonged TLR3 signaling and increased type I IFNs production. However, prolonged type I IFN responses by TLR3 activation render *Znrf1*^−/−^ mice more susceptible to opportunistic bacterial infection because of increased lung tissue damage. Our findings reveal a novel physiological function of ZNRF1 in controlling the termination of TLR3 signaling to prevent excessive type I IFN production and impairment of lung tissue repair.

## Results

### ZNRF1 negatively regulates TLR3-driven immune responses

**Figure S1.**
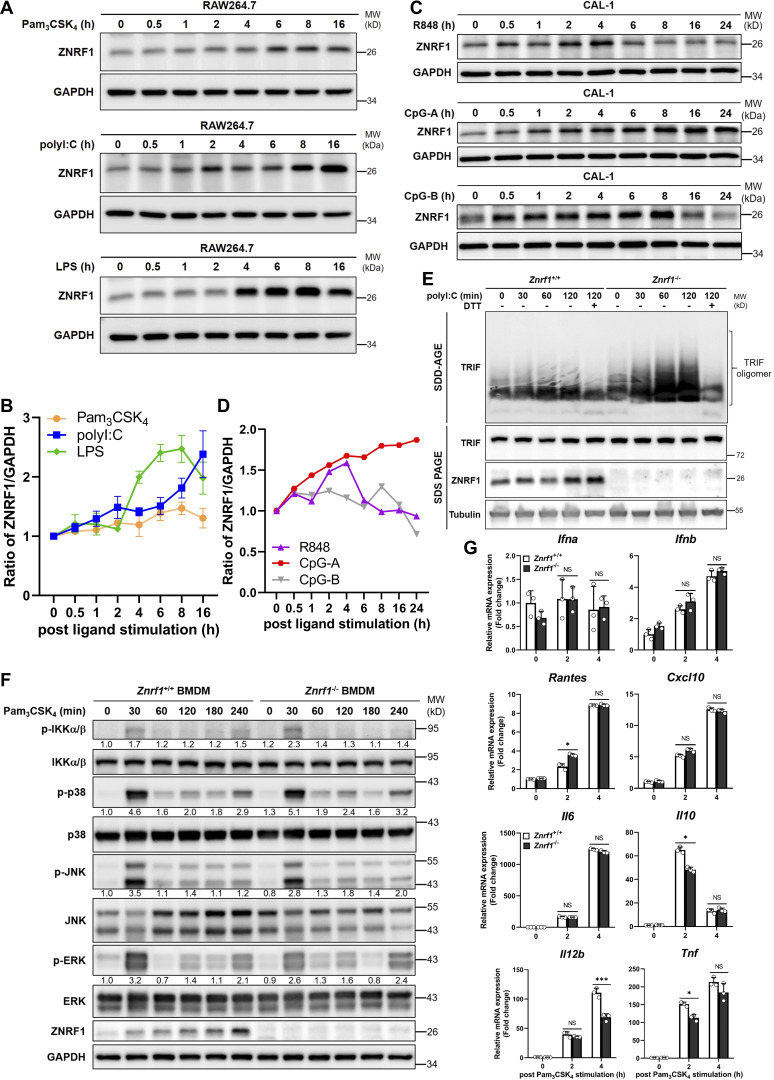
**ZNRF1 is not involved in TLR2-mediated immune responses. (A)** Immunoblot analysis of ZNRF1 protein in RAW264.7 cells after stimulation with Pam_3_CSK_4_ (1 μg/ml), poly(I:C) (30 μg/ml), and LPS (100 ng/ml) for the times indicated. **(B)** Quantification of ZNRF1 expression in A. The intensities of the ZNRF1 bands are expressed as fold increases compared with untreated control cells, after normalization to the internal control GAPDH. **(C)** Immunoblot analysis of ZNRF1 protein in CAL-1 cells after stimulation with R848 (2 μM), CpG-A (1 μM), and CpG-B (1 μM) for the times indicated. **(D)** Quantification of ZNRF1 protein expression in C as described in B. Data are representative of two independent experiments. **(E)** Cells were stimulated with poly(I:C) (30 μg/ml) for the times indicated. Cell lysates were collected in the presence or absence of dithiothreitol (DTT; 5 mM) and separated on semidenaturing detergent agarose gel electrophoresis (SDD-AGE) and 10% SDS-PAGE followed by immunoblotting with the indicated antibodies. **(F and G)** *Znrf1*^+/+^ and *Znrf1*^−/−^ BMDMs were stimulated with Pam_3_CSK_4_ (1 μg/ml) for the times indicated. The proteins (F) and mRNAs (G) indicated were analyzed by immunoblotting and RT-qPCR, respectively. The intensities of the bands are expressed as fold increases compared to untreated control cells, after normalization to their unphosphorylated forms. *P < 0.05 and ***P < 0.001 (Student’s *t* test). Data are representative of three independent experiments (error bars, mean ± SD). Source data are available for this figure: SourceData FS1.

**Figure 1.**
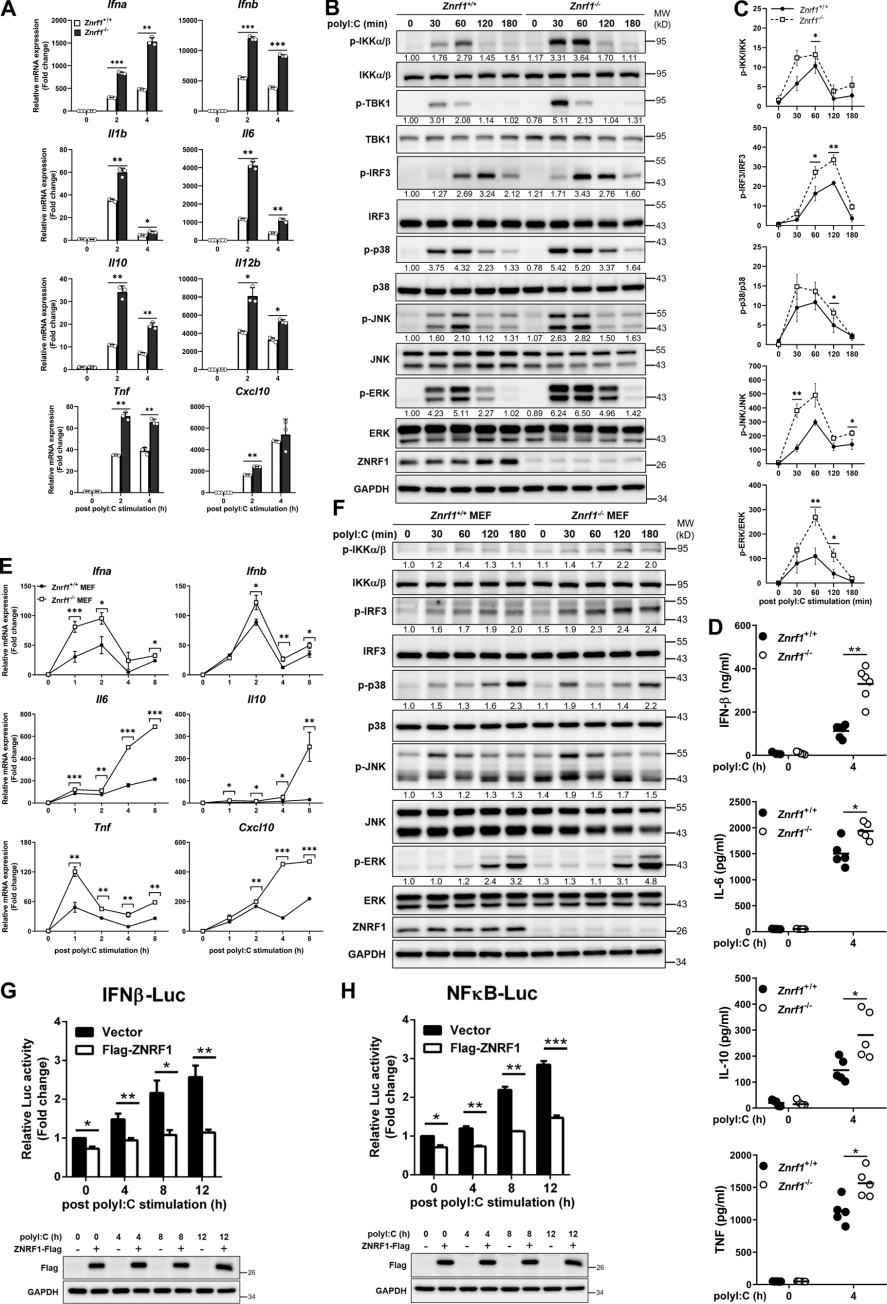
**ZNRF1 negatively regulates TLR3-mediated immune responses. (A and B)** BMDMs from *Znrf1*^+/+^ or *Znrf1*^−/−^ mice were treated with poly(I:C) (30 μg/ml) for the times indicated. **(A)** The expression of the indicated mRNAs was analyzed by RT-qPCR. **(B)** The phosphorylation of IKKα/β, IRF3, and MAPKs as well as the indicated proteins in cell lysates was analyzed by immunoblotting. The intensities of the bands are expressed as fold increases compared to those of untreated control cells after normalization to their unphosphorylated forms. **(C)** Quantification of immunoblotting analysis data of five independent experiments from B are shown. **(D)** The production of cytokines in the supernatants of *Znrf1*^+/+^ and *Znrf1*^−/−^ BMDMs at 4 h after poly(I:C) stimulation was determined by ELISA. **(E and F)** Primary *Znrf1*^+/+^ or *Znrf1*^−/−^ MEFs were stimulated with poly(I:C) (100 μg/ml) for the times indicated. **(E)** The expression of the indicated mRNAs was analyzed by RT-qPCR. **(F)** The levels of phosphorylation of IKKα/β, IRF3, MAPKs, and their unphosphorylated forms were analyzed by immunoblotting. **(G and H)** MEFs were cotransfected with IFN-β-Luc (G) or NF-κB-Luc (H) reporter and wild-type ZNRF1 for 24 h. Cells were stimulated with poly(I:C) (100 μg/ml) for the times indicated, followed by a dual-luciferase reporter assay. The expression of the proteins indicated in the cell lysates was confirmed by immunoblotting, as shown in the lower panel. *P < 0.05, **P < 0.01, and ***P < 0.001; NS, not significant (Student’s *t* test). Data are representative of three independent experiments (error bars, mean ± SD). Source data are available for this figure: SourceData F1.

**Figure. S2.**
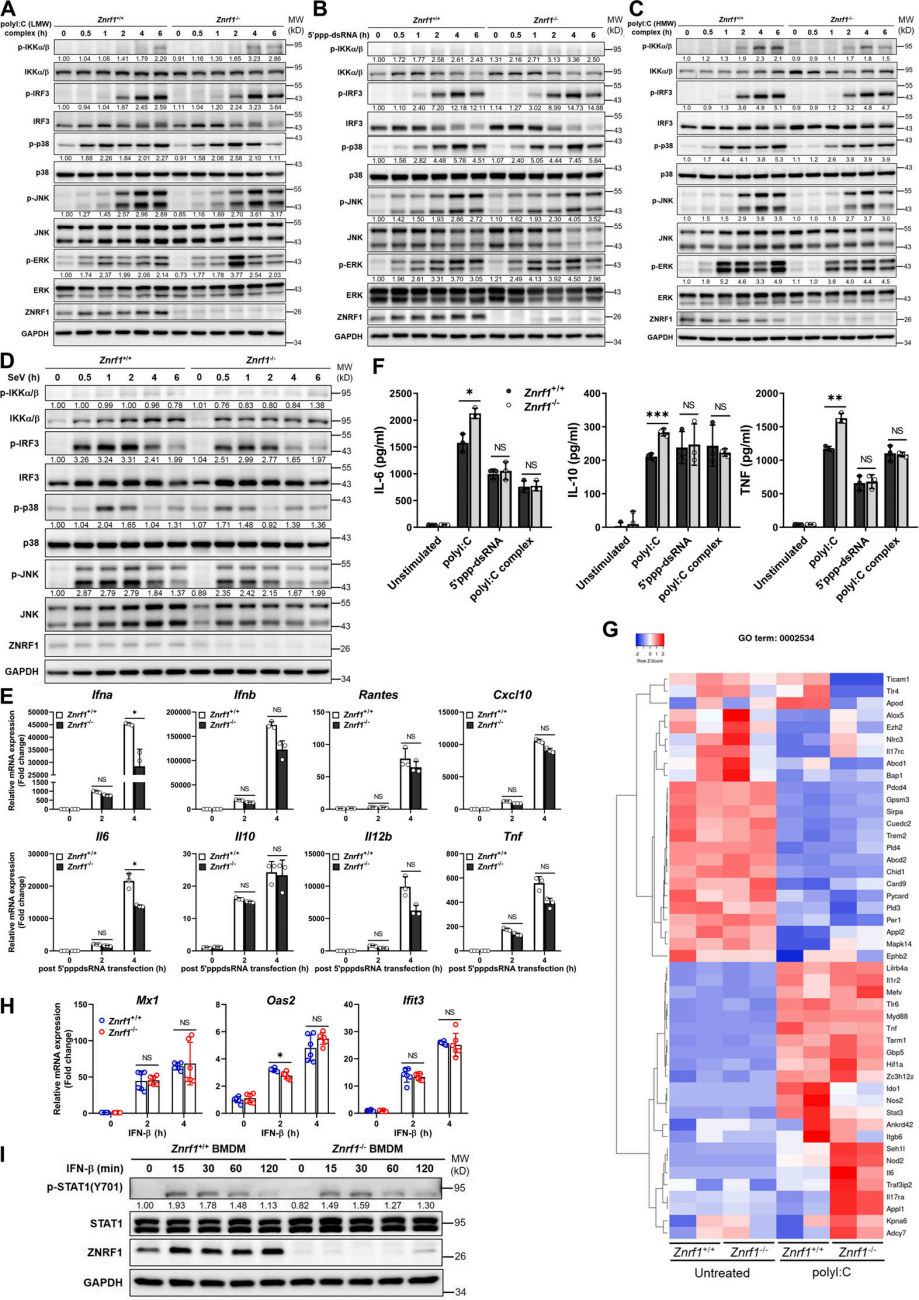
**ZNRF1 is not involved in RLR-mediated antiviral signaling or type I IFN–triggered immune responses. (A–E)** *Znrf1*^+/+^ and *Znrf1*^−/−^ BMDMs were transfected with poly(I:C) (low molecular weight; LMW; 2.5 μg/ml), poly(I:C) (HMW; 2.5 μg/ml), or 5′ppp-dsRNA (2.5 μg/ml) for the times indicated. **(A–C)** Immunoblot analysis of p-IKKα/β, p-IRF3, and phosphorylation of MAPKs in cell lysates. **(D)** Immunoblot analysis of p-IKKα/β, p-IRF3, and phosphorylation of MAPKs in *Znrf1*^+/+^ and *Znrf1*^−/−^ BMDMs, after infection with Sendai virus (SeV; 100 HA units/ml) for the indicated times. The intensities of the bands are expressed as fold increases compared with those of untreated control cells after normalization to their unphosphorylated forms. **(E)** The mRNA expression of the indicated genes was analyzed by RT-qPCR in *Znrf1*^+/+^ and *Znrf1*^−/−^ BMDMs after transfection of 5′ppp-dsRNA (2.5 μg/ml) for the times indicated. **(F)** The secretory levels of IL-6, IL-10, and TNF in supernatants of *Znrf1*^+/+^ and *Znrf1*^−/−^ BMDMs stimulated with poly(I:C) (30 μg/ml) or transfected with 5′ppp-dsRNA (2.5 μg/ml) or poly(I:C) (HMW; 2.5 μg/ml) for 16 h were detected by ELISA. **(G)** Heatmap showing the changes in cytokine production involved in the inflammatory response (GO term: 0002534) in BMDMs from *Znrf1*^+/+^ and *Znrf1*^−/−^ mice after 4 h treatment with poly(I:C) (30 μg/ml). **(H and I)** *Znrf1*^+/+^ and *Znrf1*^−/−^ BMDMs were stimulated with IFN-β (50 ng/ml) for the times indicated. **(H)** The mRNA expression of *Ifit3*, *Mx1*, and *Oas2* was analyzed by RT-qPCR. **(I)** Phosphorylation of STAT1 and STAT1 in cell lysates was determined by immunoblotting. The intensities of the bands are expressed as fold increases compared to those of untreated control cells, after normalization to their unphosphorylated forms. *P < 0.05, **P < 0.01, and ***P < 0.001 (Student’s *t* test). Data are representative of three independent experiments (error bars, mean ± SD). Source data are available for this figure: SourceData FS2.

**Figure 2.**
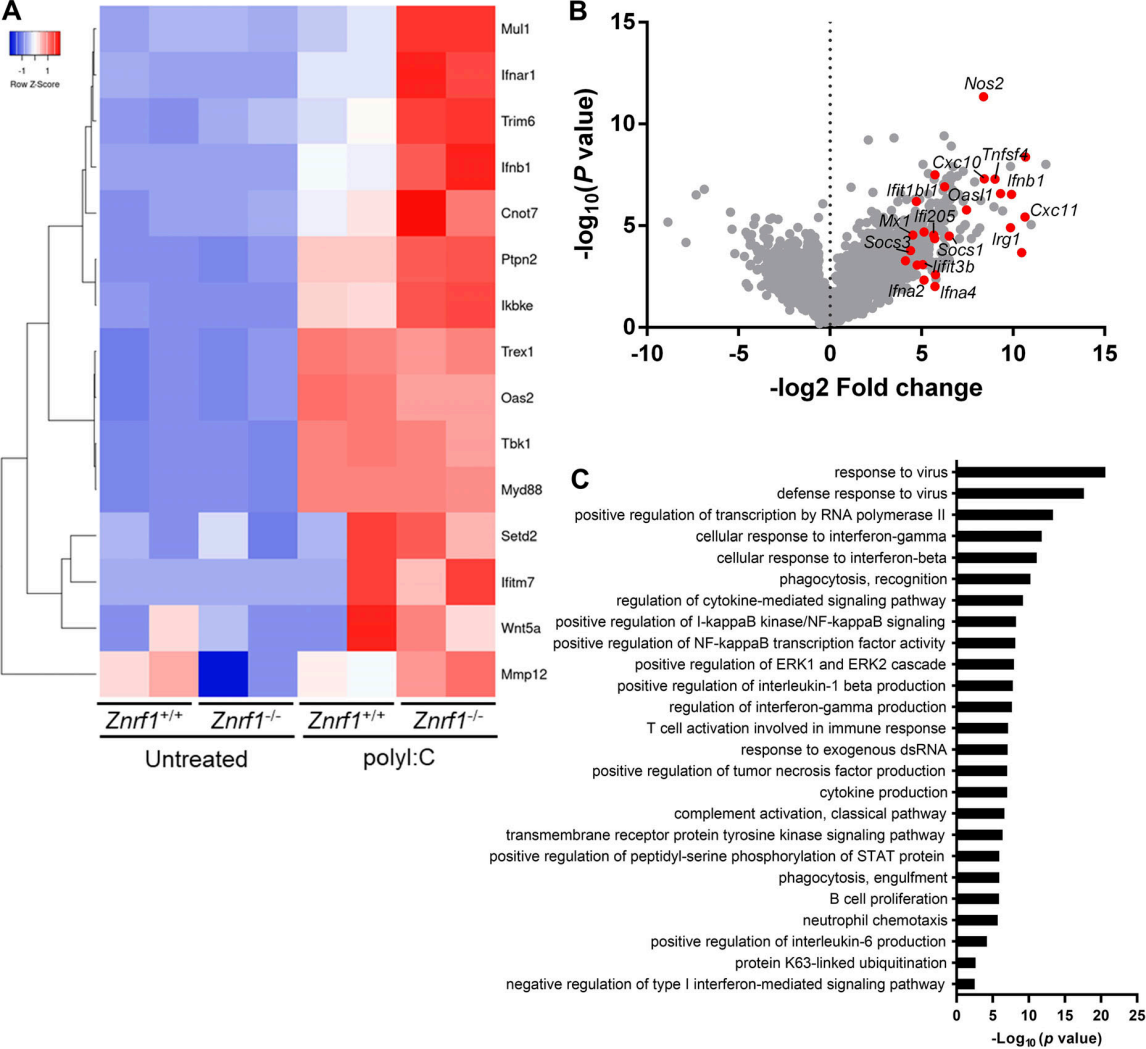
**Transcriptomic profiling reveals TLR3-driven IFN-stimulated gene expression influenced by ZNRF1 in BMDMs. (A)** Heatmap showing the changes of type I IFN–related gene expression in *Znrf1*^+/+^ and *Znrf1*^−/−^ BMDMs after 4 h treatment with poly(I:C) (30 μg/ml). **(B)** Volcano plot of the changes of differential gene expression in *Znrf1*^−/−^ BMDMs after 4 h treatment with 30 μg/ml poly(I:C) against wild-type BMDMs. The x axis indicates the logarithm of P values to the base 2 of the fold change, and the y axis reveals the negative logarithm of that to the base 10. Red dots denote transcripts related to type I IFN production in response to poly(I:C). **(C)** GO analysis of the differential expressed genes from B.

We reported recently that ZNRF1 positively regulates TLR4-driven immune responses by mediating caveolin-1 ubiquitination and degradation (Lee et al., 2017). However, we noted that depletion of ZNRF1 in macrophages increased LPS-induced IRF3 activation, which is mainly controlled by endosomal TLR4–TRIF signaling. Interestingly, ZNRF1 protein expression was significantly induced by poly(I:C) and LPS (ligands for TLR3 and TLR4) in RAW264.7 macrophages and R848, and CpG (ligands for TLR7 and TLR9) in CAL-1 cells, human plasmacytoid dendritic cell line (Fig. S1, A–D). This spurred us to investigate the impact of ZNRF1 on inflammatory responses mediated by endosomal TLRs. We chose to study TLR3 signaling to address this issue. We found that ZNRF1 depletion in bone-marrow-derived macrophages (BMDMs) significantly increased the mRNA expression of type I IFNs, such as *Ifna* and *Ifnb*, and proinflammatory cytokines, including *Il1b*, *Il6*, *Il10*, *Il12b*, *Tnf*, and *Cxcl10*, in response to poly(I:C) (Fig. 1 A). Consistent with the mRNA expression, the levels of cytokines, including IFN-β, IL-6, IL-10, and TNF, were increased in ZNRF1-depleted BMDMs after poly(I:C) treatment (Fig. 1 D). We then assessed upstream TLR signaling and found that activation of IKK, IRF3, and MAPKs, including p38, JNK, and ERK, was increased in *Znrf1*^−/−^ BMDM in response to poly(I:C) (Fig. 1, B and C). Similar results were observed in *Znrf1*^−/−^ murine embryonic fibroblasts (MEFs), non-immune cells (Fig. 1, E and F), suggesting that the ZNRF1-mediated TLR3 immune response is not cell specific. Once TLR3 is activated by ligands, TRIF is recruited to TLR3 and undergoes oligomerization (Zang et al., 2020). We performed semidenaturing detergent agarose gel electrophoresis to assess whether ZNRF1 modulates TRIF oligomerization. Deficiency of ZNRF1 enhanced TRIF oligomerization in response to poly(I:C), indicating a negative regulatory role of ZNRF1 in TLR3 signaling (Fig. S1 E). Consistent with its suppressive effect, luciferase reporter assays revealed that overexpression of ZNRF1 in MEFs substantially decreased the luciferase activity of both IFN-β and NF-κB–containing promoters upon poly(I:C) stimulation (Fig. 1, G and H). TLR2 localizes at the plasma membrane, where it initiates the immune response through the adaptor proteins MyD88 and Mal (Takeuchi and Akira, 2010). Unlike its role in TLR3-mediated responses, ZNRF1 was not involved in TLR2-driven inflammatory responses (Fig. S1, F and G). RIG-I–like receptors (RLRs), including retinoic acid-inducible gene I (RIG-I) and melanoma differentiation-associated protein 5 (MDA5), and cytosolic pattern recognition receptors can sense intracellular poly(I:C) and initiate inflammation. To assess the involvement of ZNRF1 in the regulation of RLR-mediated immune responses, we transfected control and *Znrf1*^−/−^ BMDMs with high molecular (HMW) or low molecular weight poly(I:C) (specific ligands for MDA5 and RIG-I, respectively) or 5′ppp-dsRNA. In contrast to its function in TLR3 responses, ZNRF1 was not required for RLR-induced signaling (Fig. S2, A–C). Similarly, ZNRF1 was dispensable for the activation of IKK and MAPKs in macrophages challenged with Sendai virus, which mainly triggers RIG-I signaling (Kato et al., 2006; Fig. S2 D). Accordingly, the mRNA expression of cytokines and type I IFNs, as well as the release of cytokines, were comparable between control and *Znrf1*^−/−^ BMDMs transfected with poly(I:C) or 5′ppp-dsRNA (Fig. S2, E and F). To evaluate comprehensively the effect of ZNRF1 on TLR3 activation, we performed RNA sequencing analysis of control and *Znrf1*^−/−^ BMDMs challenged with poly(I:C). Among the poly(I:C)-induced genes, elevated expression of 2,840 genes, including type I IFNs and interferon-stimulated genes, was observed in *Znrf1*^−/−^ BMDM (Fig. 2, A and B; and Fig. S2 G). Gene ontology (GO) enrichment analysis revealed that genes involved in virus defenses and IFN responses were upregulated in poly(I:C)-stimulated *Znrf1*^−/−^ BMDMs (Fig. 2 C), suggesting systemic upregulation of type I IFN responses in *Znrf1*^−/−^ BMDMs. However, the IFN-β–driven immune response was comparable in wild-type and *Znrf1*^−/−^ BMDMs (Fig. S2, H and I), indicating that ZNRF1 is not involved in type I IFN signaling. Together, we discovered that ZNRF1 negatively regulates TLR3-mediated immune responses.

### ZNRF1-deficient cells are resistant to EMCV and SARS-CoV-2 infection

**Figure 3.**
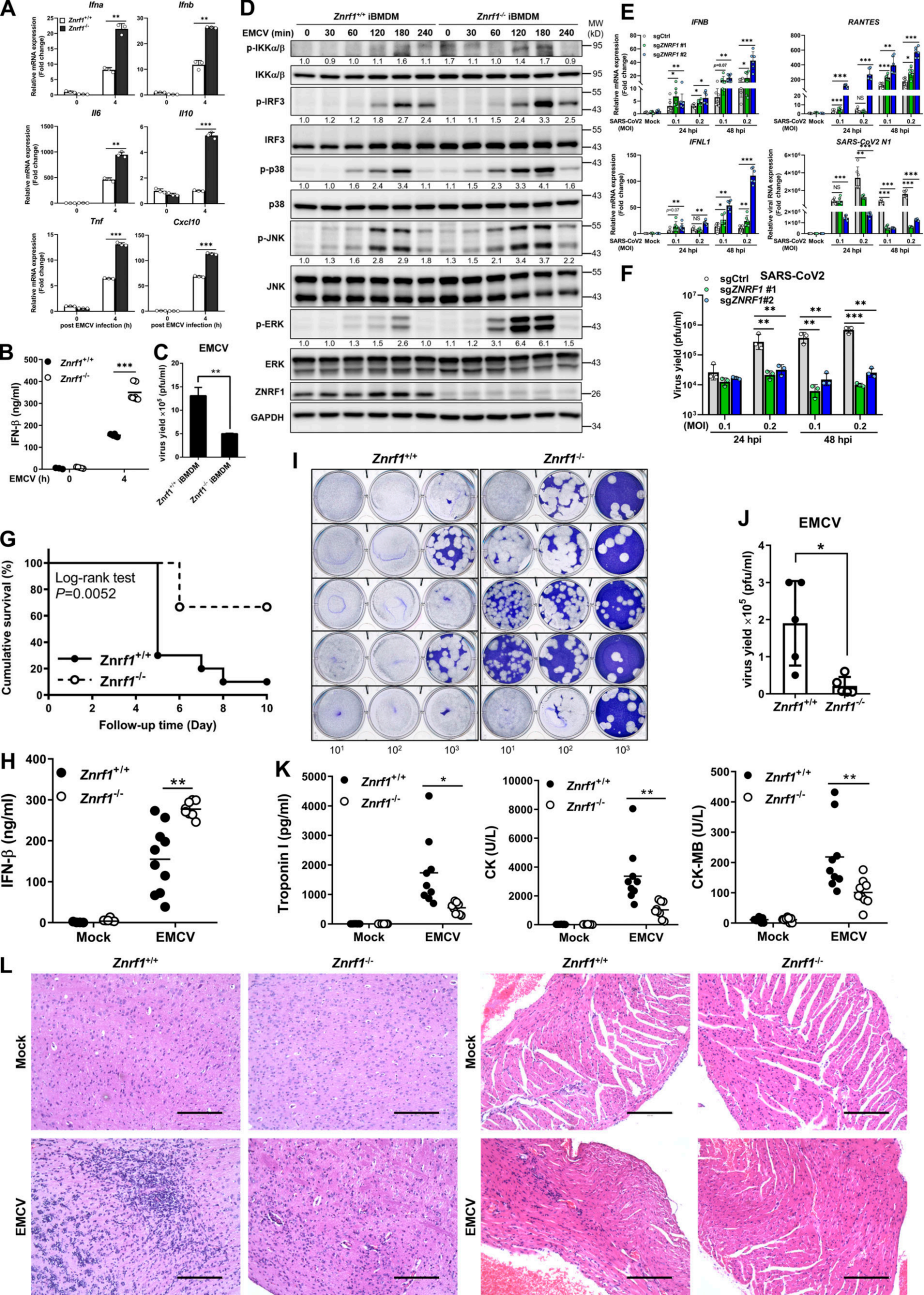
**ZNRF1-deficient cells and mice are resistant to EMCV and SARS-CoV-2 infection. (A–D)** Wild-type and *Znrf1*^−/−^ iBMDMs were infected with EMCV (MOI = 10) for 4 h (A and B), 24 h (C), or the times indicated (D). **(A)** The expression of the indicated mRNAs was analyzed by RT-qPCR. **(B)** The level of IFN-β in culture supernatant was quantified by ELISA. **(C)** Plaque assay of EMCV in culture supernatants of infected BMDMs. **(D)** The levels of phosphorylation of IKKα/β, IRF3, MAPKs, and their unphosphorylated forms were analyzed by immunoblotting. **(E and F)** Wild-type and *ZNRF1*^−/−^ Calu-3 cells were infected with SARS-CoV-2 at an MOI of 0.1 and 0.2 for 24 and 48 h. **(E)** Total RNAs were prepared, and the levels of *IFNB*, *IFNL1*, and *RANTE* mRNAs, as well as *SARS-CoV-2 N1* RNA, were quantified by RT-qPCR. **(F)** Plaque assay of SARS-CoV-2 in culture supernatants of infected Calu-3 cells. **(G)** Survival of *Znrf1*^+/+^ (solid line) and *Znrf1*^−/−^ (dotted line) mice (*n* = 9 per genotype) given i.p. injection of EMCV (10^4^ pfu per mouse). **(H–K)** *Znrf1*^+/+^ and *Znrf1*^−/−^ mice were injected i.p. with EMCV (10^7^ pfu per mouse) for 72 h. **(H)** ELISA analysis of IFN-β in sera from *Znrf1*^+/+^ and *Znrf1*^−/−^ mice after EMCV challenge. **(I)** Plaque assay of EMCV in the brain tissues of infected mice (*n* = 5 per genotype). **(J)** Quantification of virus titers from I. **(K)** ELISA analysis of the levels of Troponin-I, CK, and CK-MB in sera from *Znrf1*^+/+^ and *Znrf1*^−/−^ mice upon EMCV infection (*n* = 9 per genotype). L, liter. **(L)** H&E staining of histological sections of brain and heart tissues collected from *Znrf1*^+/+^ and *Znrf1*^−/−^ mice 4 d after EMCV infection (10^4^ pfu per mouse) and from mock-infected control mice. Objective magnification, ×20. Scale bar, 200 μm. *P < 0.05, **P < 0.01, and ***P < 0.001 (Student’s *t* test). Data (except L) are representative of three independent experiments (error bars, mean ± SD). Source data are available for this figure: SourceData F3.

**Figure S3.**
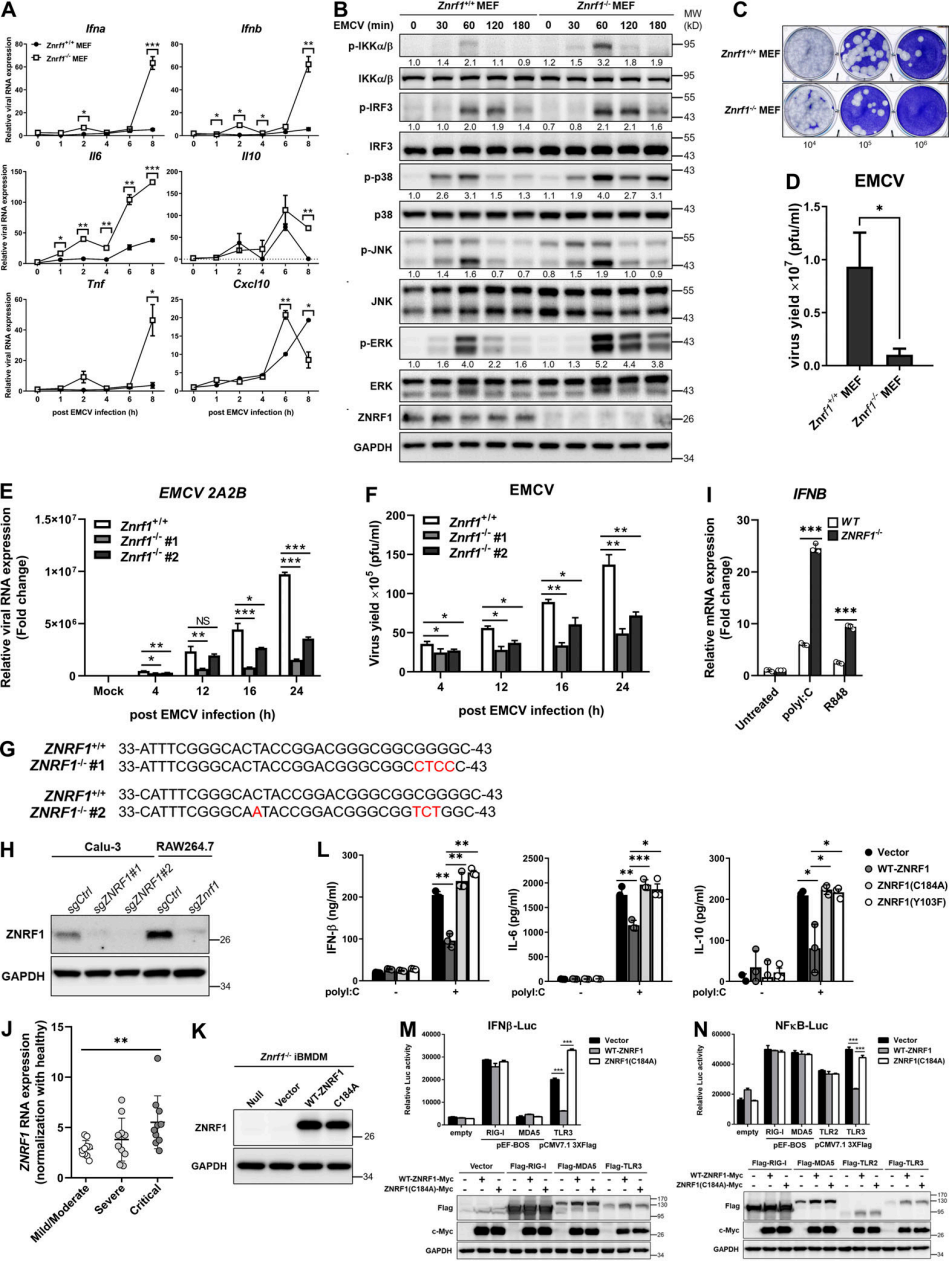
**ZNRF1 deficiency in MEFs and BMDMs enhances IFN production and restricts EMCV proliferation after viral infection. (A–D)** *Znrf1*^+/+^ and *Znrf1*^−/−^ MEFs were infected with EMCV at MOI of 1 for the times indicated. **(A)** RT-qPCR analysis of the mRNA expression of the genes indicated. **(B)** Immunoblot analysis of p-IKKα/β, p-IRF3, and phosphorylation of MAPKs in *Znrf1*^+/+^ and *Znrf1*^−/−^ MEFs. **(C)** The viral titers in culture media were determined by plaque assays. **(D)** Quantification of viral particles in C. **(E and F)** *Znrf1*^+/+^ and *Znrf1*^−/−^ BMDMs were infected with EMCV at an MOI of 5 for the times indicated. **(E)** RT-qPCR analysis of the expression of *EMCV 2A2B* RNA in BMDMs. **(F)** Quantification of viral particles in culture media was made by plaque assays. **(G)** Sequence analysis of wild-type and two different *ZNRF1*^−/−^ Calu-3 clones generated by the CRISPR/Cas9 system. Genomic DNA was extracted from wild-type and *ZNRF1*^−/−^ Calu-3 cells and the region surrounding the targeted site was amplified by PCR for sequencing. Indel mutations are indicated in red. **(H)** Immunoblot analysis of ZNRF1 protein in cell lysates from scrambled controls (sgCtrl) and *ZNRF1*^−/−^ Calu-3 and RAW264.7 cells. **(I)** Calu-3 cells treated with poly(I:C) (30 μg/ml) or R848 (2 μM) for 4 h. RT-qPCR analysis of the expression of *IFNB* in Calu-3 cells. **(J)** The RNA level of *Znrf1* in patients with mild-to-moderate (*n* = 10), severe (*n* = 10), and critical (*n* = 10) COVID-19 were normalized with healthy controls for clinical validation. Data were analyzed from the National Center for Biotechnology Information GEO database, accession number: GSE167930. **(K)** Immunoblot analysis of ZNRF1 protein in lysates from *Znrf1*^−/−^ iBMDMs reconstituted with vector, wild-type ZNRF1, or ZNRF1(C184A) mutant. **(L)** The secretion of IFN-β, IL-6, and IL-10 into the culture media of *Znrf1*^−/−^ RAW264.7 cells reconstituted with vector, wild-type ZNRF1, ZNRF1(C184A), or ZNRF1(Y103F) mutants after stimulation with poly(I:C) (30 μg/ml) for 4 h were measured by ELISA. **(M and N)** HEK293T were co-transfected with IFN-β-Luc (M) or NF-κB-Luc (N) reporter, wild-type ZNRF1 or ZNRF1(C184A) mutant, and the pattern-recognition receptors indicated for 36 h. Cells were harvested, and reporter activities were analyzed by the dual-luciferase reporter assay. The expression of the proteins indicated in cell lysates was confirmed by immunoblotting, as shown in the lower panel. *P < 0.05, **P < 0.01, and ***P < 0.001 (Student’s *t* test). Data are representative of three independent experiments (error bars, mean ± SD). Source data are available for this figure: SourceData FS3.

TLR3 is crucial for the host to sense invading RNA viruses and to trigger antiviral immunity (Takeuchi and Akira, 2010). To investigate the functional role of ZNRF1 in mammalian antiviral immunity, we infected macrophages generated from immortalized macrophage progenitors (iBMDMs) with EMCV, which has been shown to induce host innate immunity, mainly through TLR3 and MDA5 (Gitlin et al., 2006; Hardarson et al., 2007). Similar to poly(I:C), EMCV infection elevated the mRNA expression of cytokines and type I IFNs, including *Ifna*, *Ifnb*, *Il6*, *Il10*, *Tnf*, *Cxcl10*, as well as IFN-β production in *Znrf1*^−/−^ cells compared with wild-type cells (Fig. 3, A and B). Consistent with augmenting type I IFN production, *Znrf1*^−/−^ iBMDM produced significantly less EMCV than control cells (Fig. 3 C). Activation of IKK, MAPKs, and IRF3 was also increased in EMCV-challenged *Znrf1*^−/−^ iBMDM (Fig. 3D). Similar results were obtained from MEFs (Fig. S3, A–D) and primary BMDMs (Fig. S3, E and F), as cells with depletion of ZNRF1 showed enhanced activation of IKK and IRF3, increased type I IFN and cytokine production, and less EMCV proliferation. Recent studies revealed that patients bearing genetic mutations in TLR3 and TLR7 signaling experienced severe outcomes of coronavirus disease 2019 (COVID-19; Asano et al., 2021; Zhang et al., 2020), suggesting the crucial protective function of TLR3/7 signaling against SARS-CoV-2 infection. To explore whether ZNRF1 participates in the innate immune response against SARS-CoV-2 infection, we deleted the *ZNRF1* gene in human lung epithelial Calu-3 cells using the CRISPR/Cas9 system (Fig. S3, G and H). Consistently, poly(I:C) induced more type I IFN (*IFNB*) expression in *ZNRF1*^−/−^ Calu-3 cells (Fig. S3 I). Compared with poly(I:C), the TLR7 ligand, R848, was a weaker inducer of *IFNB* expression in Calu-3 cells (Fig. S3 I), which is consistent with the previous reports that human TLR7 is mainly expressed in plasmacytoid dendritic cells (Laurent et al., 2022; van der Sluis et al., 2022). In line with its effect on TLR3 signaling, depletion of ZNRF1 in Calu-3 cells induced more mRNA expression of type I IFN (*IFNB*), type III IFN (*IFNL1*), and IFN-inducible gene (*RANTES*) than wild-type cells (Fig. 3 E). Similarly, cells with deletion of ZNRF1 expressed less non-structural viral gene N1 RNA (Fig. 3 E) and produced less SARS-CoV-2 (Fig. 3 F). Interestingly, ZNRF1 expression in peripheral blood mononuclear cells was positively correlated with the severity of COVID-19 (Fig. S3 J). Our findings together suggest that ZNRF1 negatively regulates TLR3-mediated antiviral immunity.

### Mice with ZNRF1 deficiency have greater resistance to EMCV infection

To investigate further the physiological function of ZNRF1 in TLR3-mediated antiviral immunity, age- and sex-matched *Znrf1*^−/−^ mice and their wild-type littermates were infected i.p. with EMCV. Infection of mice with EMCV is known to lead to myocarditis, with severe inflammation of the heart and brain, and TLR3 signaling is crucial for the host response to EMCV infection (Hardarson et al., 2007). After EMCV challenge, more than 60% of wild-type mice died within 5 d, whereas more than 60% of the *Znrf1*^−/−^ mice survived for up to 6 d (Fig. 3 G). The serum concentrations of IFN-β in *Znrf1*^−/−^ mice were significantly higher than those of their wild-type littermates (Fig. 3 H). Consistently, EMCV replication was decreased in the brain tissues of *Znrf1*^−/−^ mice compared with those of wild-type mice (Fig. 3, I and J). In addition, the serum levels of troponin-I, creatine kinase (CK), and CK-MB, all of which are biomarkers of myocarditis, were lower in *Znrf1*^−/−^ mice (Fig. 3 K). Histological staining revealed attenuated inflammation and tissue damage in the hearts and brain tissues of *Znrf1*^−/−^ mice during EMCV infection (Fig. 3 L). Altogether, these results demonstrate that ZNRF1 is a critical regulator of TLR3-mediated antiviral immunity that has significant impacts on mortality and virus replication.

### ZNRF1 requires its ubiquitin ligase activity to regulate TLR3-driven immune responses

**Figure 4.**
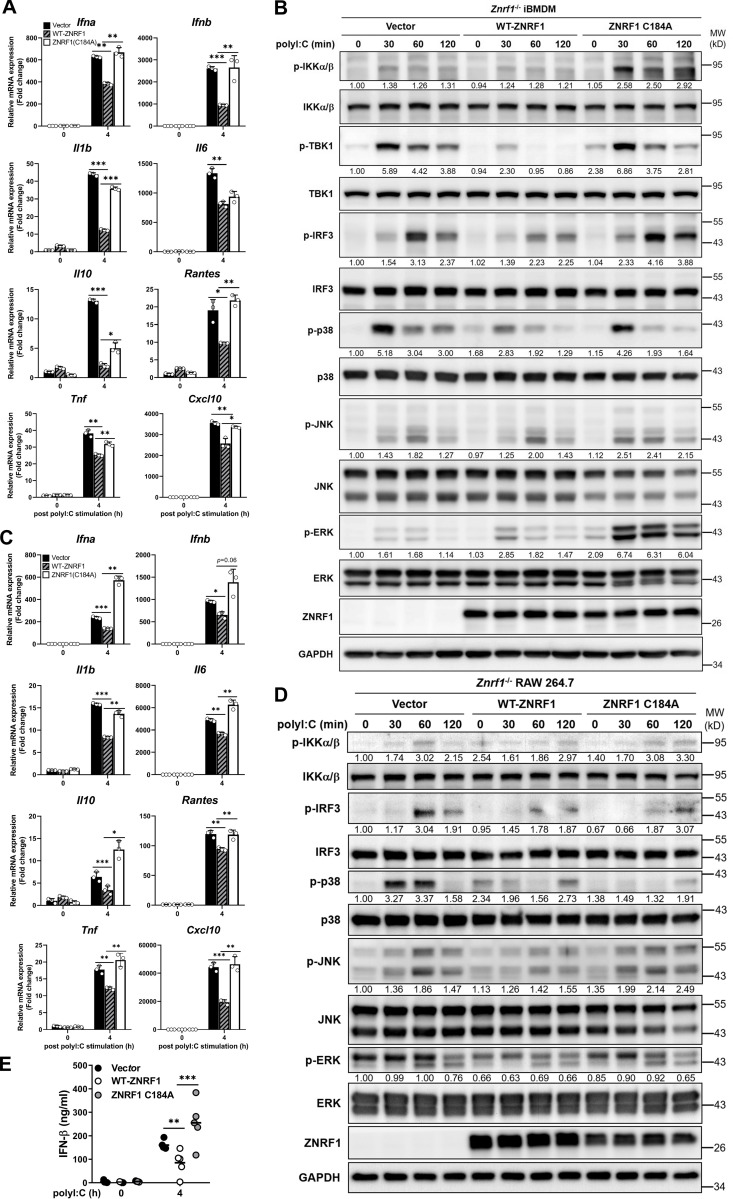
**ZNRF1-mediated TLR3-driven immune responses require its E3 ubiquitin ligase activity. (A–E)** *Znrf1*^−/−^ iBMDMs (A and B) or RAW264.7 cells (C–E) were reconstituted with Tet-inducible vector, wild-type ZNRF1, and ZNRF1(C184A) mutant, and stimulated with poly(I:C) (30 μg/ml) for the times indicated. **(A and C)** The mRNA levels of the indicated type I IFN–related genes and proinflammatory cytokines were detected by RT-qPCR. **(B and D)** The phosphorylation of IKKα/β, IRF3, MAPKs, and the indicated proteins in cell lysates was analyzed by immunoblotting. The intensities of the bands are expressed as fold increases compared to those of untreated control cells, after normalization to their unphosphorylated forms. **(E)** The level of IFN-β in culture supernatants was measured by ELISA. *P < 0.05, **P < 0.01, and ***P < 0.001 (Student’s *t* test). Data are representative of three independent experiments (error bars, mean ± SD). Source data are available for this figure: SourceData F4.

Cysteine 184 of ZNRF1 is critical for its E3 ubiquitin ligase activity (Araki and Milbrandt, 2003; Lee et al., 2017). To determine whether this activity is necessary for the modulation of TLR3-driven immunity, we reconstituted *Znrf1*^−/−^ iBMDMs with inducible wild-type ZNRF1 or a catalytically inactive E3 ligase mutant ZNRF1(C184A) (Fig. S3 K). Reconstitution of *Znrf1*^−/−^ iBMDMs or RAW264.7 with ZNRF1, but not its enzymatically inactive mutant C184A, reduced poly(I:C)-induced phosphorylation of IKK, IRF3, and MAPKs, as well as transcription of the *Ifna*, *Ifnb*, *Il1b*, *Il6*, *Il10*, *Rantes*, *Tnf*, and *Cxcl10* genes (Fig. 4, A–D). Consistently, secretion of IFN-β, IL-6, and IL-10 by *Znrf1*^−/−^ macrophages was decreased after reconstitution with ZNRF1 but not by those reconstituted with the ZNRF1(C184A) mutant (Fig. 4 E and Fig. S3 L). In addition, ectopic expression of wild-type ZNRF1 suppressed IFN-β– and NF-κB–driven reporter luciferase activities induced by TLR3 compared with the vector or ZNRF1(C184A) mutant (Fig. S3, M and N). In line with the previous results, ZNRF1 had no effect on TLR2-, RIG-I–, or MDA5-mediated IFN-β– and NF-κB–driven reporter activities. These results suggest that the E3 ubiquitin ligase activity of ZNRF1 is indispensable for its regulation of TLR3-driven signaling.

### ZNRF1 promotes TLR3 trafficking from endolysosomes to MVBs/lysosomes for degradation

**Figure S4.**
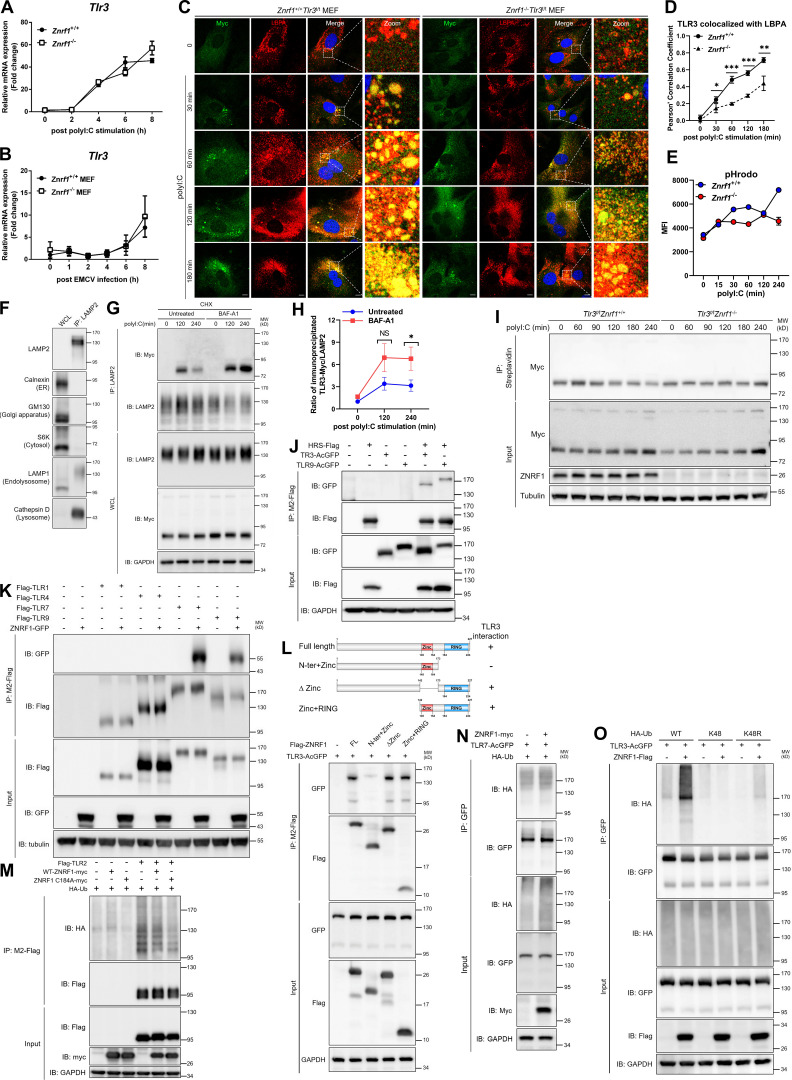
**ZNRF1 does not affect TLR3 mRNA expression upon ligand binding and does not mediate K48-linked polyubiquitin chains on TLR3. (A and B)** RT-qPCR analysis of the mRNA expression of *Tlr3* in BMDMs and MEFs from *Znrf1*^+/+^ and *Znrf1*^−/−^ mice after stimulation with poly(I:C) (30 μg/ml) or EMCV at an MOI of 1 for the times indicated. **(C and D)** *Znrf1*^+/+^*Tlr3*^t/t^ and *Znrf1*^−/−^*Tlr3*^t/t^ MEFs were not treated or treated with poly(I:C) (100 μg/ml) for the times indicated. **(C)** Cells were costained with antibodies against Myc (TLR3) and LBPA. Scale bar, 10 μm. **(D)** Quantitative analysis of colocalization of TLR3 with LBPA. **(E)** *Znrf1*^+/+^ and *Znrf1*^−/−^ BMDMs were stimulated with poly(I:C) (30 μg/ml) for the times indicated. Cells were incubated with pHrodo green for 15 min followed by flow cytometric analysis. MFI, mean fluorescence intensity. **(F)** Cell lysates were prepared from BMDMs and immunoprecipitated with LAMP2 antibodies. **(G and H)** WCL and purified LAMP2^+^ vesicles were subjected to immunoblotting using the antibodies against proteins of various subcellular compartments (Calnexin: ER, GM130: Golgi apparatus, S6K: cytosol, LAMP1: endolysosomes/lysosomes, and Cathepsin D: lysosomes; G and H) *Znrf1*^+/+^*Tlr3*^t/t^ BMDMs were pretreated with CHX (10 μg/ml) for 1 h and then stimulated with poly(I:C) (30 μg/ml) for 30 min, followed by treatment with Bafilomycin A1 (BAF-A1; 2 μM) for the times indicated. Cell lysates were prepared and immunoprecipitated with LAMP2 antibodies followed by immunoblotting (IB) with the antibodies indicated (G). The intensities of the immunoprecipitated Myc bands are expressed as fold increases compared to those of untreated control cells, after normalization to their immunoprecipitated LAMP2 (H). **(I)** *Znrf1*^+/+^*Tlr3*^t/t^ and *Znrf1*^−/−^*Tlr3*^t/t^ BMDMs were methionine-starved for 1 h, and then fed with L-Azidohomoalanine for 4 h, followed by poly(I:C) (30 μg/ml) stimulation for the times indicated. Cell lysates were crosslinked with Biotin-alkyne and then immunoprecipitated with anti-Streptavidin antibody, followed by immunoblotting with the antibodies indicated. **(J)** HEK293T were co-transfected with HRS-Flag, TLR3-AcGFP, or TLR9-AcGFP for 72 h, and cell lysates were immunoprecipitated with anti-M2-Flag antibody. Immunocomplexes and WCL were subjected to immunoblotting with the antibodies indicated. **(K)** HEK293T were cotransfected with ZNRF1-GFP or Flag-tagged TLR1 or TLR4 or TLR7 or TLR9 for 36 h, and cell lysates were immunoprecipitated with anti-M2-Flag antibody. Immunocomplexes and WCL were subjected to immunoblotting with the antibodies indicated. **(L)** HEK293T cells were cotransfected with AcGFP-tagged TLR3 and Flag-tagged full-length (FL) or truncated forms of ZNRF1 for 72 h, and the interaction between TLR3 and ZNRF1 was identified by immunoprecipitation followed by immunoblotting with the antibodies indicated. Schematic diagram of full-length ZNRF1 and its various deletion mutants, with a C-terminal Flag tag, is shown in the upper panel. **(M)** HEK293T cells were cotransfected with Flag-tagged TLR2, HA-tagged ubiquitin, and myc-tagged wild-type ZNRF1 or ZNRF1(C184A) for 36 h. **(N)** HEK293T cells were cotransfected with TLR7-AcGFP, ZNRF1-myc, and HA-tagged ubiquitin for 36 h. **(O)** HEK293T cells were cotransfected with TLR3-AcGFP, ZNRF1-Flag, and HA-tagged wild-type or ubiquitin mutants for 36 h. Cell lysates were immunoprecipitated using anti-GFP antibody. The immunocomplexes and WCL were analyzed by immunoblotting using the antibodies indicated. Data are representative of two independent experiments (error bars, mean ± SD). Source data are available for this figure: SourceData FS4.

**Figure 5.**
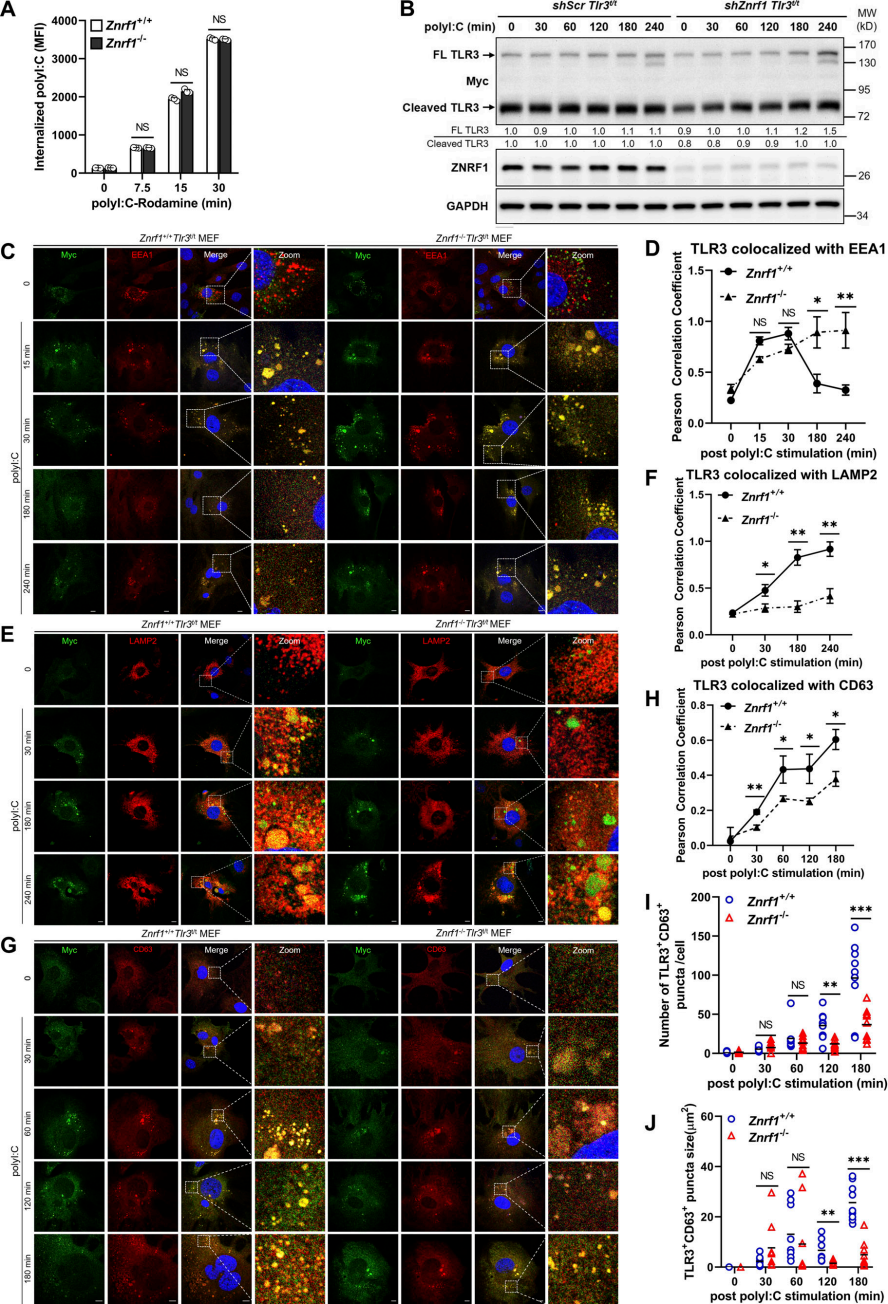
**ZNRF1 promotes TLR3 trafficking from endosomes to lysosomes. (A)** *Znrf1*^+/+^ and *Znrf1*^−/−^ BMDMs were treated with poly(I:C)-conjugated Rhodamine for the times indicated, followed by flow cytometric analysis. **(B)** *Tlr3*^t/t^ BMDMs expressing control shRNA (*shScr*) or *shZnrf1* were stimulated with poly(I:C) (30 μg/ml) for the times indicated. Cell lysates were prepared and subjected to immunoblotting with the antibodies indicated. The intensities of the full-length (FL) and cleaved TLR3 bands are expressed as fold increases compared with those of untreated control cells after normalization to their internal control GAPDH. **(C–J)** *Znrf1*^+/+^*Tlr3*^t/t^ and *Znrf1*^−/−^*Tlr3*^t/t^ MEFs were untreated or treated with poly(I:C) (100 μg/ml) for the times indicated. Cells were costained with antibodies against Myc (TLR3) and EEA1 (C and D), LAMP2 (E and F), or CD63 (G–J). Quantitative analysis of colocalization of TLR3 with EEA1, LAMP2, or CD63 (D, F, and H–J). The numbers of TLR3^+^CD63^+^ puncta with an area >0.2 μm^2^ (I) and puncta size (J) in G. Colocalization coefficients of TLR3 and EEA1, TLR3 and LAMP2, and TLR3 and CD63 were respectively quantified in at least three different images (dozens of cells) using ImageJ software. *P < 0.05, **P < 0.01, and ***P < 0.001. (Student’s *t* test). Data are representative of three independent experiments (error bars, mean ± SD). Source data are available for this figure: SourceData F5.

**Figure 6.**
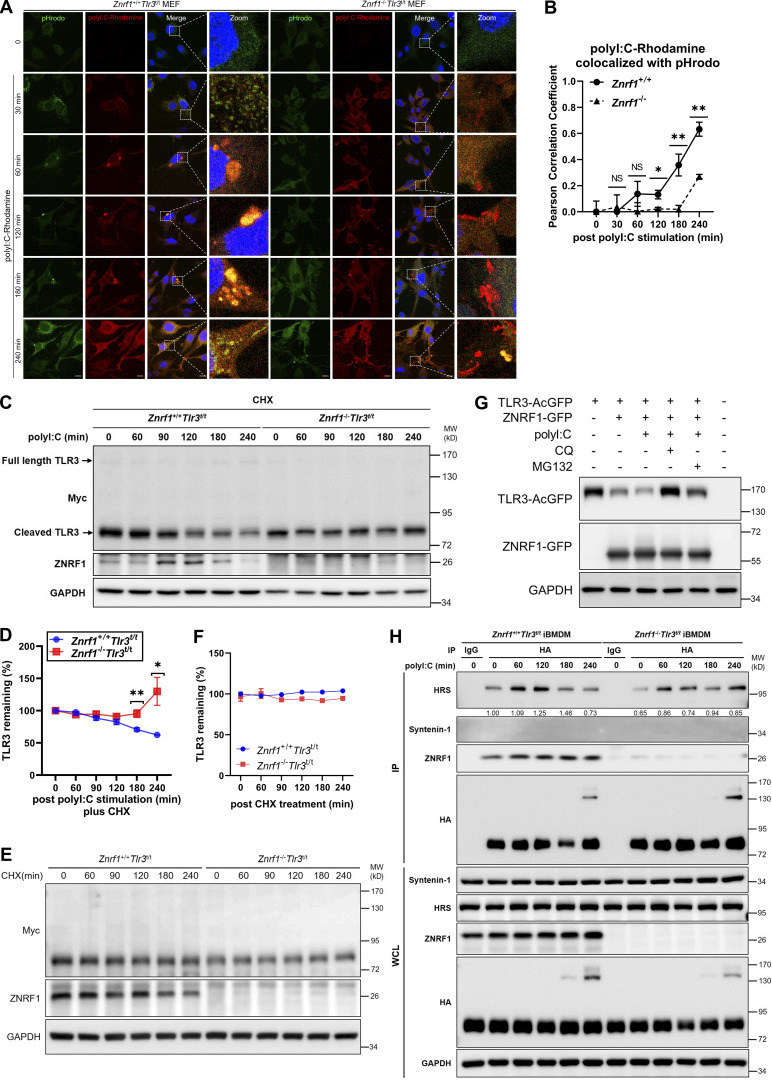
**ZNRF1 promotes lysosomal-dependent degradation of TLR3. (A)** *Znrf1*^+/+^*Tlr3*^t/t^ and *Znrf1*^−/−^*Tlr3*^t/t^ MEFs treated with poly(I:C) conjugated Rhodamine for the times indicated were incubated with pHrodo green for 15 min followed by confocal microscopy. Scale bar, 10 μm. **(B)** Colocalization coefficients of poly(I:C)-Rhodamine and the pHrodo signal were quantified in at least three different images (dozens of cells) using ImageJ software. **(C and D)** *Znrf1*^+/+^*Tlr3*^t/t^ and *Znrf1*^−/−^*Tlr3*^t/t^ BMDMs were pretreated with CHX (10 μg/ml) for 1 h and then stimulated with poly(I:C) (30 μg/ml) for the times indicated. Cell lysates were immunoblotted with the antibodies indicated. Quantification of immunoblotting analysis data of three independent experiments is shown in D. The intensities of cleaved TLR3 bands are compared with those of untreated cells after normalization to GAPDH expression. **(E and F)** *Znrf1*^+/+^*Tlr3*^t/t^ and *Znrf1*^−/−^*Tlr3*^t/t^ BMDMs were pretreated with CHX (10 μg/ml) for 1 h and then the cell lysates were harvested at the times indicated, followed by immunoblotting. Quantification of immunoblotting analysis data is shown in F. **(G)** MEFs expressing either vector or TLR3-AcGFP with ZNRF1-GFP were stimulated with poly(I:C) (100 μg/ml) for 6 h, followed by treatment with chloroquine (CQ; 50 μM) or MG132 (10 μM) for 4 h. Cell lysates were harvested and subjected to immunoblotting with the antibodies indicated. **(H)** *Znrf1*^+/+^*Tlr3*^t/t^ and *Znrf1*^−/−^*Tlr3*^t/t^ BMDMs were untreated or treated with poly(I:C) (30 μg/ml) for the times indicated, and cells lysates were prepared and immunoprecipitated with anti-HA antibody. Whole-cell lysates (WCL) and the immunocomplexes were subjected to immunoblotting using the antibodies indicated. *P < 0.05, **P < 0.01. (Student’s *t* test). Data are representative of three independent experiments (error bars, mean ± SD). Source data are available for this figure: SourceData F6.

We then investigated the mechanisms underlying ZNRF1 regulation of TLR3 signaling. TLR3 mRNA expression was similar in wild-type and *Znrf1*^−/−^ cells with or without challenge with poly(I:C) and EMCV (Fig. S4, A and B), indicating that ZNRF1 does not affect the transcription of TLR3. As TLR3 interacts with its ligand in endolysosomes (Johnsen et al., 2006), we examined whether ZNRF1 affects ligand internalization, altering of TLR3 signaling. To address this issue, we tracked the uptake of rhodamine-conjugated poly(I:C). The amount of internalized poly(I:C) was similar in control and *Znrf1*^−/−^ BMDMs (Fig. 5 A), indicating that ZNRF1 does not affect poly(I:C) internalization. Accumulated results have revealed that the trafficking of nucleic acid–sensing TLRs is crucial for their activation of downstream signaling (Ewald et al., 2011; Garcia-Cattaneo et al., 2012; Toscano et al., 2013). TLR3 needs to undergo proteolytic processing within its ectodomain to be able to activate downstream signaling upon ligand binding (Garcia-Cattaneo et al., 2012). Unfortunately, no commercial TLR3 antibody was available for detecting endogenous cleaved TLR3 protein. To resolve this issue, we took advantage of Myc-HA knock-in *Tlr3* mice (herein called *Tlr3*^t/t^), in which the endogenous TLR3 C-terminal tail was fused with dual Myc-HA tags using the CRISPR/Cas9 system (Chen et al., 2021). We first assessed the effect of ZNRF1 on TLR3 proteolytic processing and found similar amounts of cleaved TLR3 proteins in control and ZNRF1 knockdown TLR3^t/t^ BMDMs, regardless of poly(I:C) treatment (Fig. 5 B). To determine whether ZNRF1 regulates TLR3 trafficking, we crossed *Znrf1*^−/−^ mice with *Tlr3*^t/t^ mice (named *Znrf1*^−/−^*Tlr3*^t/t^) and generated MEFs from these mice and their wild-type littermates. Costaining was carried out with the early endosome marker early endosomal antigen 1 (EEA1), MVBs marker, cluster of differentiation 63 (CD63), and lysobis-phosphatidic acid (LBPA), or the late endosome/lysosome marker lysosomal-associated membrane protein 2 (LAMP2) with TLR3(Myc) upon poly(I:C) stimulation. TLR3 and EEA1 colocalization was comparable in control and ZNRF1-depleted cells in the early phase after poly(I:C) stimulation (Fig. 5, C and D). However, TLR3-EEA1 colocalization was gradually decreased in wild-type cells but not in *Znrf1*^−/−^ MEFs after 120 min post-poly(I:C) treatment, suggesting that ZNRF1 is not involved in TLR3 transport from the ER to endosomes but controls its trafficking beyond the early endosome. Colocalization of TLR3 and CD63, LBPA, or LAMP2 was significantly increased in wild-type MEFs after 180 min post-poly(I:C) stimulation; however, TLR3 trafficking to CD63^+^, LBPA^+^, or LAMP2^+^ vesicles was significantly reduced in *Znrf1*^−/−^ MEFs (Fig. 5, E–J; and Fig. S4, C and D), suggesting that ZNRF1 promotes TLR3 trafficking from endosomes to MVBs/lysosomes. As endosomal TLR-containing endocytic cargos transport these to lysosomes, they become progressively acidic, eventually leading to degradation of the TLRs and termination of their downstream signaling (McAlpine et al., 2018). In line with this concept, the pH value declined gradually in wild-type BMDMs after poly(I:C) stimulation, whereas acidification of TLR3 endolysosomes was impaired in *Znrf1*^−/−^ BMDMs (Fig. 6, A and B; and Fig. S4 E). To examine whether TLR3 protein is degraded in LAMP2^+^ vesicles, we purified LAMP2^+^ vesicles using antibodies against LAMP2 followed by immunoblotting with antibodies against proteins from various subcellular compartments. Our data revealed that TLR3 proteins decreased gradually in LAMP2^+^ compartments after poly(I:C) stimulation and the lysosome inhibitor Bafilomycin A1 blocked TLR3 degradation in LAMP2^+^ compartments, indicating that activated TLR3 is degraded in lysosomes (Fig. S4, F–H). To confirm that delayed TLR3 trafficking to lysosomes leads to enhanced stability of TLR3, we assessed the poly(I:C)-induced TLR3 protein level in the presence of the protein synthesis inhibitor cycloheximide (CHX). As expected, the level of TLR3 in wild-type BMDMs declined gradually after poly(I:C) stimulation, while it remained stable in *Znrf1*^−/−^ cells (Fig. 6, C and D; and Fig. S4 I). However, ZNRF1 did not influence TLR3 expression at the steady-state (Fig. 6, E and F). ZNRF1-mediated TLR3 degradation after poly(I:C) stimulation was significantly attenuated in the presence of the lysosome inhibitor chloroquine, but the proteasome inhibitor MG132 suppressed TLR3 degradation slightly (Fig. 6 G). The endosomal sorting complexes required for transport (ESCRT) has been reported to mediate the sorting of nucleic acid sensing TLRs to MVBs for degradation (Majer et al., 2019a). In addition, TLR3 is known to associate with the hepatocyte growth factor–regulated tyrosine kinase substrate (HRS) of the ESCRT-0 complex, and this association increases after poly(I:C) stimulation (Li et al., 2020). Coimmunoprecipitation assays showed that both TLR3 and TLR9 interact with the key ESCRT-0 component, HRS (Fig. S4 J). Consistent with a previous report, poly(I:C) induced the interaction of TLR3 and HRS, but this interaction was significantly less pronounced in *Znrf1*^−/−^*Tlr3*^t/t^ BMDM (Fig. 6 H). These data together suggest that ZNRF1 promotes TLR3 sorting to MVBs/lysosomes for degradation by the ESCRT complex, resulting in the termination of its downstream signaling.

### ZNRF1-mediated TLR3 K63-linked polyubiquitination at K813 reduces type I IFN production and EMCV propagation

**Figure 7.**
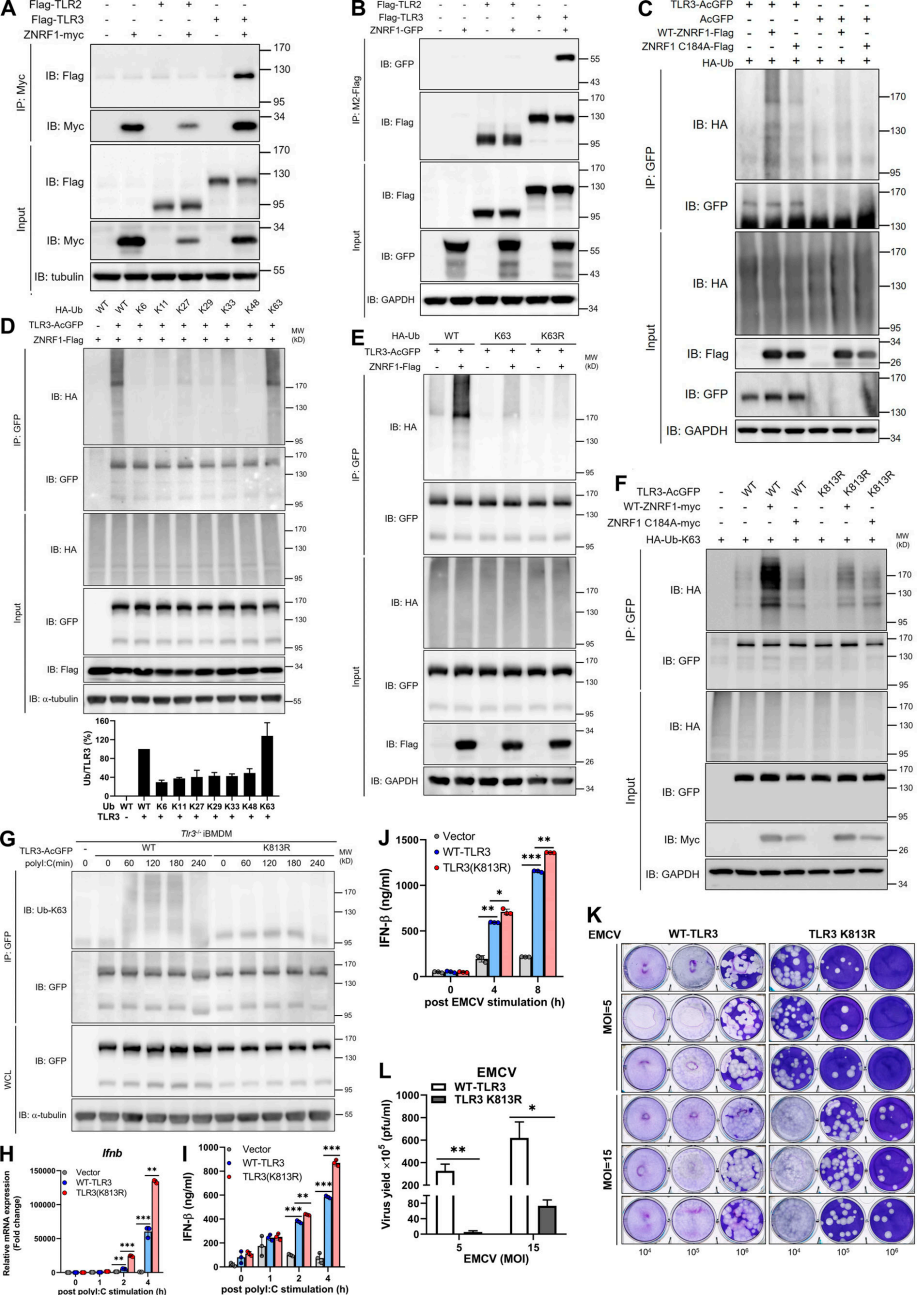
**ZNRF1 mediates TLR3 K63-linked polyubiquitination at K813 to inhibit type I IFN production and EMCV propagation. (A and B)** HEK293T were cotransfected with Flag-TLR2 or Flag-TLR3 and ZNRF1-Myc. **(C)** HEK293T were cotransfected with HA-Ub, empty vector or TLR3-AcGFP or wild-type ZNRF1-Flag or ZNRF1 (C184A)-Flag for 36 h. Cell lysates were harvested and immunoprecipitated with the antibodies indicated. Immunocomplexes, as well as WCL, were subjected to immunoblotting (IB) with the antibodies indicated. **(D)** HEK293T cells were cotransfected with GFP-tagged ZNRF1, AcGFP-tagged TLR3, and HA-tagged various ubiquitin mutants; 36 h after co-transfection, a TLR3 ubiquitination assay was carried out by immunoprecipitating TLR3 and subsequent immunoblotting with anti-HA antibody. Quantification of TLR3 ubiquitination is shown in the lower panel of D. **(E)** HEK293T cells were cotransfected with the plasmids indicated for 36 h. Cell lysates were immunoprecipitated using anti-GFP antibodies. The immunoprecipitates were analyzed by immunoblotting using the antibodies indicated. **(F)** HEK293T cells were cotransfected with the plasmids indicated HA-Ub-K63, wild-type ZNRF1-Flag, or ZNRF1 (C184A)-Flag, and the indicated AcGFP-tagged wild-type TLR3 or TLR3 mutants. After 36 h, cell lysates were immunoprecipitated with the antibodies indicated. The immunocomplexes, as well as WCL, were subjected to immunoblotting with the antibodies indicated. **(G and H)** *Tlr3*^−/−^ iBMDM were reconstituted with either AcGFP-tagged wild-type TLR3 or TLR3(K813R) mutant. **(G)** The cell lysates were immunoprecipitated with anti-GFP antibodies. The immunocomplexes, as well as WCL, were subjected to immunoblotting with the antibodies indicated. **(H)** The expression of *Ifnb* mRNAs in iBMDMs after stimulation with poly(I:C) for the times indicated was analyzed by RT-qPCR. **(I)** The level of IFN-β in culture media after stimulation with poly(I:C) for the times indicated was measured by ELISA. **(J)** The level of IFN-β in culture media after infection with EMCV at MOI of 10 for the times indicated was measured by ELISA. **(K)** Cells were infected with EMCV at the MOI indicated for 24 h; viral titers in culture media were determined by plaque assay on Vero cells. **(L)** Quantification of EMCV virus particles in K. *P < 0.05, **P < 0.01, and ***P < 0.001 (Student’s *t* test). Data are representative of three independent experiments (error bars, mean ± SD). Source data are available for this figure: SourceData F7.

K63-linked ubiquitination has been shown to direct receptor cargo into the ILVs of MVBs, resulting in receptor degradation in lysosomes (Kwon and Ciechanover, 2017; Majer et al., 2019a; Migliano and Teis, 2018; Sardana and Emr, 2021; Zhang et al., 2021; Zhu et al., 2017). We demonstrated recently that ZNRF1 controls EGFR endocytic trafficking by mediating its ubiquitination (Shen et al., 2021). We hypothesized, therefore, that ZNRF1 mediates TLR3 ubiquitination to control receptor trafficking and signaling. We determined first the interaction of TLR3 and ZNRF1 by performing reciprocal immunoprecipitation (IP) analysis, and our results clearly show that ZNRF1 interacts with TLR3, but not TLR2, in human embryonic kidney 293T (HEK293T) cells exogenously expressing these proteins (Fig. 7, A and B). In addition, ZNRF1 associated with other endosomal TLRs, TLR7 and TLR9, but not plasma TLRs, TLR4, and TLR1 (Fig. S4 K). Domain mapping experiments revealed that the RING domain of ZNRF1 is responsible for its interaction with TLR3 (Fig. S4 L). To determine whether ZNRF1 catalyzes TLR3 ubiquitination, we coexpressed wild-type ZNRF1 or the catalytic mutant ZNRF1(C184A) with AcGFP-tagged TLR3 in HEK293T cells and determined the level of TLR3 ubiquitination. ZNRF1, but not the enzymatically inactive mutant ZNRF1(C184A), induced strong polyubiquitination of TLR3 (Fig. 7 C). In line with its dispensable role in TLR2 signaling, ZNRF1 was not able to ubiquitinate TLR2 (Fig. S4 M). Surprisingly, despite its binding to TLR7, ZNRF1 was dispensable for TLR7 ubiquitination (Fig. S4 N). In addition, ZNRF1 preferentially catalyzed K63-linked polyubiquitination of TLR3 (Fig. 7 D). Consistently, ZNRF1 failed to ubiquitinate TLR3 when coexpressing the ubiquitin K63R mutant, in which lysine 63 was replaced with arginine (Fig. 7 E). ZNRF1 did not promote K48-linked polyubiquitination of TLR3 as ZNRF1-mediated TLR3 ubiquitination still occurred in cells expressing the ubiquitin K48R mutant, in which all lysines except lysine 48 were replaced with arginines (Fig. S4 O). Taken together, these results suggest that ZNRF1 promotes K63-linked polyubiquitination of TLR3, leading to receptor degradation through the lysosomal pathway.

**Figure S5.**
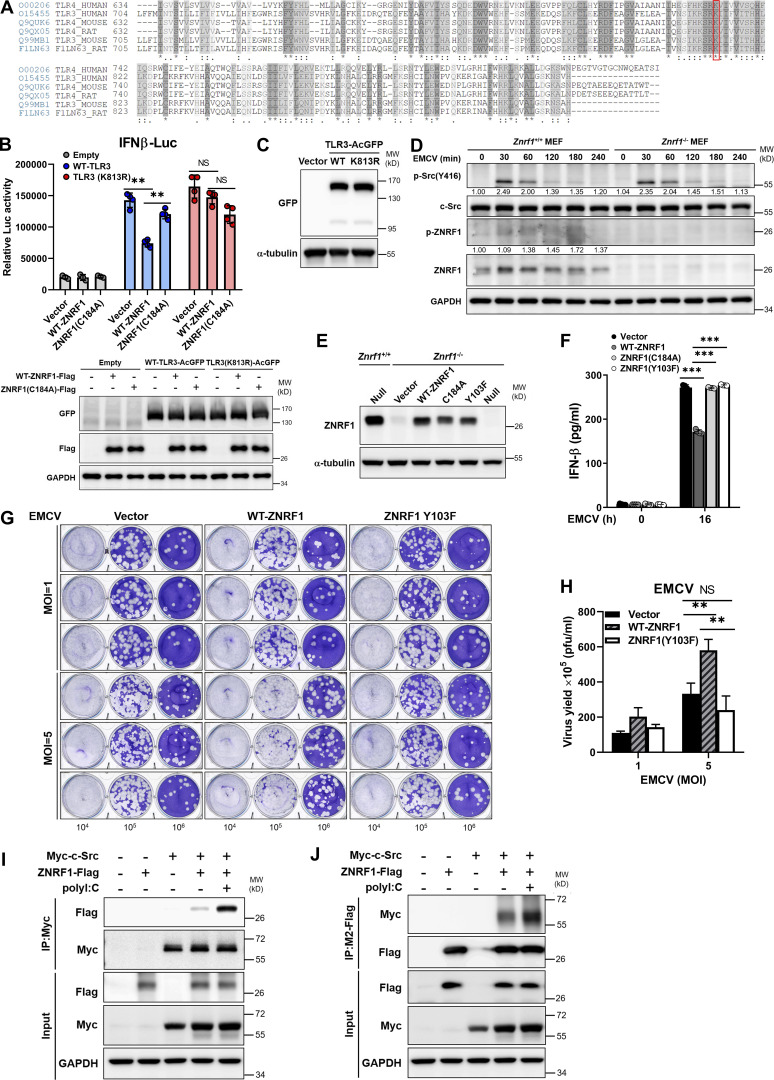
**ZNRF1 Y103 phosphorylated by c-Src is required for its regulation of TLR3-driven IFNs production and antiviral immunity. (A)** Sequence alignment of the TIR-domain region of three species (human, mouse, and rat) of TLRs (including TLR3 and TLR4) obtained from the UniProt website (https://www.uniprot.org/). An asterisk (*) denotes an identical residue, a colon (:) denotes conserved substitutions, and a period (.) denotes semiconserved substitutions. The conserved lysine residue in TLR3 and TLR4 proteins across different species is highlighted within the red box. **(B)** Wild-type TLR3 and TLR3(K813R) mutant stably expressing HEK293T cells were cotransfected with IFNβ-Luc reporter and wild-type ZNRF1 or ZNRF1(C184A) mutant. After 24 h, cells were stimulated with poly(I:C) (50 μg/ml) for 8 h, followed by the dual-luciferase reporter assay. The expression of the proteins indicated in cell lysates was confirmed by immunoblotting, as shown in the lower panel. **(C)** Immunoblot analysis of TLR3-AcGFP protein in lysates from *Tlr3*^−/−^ iBMDMs reconstituted with vector, AcGFP-tagged wild-type TLR3, or TLR3(K813R). **(D)** Immunoblotting analysis of the phosphorylation of c-Src and ZNRF1, as well as total c-Src and ZNRF1, in lysates of *Znrf1*^+/+^ and *Znrf1*^−/−^ MEFs infected with EMCV (MOI of 1) for the times indicated. The intensities of the bands are expressed as fold increases compared to those of untreated control cells, after normalization to their unphosphorylated forms. **(E)** *Znrf1*^−/−^ RAW264.7 cells were reconstituted with Tet-inducible vector, wild-type ZNRF1 or ZNRF1(C184A) or ZNRF1(Y103F) mutant. The protein expression of ZNRF1 in cell lysates was analyzed by immunoblotting. **(F)** *Znrf1*^−/−^ RAW264.7 cells reconstituted with vector, wild-type ZNRF1, and ZNRF1(Y103F) mutant were infected with EMCV at an MOI of 10 for 16 h. The IFN-β levels in the culture media were measured by RT-qPCR. **(G and H)** *Znrf1*^−/−^ RAW264.7 cells reconstituted with vector, wild-type ZNRF1, and ZNRF1(Y103F) mutant were infected with EMCV at the MOI indicated for 24 h. **(G)** Viral titers in the supernatants were determined by plaque assays. **(H)** Quantification of viral titers in G. **(I and J)** HEK293T cells were cotransfected with Myc-tagged c-Src and ZNRF1-Flag for 36 h followed by stimulation with poly(I:C) (50 μg/ml). Cell lysates were immunoprecipitated with anti-M2-Flag or Myc antibodies. The immunoprecipitates and WCL were analyzed by immunoblotting with the antibodies indicated. **P < 0.01 and ***P < 0.001 (Student’s *t* test). Data are representative of two independent experiments (error bars, mean ± SD). Source data are available for this figure: SourceData FS5.

Our previous results showed increased IRF3 phosphorylation in LPS-induced *Znrf1*^−/−^ macrophages, implying a similar mechanism controlling endosomal TLR4–TRIF signaling by ZNRF1 (Lee et al., 2017). We hypothesized that ZNRF1 mediates ubiquitination of endosomal TLR3 and TLR4 to control their downstream signaling. We aligned the TIR domains of human, mouse, and rat TLR3 and TLR4 and found that the lysine 813 (K813) of mouse TLR3 is conserved (Fig. S5 A). To determine whether K813 of TLR3 is the acceptor of ubiquitination mediated by ZNRF1, we substituted K813 with arginine (K813R). ZNRF1 failed to mediate K63-linked polyubiquitination on TLR3(K813R) when overexpressed in HEK293T cells (Fig. 7 F). Reporter assays showed that TLR3(K813R) was resistant to the ZNRF1-mediated suppressive effect on activation of the IFN-β promoter (Fig. S5 B). To determine whether ZNRF1-mediated TLR3 ubiquitination is critical for antiviral responses, we reconstituted *Tlr3*^−/−^ iBMDMs with wild-type TLR3 or TLR3(K813R) mutant (Fig. S5 C). Similar to the results in HEK293T cells, the reconstitution of *Tlr3*^−/−^ iBMDM with TLR3(K813R) displayed significantly reduced TLR3 ubiquitination in response to poly(I:C) (Fig. 7 G). Poly(I:C)- or EMCV-induced IFN-β mRNA and cytokine release were significantly increased in *Tlr3*^−/−^ iBMDMs reconstituted with TLR3(K813R) compared with that in cells reconstituted with wild-type TLR3 (Fig. 7, H–J). Accordingly, viral titers were reduced in the culture medium of cells reconstituted with TLR3(K813R) after EMCV challenge (Fig. 7, K and L). Taken together, ZNRF1 mediates K63-linked polyubiquitination on TLR3 K813 to control TLR3-mediated antiviral immunity.

### ZNRF1-mediated polyubiquitination of TLR3 requires activation by c-Src via phosphorylation of Y103

**Figure 8.**
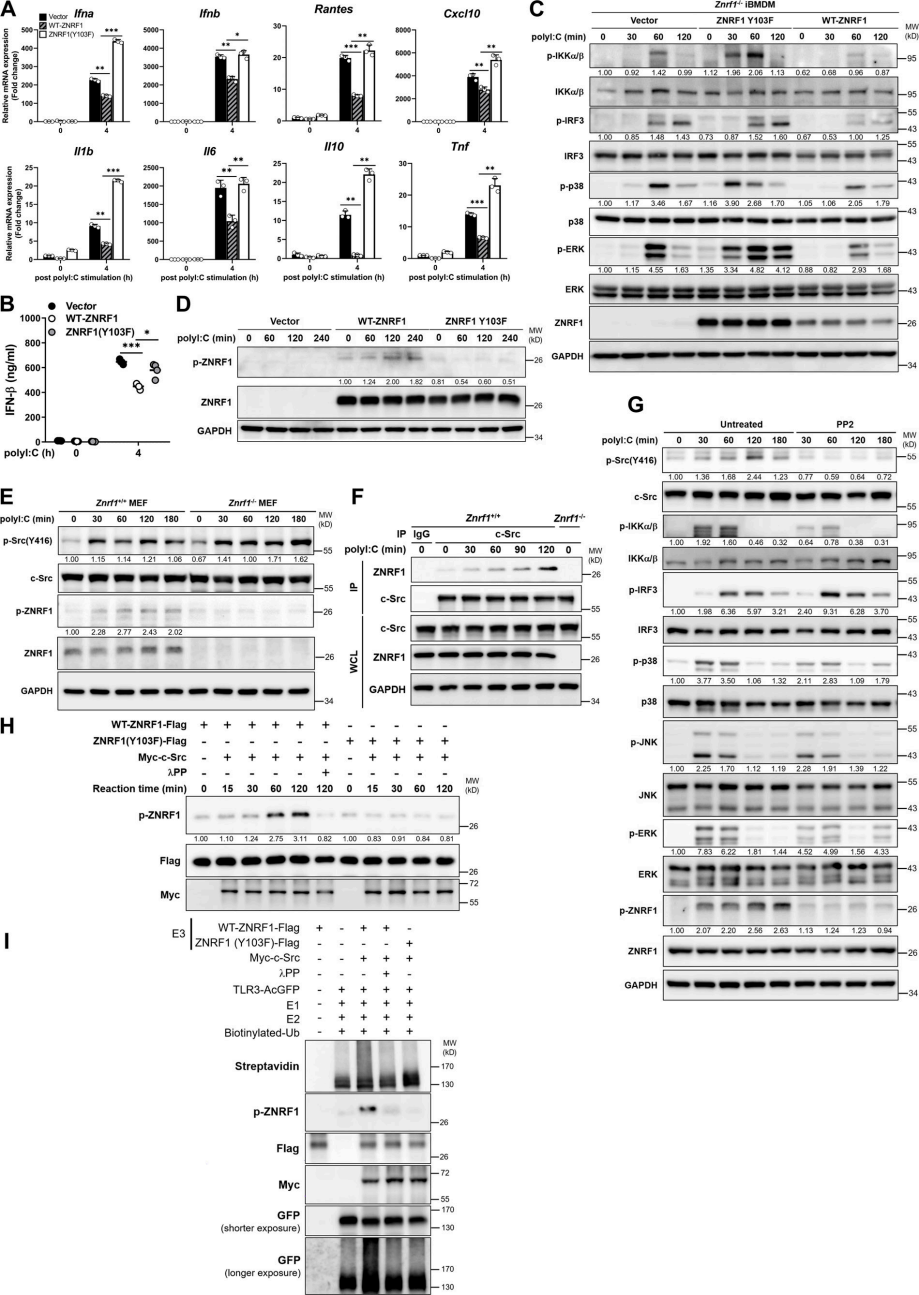
**ZNRF1-mediated TLR3 polyubiquitination requires activation by c-Src via phosphorylation at its Y103. (A–C)** *Znrf1*^−/−^ iBMDMs were reconstituted with Tet-inducible vector, wild-type ZNRF1, or ZNRF1(Y103F) mutant, and stimulated with poly(I:C) (30 μg/ml) for the times indicated. **(A)** The expression of the mRNAs indicated was analyzed by RT-qPCR. **(B)** The secreted IFN-β in culture media after stimulation with poly(I:C) for 4 h was measured by ELISA analysis. **(C)** Cell lysates were subjected to immunoblotting with the antibodies indicated. **(D)** *Znrf1*^−/−^ RAW264.7 reconstituted with vector, wild-type ZNRF1, or ZNRF1(Y103F) mutant were stimulated with poly(I:C) (30 μg/ml) for the times indicated. Cell lysates were analyzed by immunoblotting. **(E)** *Znrf1*^+/+^ and *Znrf1*^−/−^ MEFs were stimulated with poly(I:C) (100 μg/ml) for the times indicated and cell lysates were analyzed by immunoblotting. The intensities of the bands are expressed as fold increases compared with those of untreated control cells after normalization to their unphosphorylated forms. **(F)** *Znrf1*^+/+^ or *Znrf1*^−/−^ BMDMs were stimulated with poly(I:C) (30 μg/ml) for the times indicated. Cell lysates were immunoprecipitated with either IgG or anti–c-Src antibody, and the immunocomplexes and WCL were subjected to immunoblotting with the antibodies indicated. **(G)** BMDMs from *Znrf1*^+/+^ mice were either pretreated with or without PP2 (10 μM) for 1 h, followed by stimulation with poly(I:C) (30 μg/ml) in the absence of PP2 for the times indicated. Cell lysates were collected and analyzed by immunoblotting with the antibodies indicated. **(H)** HEK293T was transfected with Flag-tagged wild-type ZNRF1, ZNRF1(Y103F), or Myc-tagged c-Src for 24 h. Cell lysates were immunoprecipitated with anti-M2-Flag or anti-Myc antibodies. In vitro kinase assays were performed with immunoprecipitated Flag-tagged wild-type ZNRF1 or ZNRF1(Y103F) and Myc-tagged c-Src with or without λPP as indicated at 30°C for the reaction times indicated, followed by immunoblotting with the antibodies indicated. The intensities of the bands are expressed as fold increases compared with those of untreated control cells, after normalization to their unphosphorylated forms. **(I)** In vitro ubiquitination assays were carried out with Flag-tagged wild-type ZNRF1 or ZNRF1(Y103F) incubated with Myc–c-Src prepared from H and AcGFP-tagged TLR3 immunopurified from HEK293T cells transfected with AcGFP-TLR3 and recombinant ubiquitin catalytic components as indicated at 37°C for 3 h. The mixtures were then subjected to immunoblotting with the antibodies indicated. *P < 0.05, **P < 0.01, and ***P < 0.001 (Student’s *t* test). Data are representative of three independent experiments (error bars, mean ± SD). Source data are available for this figure: SourceData F8.

The ubiquitin ligase activity of ZNRF1 has been reported to be activated by EGFR-mediated phosphorylation of its tyrosine 103 residue under oxidative stress (Wakatsuki et al., 2015). We hypothesized that TLR3 engagement induces ZNRF1 activation, probably through phosphorylation. To assess whether tyrosine 103 phosphorylation is critical for the TLR3 immune response, we reintroduced wild-type and ZNRF1(Y103F) mutant, whose tyrosine 103 was replaced with phenylalanine, into *Znrf1*^−/−^ iBMDMs and challenged with poly(I:C). While reconstitution with wild-type ZNRF1 decreased poly(I:C)-triggered expression of cytokine mRNAs, IFN-β production, and activation of IKK, MAPKs, and IRF3, reconstitution with vector or ZNRF1(Y103F) did not (Fig. 8, A–C), indicating that tyrosine 103 of ZNRF1 is critical for its suppressive effect on the TLR3 immune response. To determine whether ZNRF1 is phosphorylated at Y103 upon TLR3 activation, we generated a specific antibody against ZNRF1 phosphorylated at Tyr97 and Tyr103. The specificity of anti-ZNRF1 phosphorylation was confirmed in ZNRF1-sufficient and -deficient cells (Fig. 8, D and E). As expected, poly(I:C) and EMCV triggered ZNRF1 phosphorylation (Fig. 8, D and E; and Fig. S5 D). However, ZNRF1(Y103F) failed to be phosphorylated after poly(I:C) stimulation (Fig. 8 D), indicating that TLR3 ligation induces ZNRF1 phosphorylation at Y103, which in turn regulates TLR3 signaling. We confirmed the critical role of ZNRF1 Y103 phosphorylation in the TLR3-mediated antiviral response to EMCV infection. Reconstitution of *Znrf1*-deficient cells with wild-type ZNRF1, but not ZNRF1(Y103F) mutant, decreased EMCV-induced type I IFN expression (Fig. S5, E and F) and increased virion production (Fig. S5, G and H). Since EGFR expression is negligible in primary macrophages, macrophage cell lines, and MEFs, EGFR is unlikely to be the tyrosine kinase that phosphorylates ZNRF1 upon TLR3 activation. To determine which tyrosine kinase phosphorylates ZNRF1, we used the public NetPhos program (NetPhos 3.1 Server) to identify tyrosine kinases that may phosphorylate ZNRF1 Y103. The best three candidate kinases were EGFR, insulin receptor, and the non-receptor kinase c-Src. It has been reported that c-Src is involved in TLR3-mediated antiviral signaling (Johnsen et al., 2006). We speculated that c-Src phosphorylates and activates ZNRF1 after TLR3 activation. In line with the previous result, c-Src was activated in MEFs after challenge with poly(I:C) and EMCV, and ZNRF1 deletion had no impact on its activation (Fig. 8 E and Fig. S5 D). To investigate whether c-Src phosphorylates ZNRF1 to modulate TLR3-elicited signaling, we examined first the association of c-Src with ZNRF1 by coimmunoprecipitation analysis. Our results show that c-Src interacted with ZNRF1, and this interaction was further enhanced after poly(I:C) challenge (Fig. 8 F and Fig. S5, I and J). To confirm that ZNRF1 is phosphorylated by c-Src, we pretreated cells with PP2, a Src inhibitor, for 1 h and stimulated with poly(I:C) in the absence of PP2. ZNRF1 phosphorylation was hampered, whereas IRF3 activation was increased in poly(I:C)-stimulated BMDMs pretreated with PP2 (Fig. 8 G). To assess further whether c-Src directly phosphorylates ZNRF1 at Y103, we carried out an in vitro kinase assay using immunopurified ZNRF1 and c-Src from HEK293T cells overexpressing Flag-tagged ZNRF1 and Myc-tagged c-Src, respectively. Indeed, c-Src was able to phosphorylate wild-type ZNRF1, but not ZNRF1(Y103F), and ZNRF1 phosphorylation was abolished in the presence of lambda protein phosphatase (λPP; Fig. 8 H), indicating that c-Src specifically phosphorylates ZNRF1 at tyrosine 103. To determine whether c-Src-mediated phosphorylation of ZNRF1 directly catalyzes TLR3 ubiquitination, we performed an in vitro ubiquitination assay using phosphorylated ZNRF1 in an in vitro kinase assay with immunopurified TLR3 from HEK293T-expressing GFP-tagged TLR3 and other essential ubiquitination components. Our results show that c-Src–dependent ZNRF1 phosphorylation is required for TLR3 ubiquitination, and TLR3 ubiquitination is suppressed when ZNRF1 phosphorylation is eliminated by λPP (Fig. 8 I). Collectively, these results suggest that ZNRF1 is phosphorylated at Y103 and activated by c-Src, which subsequently promotes TLR3 polyubiquitination.

### ZNRF1 protects mice from *Staphylococcus aureus* superinfections enabled by antiviral immunity

**Figure 9.**
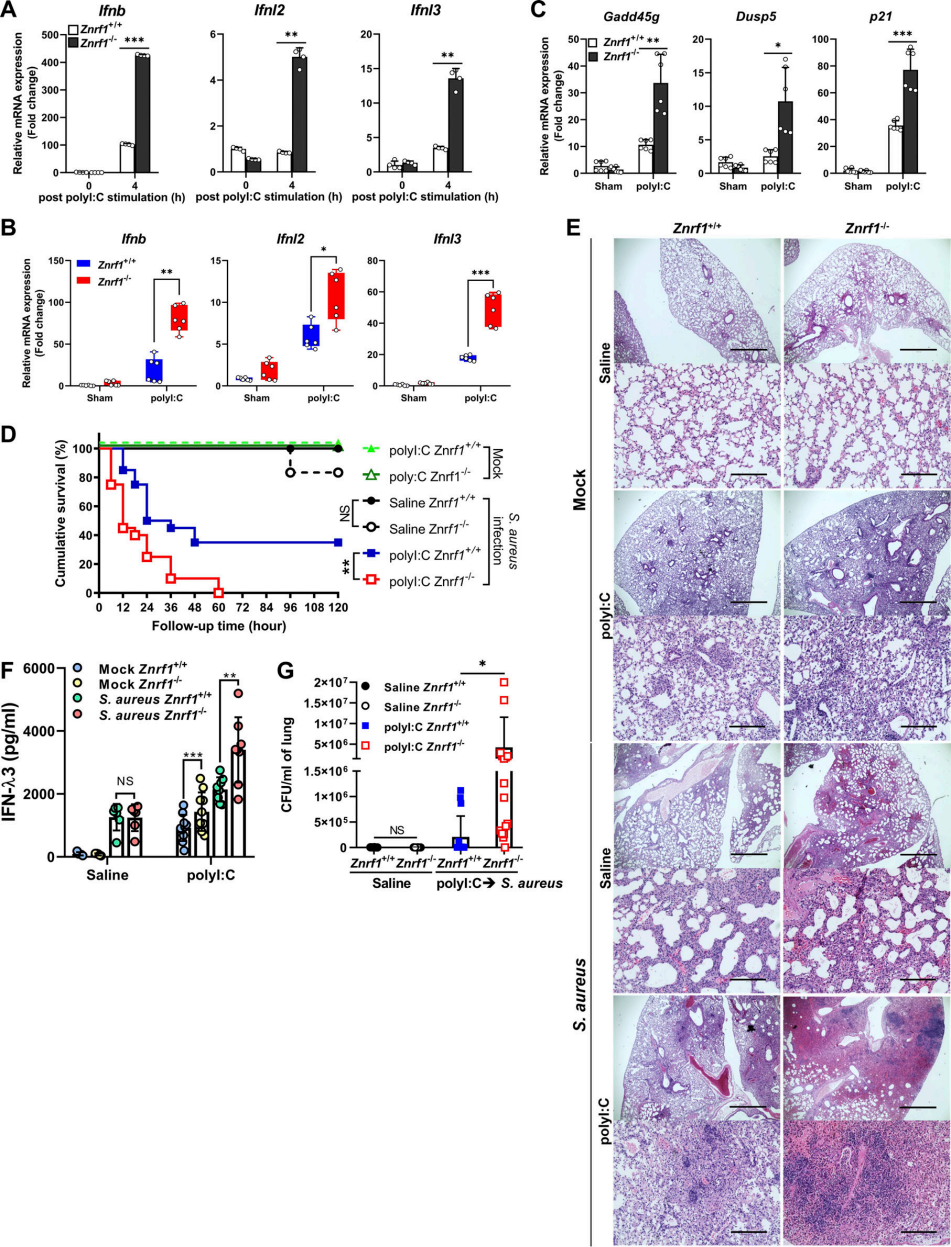
**ZNRF1 protects mice from *S. aureus* superinfection induced by antiviral immunity. (A)** RT-qPCR analysis of the expression of *Ifnb*, *Ifnl2*, and *Ifnl3* in BMDMs from *Znrf1*^+/+^ and *Znrf1*^−/−^ mice after poly(I:C) (30 μg/ml) stimulation for 4 h. **(B–F)** *Znrf1*^+/+^ and *Znrf1*^−/−^ mice were administered poly(I:C) (2.5 mg/kg) or saline i.t. daily for 6 d. **(B and C)** RT-qPCR analysis of the mRNA expression of type I and III IFNs and p53-dependent antiproliferative genes in lung tissue from *Znrf1*^+/+^ and *Znrf1*^−/−^ mice. **(D)** *Znrf1*^+/+^ and *Znrf1*^−/−^ mice administered poly(I:C) (2.5 mg/kg) i.t. for 6 d were infected i.t. with 4 × 10^7^ CFU of *S. aureus* and monitored for survival. **(E–G)** Mice administered poly(I:C) (2.5 mg/kg) i.t. for 6 d were i.t. infected with 5 × 10^7^ CFU of *S. aureus*. Mice were sacrificed 18 h after infection. **(E)** H&E staining of histological sections of lung tissues. Objective magnification, ×4 and ×20. Scale bars, 1,000 and 200 μm, respectively. **(F)** IFN-λ3 protein levels from lung homogenates were evaluated by ELISA. **(G)** Lung bacterial burdens normalized by lung homogenates were determined by colony-forming assay. *P < 0.05, **P < 0.01, and ***P < 0.001 (Student’s *t* test). Data are representative of three independent experiments (error bars, mean ± SD). Log-rank (Mantel-Cox) test, corrected for multiple comparisons, was performed to monitor survival in D.

Mounting evidence suggests that infections by specific viruses lead to increased susceptibility to opportunistic bacterial infections, called superinfections (Grajales-Reyes and Colonna, 2020; Rossi et al., 2020). Recent studies have shown that excessive or prolonged type I and III IFN responses to viral infections impaired lung epithelial regeneration by upregulated p53 signaling, resulting in bacterial superinfection (Broggi et al., 2020; Major et al., 2020). As with upregulated type I IFNs production, we found that ZNRF1 deficiency also enhanced the mRNA expression of type III (*Ifnl2*, *Ifnl3*) IFNs in BMDMs in response to poly(I:C) (Fig. 9 A). We hypothesized that ZNRF1 controls TLR3 trafficking and termination of TLR3 signaling to prevent excess type I and type III IFNs production and facilitate lung repair, which prevents opportunistic bacterial infections. To test this hypothesis, we set up an animal model published in a previous study (Broggi et al., 2020) by treating mice with an intratracheal administration of poly(I:C) for 6 d. As expected, poly(I:C) strongly upregulated the mRNA expression of type I and III IFNs, as well as p53-regulated genes, including *Gadd45g*, *Dusp5*, and *p21*, in the lungs of *Znrf1*^−/−^ mice (Fig. 9, B and C). We asked further whether ZNRF1 is crucial for the pathogenesis of *S. aureus* superinfections enabled by antiviral responses by infecting mice treated with poly(I:C) with *S. aureus*. In line with the results in the previous report, about 60% of the mice that received poly(I:C) died after *S. aureus* infection. However, ZNRF1-deficient mice treated with poly(I:C) had increased mortality and more IFN-λ3 production, higher bacterial burdens, and increased lung inflammation upon *S. aureus* infection (Fig. 9, D–G). Collectively, these data suggest that ZNRF1 regulates TLR3-triggered type I and III IFNs production, thereby protecting mice against *S. aureus* superinfection induced by antiviral immunity.

## Discussion

**Figure 10.**
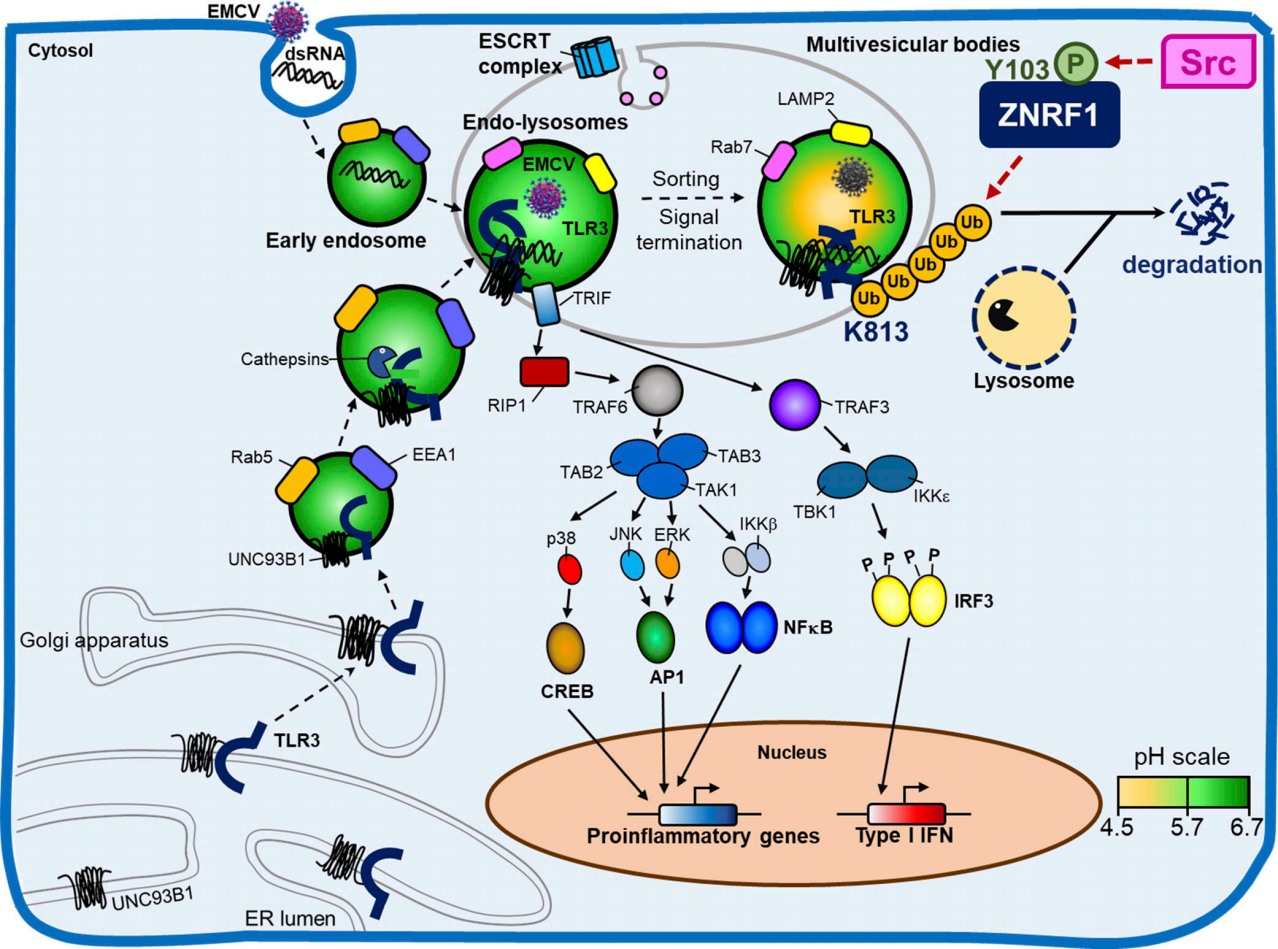
**Graphical model summarizing regulation of TLR3 trafficking and type I IFN production by ZNRF1.** Upon TLR3 activation by poly(I:C) or an invading RNA virus, ZNRF1 is activated by c-Src kinase by phosphorylation at tyrosine 103. ZNRF1 then catalyzes K63-linked polyubiquitination of TLR3 at lysine 813, which subsequently promotes TLR3 trafficking from endolysosomes to MVBs/lysosomes for degradation, thereby terminating TLR3-triggered innate immune responses. When ZNRF1 is depleted, TLR3 ubiquitination is decreased, thereby increasing endosomal accumulation of TLR3, which leads to prolonged activation of its downstream signaling and enhanced production of type I IFNs and inflammatory mediators.

Type I IFNs are crucial for the innate immune response to and adaptive immunity against viral infection, but dysregulation of type I IFNs production is linked to numerous autoimmune and infectious diseases and the recently identified inflammatory cytopenias (McNab et al., 2015; Mesev et al., 2019). Activation of endosomal TLR3, TLR7, TLR8, or TLR9 by recognition of viral nucleic acids induces the robust production of type I IFNs to inhibit viral propagation; however, the termination of endosomal TLR–mediated signaling and type I IFNs production remains poorly understood. In this study, we demonstrate that the RING finger E3 ubiquitin ligase, ZNRF1, is activated by TLR3-induced c-Src kinase and, in turn, mediates K63-linked polyubiquitination of TLR3, leading to TLR3 trafficking to MVBs/lysosomes for degradation and termination of its downstream signaling (Fig. 10). ZNRF1 deficiency results in prolonged TLR3 signaling and type I IFNs production, thereby enhancing antiviral immunity against EMCV and SARS-CoV-2 infection, but rendering mice more susceptible to opportunistic bacterial infection due to increased lung tissue damage. Our findings indicate that ZNRF1 is a pivotal brake controlling TLR3 endocytic trafficking and the termination of signaling to avoid the adverse effects of excess type I/III IFNs production.

TLR3-driven type I IFN production is indispensable for defense against EMCV and HSV-1, as shown by human and mouse studies (Hardarson et al., 2007; Lafaille et al., 2012; Prehaud et al., 2005; Zhang et al., 2007). Recent studies demonstrated that mutations of TLR3 and TLR7 and some of the downstream signaling molecules required for type I IFNs production are correlated with the severity of COVID-19, indicating the critical roles of TLR3 and TLR7 signaling in the control of SARS-CoV-2 (Asano et al., 2021; Laurent et al., 2022; van der Sluis et al., 2022; Zhang et al., 2020). It was confirmed that SARS-CoV-2 is sensitive to type I IFNs treatment in vitro and in an in vivo hamster infection model (Paludan and Mogensen, 2022). Nevertheless, type I IFNs are detrimental to the host in the late phase of infection by respiratory viruses, including SARS-CoV-2 and influenza virus, due to enhanced inflammation and tissue damage (Broggi et al., 2020; Major et al., 2020; McNab et al., 2015). Therefore, type I IFNs production needs to be spatiotemporally regulated to avoid adverse effects on the host. Our current studies show that ZNRF1 ubiquitinated TLR3 and controlled receptor trafficking to lysosomes for degradation, thereby terminating its downstream signaling and type I IFNs production. Thus, although ZNRF deficiency renders cells resistant to EMCV and SARS-CoV-2 and mice to EMCV, mice with ZNRF1 deficiency are susceptible to bacterial infection due to increased lung tissue damage. These results suggest that ZNRF1 functions as a critical brake in TLR3 signaling and type I IFN production during the antiviral response to prevent excessive inflammation and bacterial superinfection. Notably, TLR7 is scarcely expressed in lung epithelial cells (Travaglini et al., 2020) and human TLR7 mainly functions in plasmacytoid dendritic cells to protect the host cells against SARS-CoV-2 infection (Asano et al., 2021; Laurent et al., 2022; van der Sluis et al., 2022). Thus, the protective phenotype observed in *ZNRF1*^−/−^ Calu-3 cells was probably mainly through dysregulation of TLR3 signaling. Nevertheless, it will be worthwhile to explore whether ZNRF1 controls type I IFN immunity against SARS-CoV-2 through modulation of TLR7 signaling under in vivo condition.

Endosomal TLR trafficking is known to be critical for the control of the downstream signaling pathways and the transcriptional activation of distinct sets of genes (Miyake et al., 2018), despite that their trafficking is differently regulated (Lee et al., 2013; Saitoh et al., 2017). With the assistance of UNC93B1, endosomal TLRs are required to be transported to the endolysosomal compartments, where receptors are proteolytically cleaved to become signaling-competent and encounter their bona fide ligands (Qi et al., 2012). Studies in human cells and mice indicate that the downstream signaling of TLR7 and TLR9, which lead to the induction of inflammatory cytokines and type I IFNs, is initiated in distinct endosomal compartments, named NF-κB and IRF7 endosomes, respectively, and the adaptor protein AP-3 controls the sequential activation from NF-κB to IRF7 after ligand binding (Gotoh et al., 2010; Sasai et al., 2010). In addition, TLR7 cargo needs to be ubiquitinated and sorted into MVBs/lysosomes to terminate TLR7 signaling to avoid overproduction of type I IFNs (Majer et al., 2019a). These data together suggest that trafficking of endosomal TLR7, and probably TLR9, is sequential from endolysosomes, MVB/ILVs, and eventually, to lysosomes for degradation to restrain TLRs signaling. AP-3 also is known to be required for TLR3 trafficking to endosomal compartments for type I IFN production (Sasai et al., 2010), but the detailed mechanisms of its trafficking remain largely unclear. Our studies show that ZNRF1 mediated K63-linked ubiquitination of TLR3 at K813 and promoted TLR3 cargo into MVBs/lysosomes for degradation to terminate signaling. However, although ZNRF1 also negatively modulates the TLR7-mediated immune response, ZNRF1 failed to catalyze TLR7 ubiquitination when overexpressed in HEK293T cells, indicating that ZNRF1 might control the termination of TLR3 and TLR7 signaling through different mechanisms.

K63-linked ubiquitination often labels cargo for sorting into MVBs and then into lysosomes (Duncan et al., 2006; Geetha and Wooten, 2008). The ESCRT machinery, which is required for sorting ubiquitinated cargos into MVBs/lysosomes for degradation, consists of four complexes, ESCRT-0, -I, -II, and -III (Raiborg and Stenmark, 2009). The ESCRT-0, -I, and -II complexes contain subunits, including HRS and tumor susceptibility 101, which can directly associate with ubiquitylated cargo through their ubiquitin-interacting motifs. HRS have been reported to be required for ubiquitinated TLR3, TLR7, and TLR9 transport to endolysosomes and induction of signaling (Chiang et al., 2012; Li et al., 2020). Our results show that ZNRF1 does not affect the protein stability of TLR3 during the steady-state, probably due to lack of phosphorylation at its Y103 by c-Src. In addition, the levels of the TLR3 cleaved form and colocalization of TLR3 and EEA1 in the early phase after poly(I:C) stimulation were not influenced in ZNRF1-depleted cells, indicating that ZNRF1 is dispensable for TLR3 targeting to endolysosomes. Interestingly, TLR3 has been reported to be conjugated with K63-linked ubiquitin chains at K831 by the E3 ubiquitin ligase TRIM3, in response to poly(I:C), which is essential for TLR3 targeting to endolysosomes for receptor proteolytic processing (Li et al., 2020). We demonstrated that ZNRF1 mediates K63-linked ubiquitination of TLR3 at K813, and mutation of this lysine residue with arginine led to impaired TLR3 sorting into MVBs/lysosomes without affecting its transport to endolysosomes. Similar to TLR7 and TLR9 (Ewald et al., 2008), our results show that most of endogenous TLR3 in unstimulated cells are the cleaved form. Therefore, it is possible that full-length TLR3 is ubiquitinated by TRIM3 and then transported by the ESCRT complex to the endolysosomal compartment for proteolytic processing. The cleaved functional TLR3 may need to be deubiquitinated in endolysosomes to stably localize to this compartment before encountering its ligand. Upon ligand binding in endolysosomes, TLR3 is ubiquitinated by ZNRF1 at K813, which facilitates TLR3 shuttling to MVBs/lysosomes for degradation and attenuation of signaling. However, we cannot rule out the possibility that an increased extent of TLR3 ubiquitination, in combination with TRIM3 and ZNRF1, enhances the efficiency of TLR3 lysosomal sorting and degradation, as for EGFR (Shen et al., 2021). K63-linked ubiquitination of UNC93B1 has been reported to be essential for sorting TLR7, but not TLR3 or TLR9, cargo into MVBs/lysosomes and the termination of TLR7 signaling (Majer et al., 2019a), although the E3 ubiquitin ligase responsible remains unknown. It remains to be determined which E3 ubiquitin ligase regulates TLR7 and TLR9 signaling by controlling their trafficking.

As endocytic cargos traffic to MVBs/lysosomes for degradation, the pH of compartments is progressively decreased to enhance the activity of lysosomal hydrolases. We observed impaired acidification of poly(I:C)-containing cargos in ZNRF1-depleted cells, which confirms the function of ZNRF1 in controlling TLR3 sorting to MVBs/lysosomes. Previously, it was reported that ZNRF2, the closely related E3 ubiquitin ligase of ZNRF1, interacts with the V-ATPase to maintain functional lysosomes (Hoxhaj et al., 2016). It remains to be determined whether ZNRF1 also promotes the acidification of TLR3 cargos by controlling lysosomal function, as does ZNRF2, thereby promoting TLR3 degradation and attenuating signaling.

Previous studies showed that ZNRF1 is phosphorylated at Y103 and activated by oxidative stress-induced EGFR during neuronal/axonal degeneration (Wakatsuki et al., 2015). However, the expression of EGFR was not detected in macrophages and MEFs, indicating that EGFR is not involved in the activation of ZNRF1, following TLR3 activation. c-Src kinase has been shown to be activated and participates in the initiation of TLR3 signaling (Johnsen et al., 2006). Our results reveal the critical function of c-Src in ZNRF1 after TLR3 activation, indicating that c-Src is not only essential for TLR3-driven signaling but also involved in the negative feedback loop via ZNRF1 to terminate TLR3 signaling by promoting receptor lysosomal sorting and degradation. The TLR3-mediated immune response has been shown to be crucial for host defense against certain viruses, including SARS-CoV-2, HSV-1, and EMCV (Hardarson et al., 2007; Prehaud et al., 2005; Zhang et al., 2020). However, TLR3 activation must be tightly regulated to prevent harmful effects on the host. Despite numerous studies revealing the regulation of the immune response mediated by TLR3, the mechanism underlying the termination of TLR3 signaling remains obscure. Our results indicate that TLR3 activation induces ZNRF1 activity, which in turn mediates K63-linked polyubiquitination of TLR3, resulting in ubiquitinated TLR3 sorting into lysosomes for degradation and termination of TLR3 signaling. Our findings are consistent with the notion that the sorting and lysosomal degradation of activated receptors are critical mechanisms for terminating receptor-mediated signaling (Weeratunga et al., 2020). Type I IFNs are known to be detrimental to the host in the late phase of infections by SARS-CoV-2 and influenza virus and are the major cause of morbidity in viral pneumonia (Broggi et al., 2020; Major et al., 2020; McNab et al., 2015). In our studies, ZNRF1-deficient mice were vulnerable to *S. aureus* superinfections triggered by poly(I:C), indicating the important physiological function of ZNRF1 in the spatiotemporal regulation of TLR3-mediated innate immune responses to prevent the pathogenesis of diseases caused by excessive production of IFNs and inflammatory cytokines. The clinical outcomes in individuals infected by SARS-CoV-2 range from no symptoms to severe or lethal COVID-19, and single-gene inborn errors of the TLR3 pathway have been found to be risk factors for this disease (Zhang et al., 2020). It may be worthwhile examining whether inborn errors in the *ZNRF1* gene can be identified in COVID-19 patients and are associated with disease severity.

## Materials and methods

### Mice

*Znrf1*^F/F^ mice were generated as previously described (Lee et al., 2017). To generate mice with systemic deletion of *Znrf1*, *Znrf1*^F/F^ mice were crossed with Protamine-Cre mice (O’Gorman et al., 1997). A male *Znrf1*^F/+^:Protamine-Cre mouse was mated with a *Znrf1*^F/F^ female mouse to obtain *Znrf1*^F/−^ offspring, which were then backcrossed with C57BL/6 mice to obtain *Znrf1*^+/−^ mice. Mice expressing C-terminal Myc-HA tagged TLR3 (hereafter called *Tlr3*^t/t^) were generated previously (Chen et al., 2021). These mice were maintained in a specific pathogen–free animal facility. All animal experiments were conducted following the animal welfare guidelines and were approved by the Institutional Animal Care and Use Committee of College of Medicine, National Taiwan University (approval no. 20190062).

### Cell culture and BMDMs preparation

MEFs, HEK293T cells, and African green monkey kidney Vero cells were cultured in DMEM (Gibco) containing 10% (vol/vol) heat-inactivated FBS and 100 U/ml penicillin/streptomycin at 37°C in humidified 5% CO_2_. Human non-small-cell lung cancer cell line Calu-3 cells and murine macrophage-like RAW264.7 cells grown in RPMI 1640 (Gibco) supplemented with 10% FBS. BMDMs were prepared as described previously (Lee et al., 2017). Briefly, femurs and tibia bones were collected from 6- to 8-wk-old mice and the bone marrow was flushed out with DMEM medium using a 25-gauge syringe. The bone marrow cells were collected and cultured in high-glucose DMEM medium supplemented with 20% L929 cell–conditioned medium for 7 d to differentiate into macrophages. BMDMs were collected and cultured in DMEM containing M-CSF (10 ng/ml) for further experiments.

### Generation of immortalized macrophage progenitors

Retroviral transduction of immortalized macrophage progenitors was conducted as described previously (Redecke et al., 2013). In brief, the retroviral plasmid MSCV-ERHBD-Hoxb8 (kindly provided by Dr. Hans Hacker, the University of Utah, Salt Lake City, UT, USA) was cotransfected with the packaging plasmids pCL-Eco into HEK293T cells. Retroviral particles were collected from the supernatants 48 h after transfection. Bone marrow cells were isolated and resuspended at a concentration of 5 × 10^5^ cells/ml in RPMI 1640 supplemented with 15% FBS containing recombinant mouse IL-3 (10 ng/ml), IL-6 (20 ng/ml), and stem cell factor (250 ng/ml), followed by 2 d culture. Cells were collected, resuspended in progenitor outgrowth medium (RPMI 1640 medium supplemented with 10% FBS, 1 μM β-estradiol; Sigma-Aldrich), and 10 ng/ml GM-CSF, and transduced with retrovirus by centrifugation at 1,500 *g* for 60 min in the presence of 8 μg/ml hexadimethrine bromide (Polybrene; Sigma-Aldrich). After retroviral infection for 2 d, the cells were cultured with 3 ml of fresh progenitor outgrowth medium. The medium was replaced every 3 d until immortalized macrophage progenitors were expanding stably. To prepare macrophages from iBMDMs, cells were washed twice with sterilized PBS to remove β-estradiol followed by 7 d culture in DMEM medium supplemented with 30% L929 cell-conditioned medium, as described above.

### Generation of *ZNRF1*^*−/−*^ Calu-3 cells using the CRISPR/Cas9 system

HEK293T cells were cotransfected with the lentiviral packaging plasmids pMD.G and pCMVR8.91, with the CRISPR/single guide RNA (sgRNA)/puro expression plasmid expressing a sgRNA sequence targeting the exon 1 of human *ZNRF1*. After 48 h, the culture medium containing lentiviruses was collected and used to infect Calu-3 cells for 24 h, followed by puromycin selection. The sgRNA target sequences are 5′-GAT​TTC​GGG​CAC​TAC​CGG​AC-3′ for sgRNA #1 and 5′-GCA​TTT​CGG​GCA​CTA​CCG​GA-3′ for sgRNA #2. To verify gene editing in single-cell clones, genomic DNA was purified and subjected to PCR and sequencing. The primers used for PCR are: forward primer 5′-TTG​ACT​CCC​TCC​CCC​TTT​ATG​CTC​G-3′ and reverse primer 5′-ATA​GGT​GGA​GTC​GGA​CGC​AGA​CCC​T-3′ for clones from sgRNA #1, and forward primer 5′-TTG​ACT​CCC​TCC​CCC​TTT​ATG​CTC​G-3′ and reverse primer 5′-ATA​GGT​GGA​GTC​GGA​CGC​AGA​CCC​T-3′ for clones from sgRNA #2.

### EMCV propagation and plaque assay

EMCV was kindly provided by Dr. Lih-Hwa Hwang (National Yang-Ming University, Hsinchu, Taiwan). For EMCV amplification, Vero cells in DMEM medium supplemented with 2% (vol/vol) FBS, 1 mM sodium pyruvate, and 100 U/ml penicillin/streptomycin were infected with EMCV at a multiplicity of infection (MOI) of 0.01 for 2–3 d. Culture media were collected and centrifuged at 1,500 *g* for 10 min at 4°C. The supernatant containing viruses was collected and stored at −80°C until use. For EMCV plaque assays, culture medium from EMCV-infected cells was serially diluted and used to infect 90% confluent Vero cells cultured in 6-well plates for 2 h. After infection, the cells were gently washed and overlaid with 1% low melting agarose (SeePlaque, catalog no. 50111; Lonza) containing DMEM supplemented with 2% FBS. After incubation for 2 d, the overlays were removed, and the cells were fixed with 10% formaldehyde at room temperature for 30 min, followed by staining with 1% crystal violet in 20% methanol. Plaques were counted, averaged, and multiplied by the dilution fold to calculate the viral titer, which is expressed as plaque-forming units per ml (pfu/ml).

### SARS-CoV-2 amplification and infection

SARS-CoV-2 virus was propagated in VeroE6 cells in DMEM supplemented with 2 μg/ml tosylsulfonyl phenylalanyl chloromethyl ketone-trypsin (T1426; Sigma-Aldrich), and the virus titer was determined by the plaque assay as described previously (Cheng et al., 2020). The virus isolate used in the current study is SARS-CoV-2/NTU03/TWN/human/2020 (Global Initiative on Sharing All Influenza Data accession ID EPI_ISL_413592). The Calu-3 cells were seeded into 24-well culture plates at 3 × 10^5^ cells/well in DMEM with 10% FBS and penicillin G sodium 100 U/ml, streptomycin sulfate 100 μg/ml, and amphotericin B 250 ng/ml (antibiotic-antimycotic;15240-062; Gibco) 1 d before infection. The cells were washed once with PBS before incubation with SARS-CoV-2 at the MOI indicated for 1 h at 37°C. The virus inoculum was removed and the cells were washed again with PBS before supplementation with fresh DMEM containing 2% of FBS for 24 and 48 h at 37°C. Finally, the culture supernatant was harvested for the plaque assay and real-time quantitative PCR (RT-qPCR) to determine the titers of infectious viruses and the viral RNA, individually (Cheng et al., 2020).

### RNA purification and RT-qPCR

Total cellular RNA was extracted using NucleoZOL reagent (#MN-740404.200; MACHEREY-NAGEL) following the company’s instructions. 1 μg total RNA was used to synthesize cDNA using the RevertAid H Minus First Strand cDNA Synthesis Kit (Thermo Fisher Scientific) according to the manufacturer’s instructions. The amount of specific cDNA was determined by RT-qPCR using Maxima SYBR Green/Fluorescein qPCR Master Mix (#4367659; Thermo Fisher Scientific) following the manufacturer’s instructions. All RT-qPCR values of interesting genes were normalized to cyclophilin A or GAPDH transcript as an internal control. All data are presented as fold-change relative to the unstimulated sample. The primer sequences are listed in Table S1.

### RNA-sequencing analysis

Total RNAs were prepared from BMDMs and further purified by the TruSeq Stranded mRNA Library Prep Kit (Illumina) following the manufacturer’s recommendations. Briefly, 1 μg total RNA was subjected to oligo(dT) magnetic beads, fragmented, and reverse transcribed to synthesize the first strand cDNA using random primers. After the generation of double-strand cDNA and adenylation on the 3′ ends of the DNA fragments, the adaptors were ligated and purified with the AMPure XP system (Beckman Coulter). The quality of the libraries was assessed on the Agilent Bioanalyzer 2100 system and a Real-Time PCR system. The qualified libraries were then sequenced on an Illumina NovaSeq 6000 platform with 150 bp paired-end reads generated by Genomics, BioSci & Tech Co. The bases with low quality and sequences from adapters in raw data were removed using the program Trimmomatic (version 0.39; Bolger et al., 2014). The filtered reads were aligned to the reference genomes using Bowtie2 (version 2.3.4.1; Langmead and Salzberg, 2012). A user-friendly software RSEM (version 1.2.28) was used for quantification of the transcript abundance (Li and Dewey, 2011). Differentially expressed genes were identified by EBSeq (version 1.16.0), followed by the functional enrichment analyses of GO terms and Kyoto Encyclopedia of Genes and Genomes pathways, implemented in an R package, clusterProfiler (version 3.6.0; Ashburner et al., 2000; Kanehisa and Goto, 2000; Yu et al., 2012). To identify the differential gene expression in response to poly(I:C), fold-change (>2×) and *t* tests (P value <0.05) of *Znrf1*^−/−^ BMDMs at 4 h after poly(I:C) stimulation were compared with that of *Znrf1*^+/+^ BMDMs. The GO terms used in this study include “response to type I IFN” (GO:0034340), “inflammatory response” (GO:0006954), and “cytokine production involved in inflammatory response” (GO:0002534).

### Lentiviral production and virus transduction

HEK293T cells were cotransfected with pLVX-AcGFP-N1 constructs and the packaging plasmids pMD.G and pCMVR8.91 using Turbofect transfection (#MBIR0531; Thermo Fisher Scientific) according to the manufacturer’s recommendations. The supernatants containing the lentivirus were harvested 48 and 72 h after transfection. Immortalized macrophage progenitors, HEK293T, or MEFs were transduced with lentiviruses in the presence of 8 μg/ml polybrene by spin inoculation at 1,500 *g* for 60 min and cultured in fresh medium for another 24 h. The infected cells were then selected in 2.5–5 μg/ml puromycin (#P600-100; Gold Biotechnology) containing medium until the uninfected cells were completely eliminated. The stable colonies were pooled for further experiments.

### Dual-luciferase reporter and ELISAs

HEK293T cells were cotransfected with the firefly luciferase reporter plasmid indicated (pIFNβ-Luc or pNFκB-Luc), pRL-TK-*Renilla* luciferase plasmid, and the plasmids indicated, and cultured for 48 h. Cells were lysed and firefly and *Renilla* luciferase activities were determined using the dual-luciferase reporter assay system (Promega) according to the manufacturer’s instructions. Firefly luciferase activity was normalized relative to that of *Renilla* luciferase. The levels of cytokines and IFN-β in sera and culture supernatants were determined using the ELISA systems (R&D Systems or #SEA222Mu; Cloud-clone corp) according to the manufacturer’s recommendations.

### Immunoblotting and IP

Cells were lysed in ice-cold lysis buffer containing 50 mM Tris-HCl, pH 7.5, 150 mM NaCl, 2 mM EDTA, 1% Triton X-100, 0.5% NP-40, 10% Glycerol, 20 mM sodium fluoride (NaF), 2 mM dithiothreitol, 1 mM PMSF, 2 mM p-nitrophenyl phosphate, 1 mM sodium orthovanadate (Na_3_VO_4_), and protease inhibitors including 2 μg/ml Aprotinin, 1 μg/ml Benzamidine, 1 μg/ml Pepstatin A, and 2 μg/ml Leupeptin (Sigma-Aldrich) for 30 min and homogenized by sonication (Branson Ultrasonics Sonifier 250; Thermo Fisher Scientific) with three 30 s bursts, separated by 1-min intervals, and incubation on ice for 30 min. After centrifugation at 12,000 *g* for 30 min, cellular extracts were collected and protein concentrations were determined using the Bio-Rad Protein Assay (Bio-Rad). For IP of ubiquitin-modified proteins, 20 mM *N*-ethylmaleimide (Sigma-Aldrich) was added to the lysis buffer before cell lysis. Cellular extracts (250–500 μg) were incubated with anti-FLAG or c-Myc antibody-conjugated agarose beads (Sigma-Aldrich) at 4°C for 3 h or the indicated primary antibody overnight at 4°C followed by a 2-h incubation with Protein G Agarose (#16-266; Millipore). The immunocomplexes were pelleted by centrifugation, washed three times with lysis buffer, and resuspended in SDS-PAGE sample-loading buffer (50 mM Tris-HCl, pH 6.8, 10% glycerol, 2% SDS, 20 mM β-mercaptoethanol, and 0.1% bromophenol blue). The immunocomplexes were then separated by SDS-PAGE and transferred to polyvinylidene fluoride membranes (Millipore). The membranes were blocked with 10% skim milk in TBST (50 mM Tris-HCl, pH 7.6, 150 mM NaCl, 0.05% Tween-20) or Blocking One (Nacalai Trsque) for 1 h at room temperature and then incubated with the indicated primary antibody overnight at 4°C, followed by an HRP-conjugated secondary antibody (Jackson ImmunoResearch) for 1 h at room temperature. Immunoreactive signals were detected using Luminata Western Chemiluminescent HRP substrates (Millipore) according to the manufacturer's instructions.

### Semidenaturing detergent agarose gel electrophoresis

Cells were harvested and resuspended in the sample buffer (0.5× Tris borate EDTA containing 0.065 M Tris, pH 7.6, 22.5 mM boric acid, 1.25 mM EDTA, 10% glycerol, 2% SDS, and 0.0025% bromophenol blue) and loaded onto a horizontal 1.5% agarose gel containing 0.1% SDS. After electrophoresis in the running buffer (1 × Tris acetate EDTA containing 40 mM Tris, 20 mM acetic acid, 1 mM EDTA, and 0.1% SDS) for 30 min with a constant voltage of 100 V at 4°C, the proteins were transferred to polyvinylidene fluoride membrane for 16 h followed by immunoblotting.

### LAMP2^+^ vesicle isolation

Cells were harvested in 1 ml of prechilled homogenization buffer containing 10 mM HEPES-KOH, pH 7.4, 220 mM mannitol, 70 mM sucrose, and protease inhibitors. Cells were homogenized with 40 strokes in a 1-ml tissue homogenizer with a loose pestle. The unbroken cells and cellular debris were removed by centrifugation at 1,000 *g* for 5 min at 4°C. The mitochondria-enriched pellets were pelleted by centrifugation at 10,000 *g* for 10 min at 4°C. The supernatants were used to purify LAMP2^+^ vesicles by incubation with anti-LAMP2 antibody overnight at 4°C, followed by a 2 h incubation with Protein G agarose beads. The beads were washed three times with protease inhibitor–containing homogenization buffer and resuspended in SDS-PAGE sample-loading buffer for immunoblotting.

### Immunofluorescence assay

Cells were seeded on coverslips and cultured overnight before treatment. Cells were then fixed with 4% paraformaldehyde (Electron Microscopy Sciences) in PBS (Gibco), pH 7.4, at room temperature for 30 min and permeabilized with 0.25% Triton X-100 in PBS at room temperature for 10 min, followed by blocking with 1% BSA in PBST (0.25% Triton X-100 in PBS) at 25°C for 30 min. The coverslips were then incubated with primary antibody overnight at 4°C, washed with PBS, and stained with a fluorescent-conjugated secondary antibody (Jackson ImmunoResearch) at 25°C for 1 h. After extensive washing with PBS, the coverslips were mounted with DAPI Fluoromount-G (#0100-20; SouthernBiotech) to counterstain cell nuclei. Images were captured using a Zeiss LSM 700 Confocal microscope (Zeiss) with a 60× objective. The colocalization of TLR3 with different organelle markers (EEA1, CD63, LBPA and LAMP2) was analyzed using the open-source Fiji (ImageJ) software.

### Poly(I:C) internalization assay

For the poly(I:C) internalization assay, cells were placed on ice to stop internalization, incubated with 200 ng/ml poly(I:C) (HMW) conjugated Rhodamine (InvivoGen) for 1 h at 4°C, and subsequently transferred to 37°C for the times indicated. After three washes with ice-cold PBS, the cells were subjected to an acid wash (0.2 M acetic and 0.5 M NaCl, pH 2.8) for 5 min at 4°C. The cells were then detached from the culture dishes, washed with PBS, and resuspended in PBS containing 2% FBS and 0.01% sodium azide, followed by fixation with 4% paraformaldehyde in PBS for 30 min. Fixed cells were analyzed by a BD LSR II flow cytometer (BD Biosciences).

### In vitro kinase and ubiquitination assays

HEK293T cells were transfected with Flag-tagged wild-type or ZNRF1 (Y103F) or Myc-tagged c-Src plasmids for 24 h. Total cell lysates were prepared by lysing cells in IP lysis buffer (20 mM Tris-HCl, pH 7.5, 100 mM NaCl, 1% Triton X-100, and protease inhibitors) at 4°C for 30 min followed by centrifugation at 12,000 *g* for 30 min. Cell lysates were immunoprecipitated using anti-M2-Flag or anti-Myc agarose beads and incubated at 4°C for 3 h. The beads were washed with IP lysis buffer three times.

For in vitro kinase assay, the immunoprecipitated Flag-tagged wild-type or ZNRF1 (Y103F) were coincubated with the immunoprecipitated Myc-tagged c-Src extractions in the kinase buffer (100 μM ATP, 20 mM Tris-HCl, pH 7.4, 1 mM EGTA, 5 mM MgCl_2_, 0.02% β-mercaptoethanol, and 0.2 mg/ml BSA) with or without λPP (#P0753S; New England Biolabs) at 30°C for the reaction times indicated, followed by immunoblotting analysis. For in vitro ubiquitination assays, the immunoprecipitated beads from in vitro kinase assays were collected by centrifugation and washed twice. The beads were then incubated with 2.5 μM biotinylated ubiquitin, 100 nM E1 (UBA1), and 2.5 μM E2 (UbcH5c) purchased from Enzo Life Science, and AcGFP tagged TLR3 immunoprecipitated from HEK293T cells ectopically expressing AcGFP-TLR3 in the ubiquitination buffer (Enzo Life Science) containing 5 mM MgCl_2_ and 5 mM ATP for 3 h at 37°C. The reactions were terminated by adding an equal volume of 2× non-reducing gel loading buffer (Enzo Life Science) and analyzed by immunoblotting.

### Animal models of EMCV infection and *S. aureus* superinfection

For EMCV infection, sex- and age-matched *Znrf1*^+/+^ and *Znrf1*^−/−^ mice (6–8 wk old) were infected i.p. with EMCV 10^4^ (for survival study) or 10^7^ pfu in 100 μl of DPBS per mouse. The mice were either monitored for survival every day or sacrificed at 72 h after infection to collect blood and tissues. *S. aureus* (Newman strain) was kindly provided by Dr. Yung-Chi Chang (Department and Graduate Institute of Medical Microbiology, National Taiwan University, Taipei, Taiwan). *S. aureus* grown in Brain Heart Infusion (#90003-040; BD Biosciences) agar plate at 37°C overnight was subcultured and grown to an OD_600_ of 0.4, centrifuged, and resuspended in DPBS immediately before infection. For *S. aureus* superinfection, 2.5 mg/kg of poly(I:C) HMW or saline was intratracheally (i.t.) administered daily for 6 d. Each mouse was infected i.t. with 4–5 × 10^7^ CFU of *S. aureus* and sacrificed 18 h after infection, except in the survival assay. Blood and tissues were collected for cytokine and histological analyses.

### Statistical analysis

GraphPad Prism8.0 software was used for data analysis. Results are presented as the mean ± SEM. Statistical significance was determined by unpaired, two-tailed Student’s *t* test for two-group comparisons, one-way ANOVA with Dunnett’s multiple comparisons test for comparisons of more than two groups, two-way ANOVA for comparisons of more than two groups with two or more timepoints, or the log-rank test for survival experiments. P values <0.05 were considered statistically significant.

### Online supplemental material

Fig. S1 shows that ZNRF1 is induced by endosomal TLR activation and is not involved in TLR2-driven inflammatory responses (related to Fig. 1). Fig. S2 shows that ZNRF1 does not participate in RLR-mediated antiviral signaling or type I IFN–triggered immune responses (related to Fig. 1). Fig. S3 shows that ZNRF1 deficiency in MEFs and BMDMs enhances type I IFN production and restricts EMCV propagation (related to Fig. 3). Fig. S4 shows that ZNRF1 does not affect TLR3 mRNA expression or mediate K48-linked polyubiquitin chains on TLR3, and ZNRF1 associates with TLR7 (related to Figs. 5 and 7). Fig. S5 shows that ZNRF1 Y103 phosphorylated by c-Src is required for its regulation of TLR3-driven type I IFNs production and antiviral immunity (related to Fig. 8). Table S1 lists primer pairs used for RT-qPCR analysis. Table S2 lists primer pairs used for genotyping. Table S3 provides a complete list of experimental materials, including antibodies, chemicals, peptides, recombinant proteins, critical commercial assay kits, pathogens, cell lines, mouse strain, plasmids and software, and algorithms for this study.

## Supplementary Material

Table S1 lists primer pairs used for RT-qPCR.

Table S2 lists primer pairs of genotyping.

Table S3 provides a complete list of experimental materials, including antibodies, chemicals, peptides, recombinant proteins, critical commercial assay kits, pathogens, cell lines, mouse strain, plasmids and software, and algorithms for this study.

SourceData F1 is the source file for Fig. 1.

SourceData F3 is the source file for Fig. 3.

SourceData F4 is the source file for Fig. 4.

SourceData F5 is the source file for Fig. 5.

SourceData F6 is the source file for Fig. 6.

SourceData F7 is the source file for Fig. 7.

SourceData F8 is the source file for Fig. 8.

SourceData FS1 is the source file for Fig. S1.

SourceData FS2 is the source file for Fig. S2.

SourceData FS3 is the source file for Fig. S3.

SourceData FS4 is the source file for Fig. S4.

SourceData FS5 is the source file for Fig. S5.

## Data Availability

All RNA-seq data were deposited in the GEO database under accession number GSE226295. The data and materials that support the findings of this study are available from the corresponding author upon reasonable request.
