# Supplementary material for: The Src–ZNRF1 axis controls TLR3 trafficking and interferon responses to limit lung barrier damage
Source: J Exp Med. 2023 May 9;220(8):e20220727. doi: 10.1084/jem.20220727 (PMC10174191; doi:10.1084/jem.20220727)
Supplement: Table S1 — lists primer pairs used for RT-qPCR. [file JEM_20220727_TableS1.docx]

**Supplementary Table 1. Primer pairs RT-qPCR**

| Species | Gene | Strand | Sequence |
| --- | --- | --- | --- |
| Mouse | *Ifna4* | Forward | 5’-CCTGTGTGATGCAGGAACC-3’ |
|  |  | Reverse | 5’-TCACCTCCCAGGCACAGA-3’ |
| Mouse | *Ifnb* | Forward | 5’-GAAGAGTTACACTGCCTTTGCC-3’ |
|  |  | Reverse | 5’-CAACAATAGTCTCATTCCACCC-3’ |
| Mouse | *Rantes* | Forward | 5’-GACACCACTCCCTGCTGCTTTG-3’ |
|  |  | Reverse | 5’-GATGTATTCTTGAACCCACTTCTT-3’ |
| Mouse | *Il1b* | Forward | 5’-GAACTCAACTGTGAAATGCCACC-3’ |
|  |  | Reverse | 5’-CCACAGCCACAATGAGTGATACT-3’ |
| Mouse | *Il6* | Forward | 5’-ACAAGAAAGACAAAGCCAGAGTC-3’ |
|  |  | Reverse | 5’-ATTGGAAATTGGGGTAGGAAG-3’ |
| Mouse | *Il10* | Forward | 5’-TGGGTTGCCAAGCCTTATCGG-3’ |
|  |  | Reverse | 5’-ACCTGCTCCACTGCCTTGCTC-3’ |
| Mouse | *Il12b* | Forward | 5’-TGCAGATGAAGCCTTTGAAGA-3’ |
|  |  | Reverse | 5’-AACGCACCTTTCTGGTTACAC-3’ |
| Mouse | *Tnf* | Forward | 5’-GTCTACTGAACTTCGGGGTGATC-3’ |
|  |  | Reverse | 5’-TCCACTTGGTGGTTTGCTACG-3’ |
| Mouse | *Cxcl10* | Forward | 5’-CCAAGTGCTGCCGTCATTTTCT-3’ |
|  |  | Reverse | 5’-TTCCCTATGGCCCTCATTCTCA-3’ |
| Mouse | *Tlr3* | Forward | 5’-TTGTCTTCTGCACGAACCTG-3’ |
|  |  | Reverse | 5’-CCCGTTCCCAACTTTGTAGA-3’ |
| Mouse | *Cyclophilin A* | Forward | 5’-ATGTGCCAGGGTGGTGACTTT-3’ |
|  |  | Reverse | 5’-TTGCCATCCAGCCATTCAGTC-3’ |
| Mouse | *Ifit3* | Forward | 5’-CTGAAGGGGAGCGATTGATT-3’ |
|  |  | Reverse | 5’-AACGGCACATGACCAAAGAGTAGA-3’ |
| Mouse | *Mx1* | Forward | 5’-TGCTGTACTGCTAAGTCCAAA-3’ |
|  |  | Reverse | 5’-GCAGTAGACAATCTGTTCCATCTG-3’ |
| Mouse | *Oas2* | Forward | 5’-CTATGATGCACTAGGTCAACTGC-3’ |
|  |  | Reverse | 5’-TTCCTTTCATACTGTTTGTACCAGT-3’ |
| Mouse | *Ifnl2* | Forward | 5’- CCTGAAGGTCTGGGAGAACATG-3’ |
|  |  | Reverse | 5’- AAGAGGCTGGCCCAGGAT-3’ |
| Mouse | *Ifnl3* | Forward | 5’- GGCCCAGAGCAAGGAGACT-3’ |
|  |  | Reverse | 5’- TTGAAACAGGTTGGAGGTGACA-3’ |
| Mouse | *Gadd45g* | Forward | 5’- CGTCTACGAGTCCGCCAAA-3’ |
|  |  | Reverse | 5’- GCACGCAAAAGGTCACATTG-3’ |
| Mouse | *Dusp5* | Forward | 5’- GCATCCAAGTGCGAGTTCCT-3’ |
|  |  | Reverse | 5’- GTCCGGCGGGAAACATTC-3’ |
| Mouse | *p21* | Forward | 5’- AGTGTGCCGTTGTCTCTTCG-3’ |
|  |  | Reverse | 5’- ACACCAGAGTGCAAGACAGC-3’ |
| Human | *IFNB* | Forward | 5’-TGCTCTCCTGTTGTGCTTCTCCAC-3’ |
|  |  | Reverse | 5’-ATAGATGGTCAATGCGGCGTCC-3’ |
| Human | *IFNL1* | Forward | 5’-AACTGGGAAGGGCTGCCACATT-3’ |
|  |  | Reverse | 5’-GGAAGACAGGAGAGCTGCAACT-3’ |
| Human | *RANTES* | Forward | 5’-GCATCTGCCTCCCCATATTC-3’ |
|  |  | Reverse | 5’-CAGTGGGCGGGCAATG-3’ |
| Human | *GAPDH* | Forward | 5’-TGCACCACCAACTGCTTAGC-3’ |
|  |  | Reverse | 5’-GGCATGGACTGTGGTCATGAG-3’ |
| Virus | EMCV 2A2B | Forward | 5’-AATGCCCACTACGCTGGT-3’ |
|  |  | Reverse | 5’-GTCGTTCGGCAGTAGGGT-3’ |
| Virus | SARS-CoV-2 N1 | Forward | 5’- GACCCCAAAATCAGCGAAAT-3’ |
|  |  | Reverse | 5’- TCTGGTTACTGCCAGTTGAATCTG-3’ |
