# Supplementary material for: The Src–ZNRF1 axis controls TLR3 trafficking and interferon responses to limit lung barrier damage
Source: J Exp Med. 2023 May 9;220(8):e20220727. doi: 10.1084/jem.20220727 (PMC10174191; doi:10.1084/jem.20220727)
Supplement: Table S2 — lists primer pairs of genotyping. [file JEM_20220727_TableS2.docx]

**Supplementary Table 2. Primer pairs of genotyping**

| Gene | Strand | Sequence |
| --- | --- | --- |
| *Znrf1* | Forward 1 | 5’- AACATCTCAGGAAGCCACTAAC -3’ |
|  | Forward 2 | 5’- TGACTAGTCGTCGTCCCCCTTTTT-3’ |
|  | Reverse | 5’- GAAAAGGAAAACTAAAACATCG-3’ |
| *Tlr3* | Forward | 5’-CACTCTGTTTGCGAAGAG-3’ |
|  | Reverse 1 | 5’-CTATCCCTTTACCGACTC-3’ |
|  | Reverse 2 | 5’- GCGTAATCTGGAACATCG-3’ |
