# Supplementary material for: The Src–ZNRF1 axis controls TLR3 trafficking and interferon responses to limit lung barrier damage
Source: J Exp Med. 2023 May 9;220(8):e20220727. doi: 10.1084/jem.20220727 (PMC10174191; doi:10.1084/jem.20220727)
Supplement: Table S3 — provides a complete list of experimental materials, including antibodies, chemicals, peptides, recombinant proteins, critical commercial assay kits, pathogens, cell lines, mouse strain, plasmids and software, and algorithms for this study. [file JEM_20220727_TableS3.docx]

**Supplementary Table 3. Supplemental material**

| **REAGENT or RESOURCE** | **SOURCE** | **IDENTIFIER** |
| --- | --- | --- |
| **Antibodies** | | |
| phospho-IKKα/β Ser176/180 | Cell Signaling Technology | Cat # 2697; RRID: AB_2079382 |
| phospho-IRF3 Ser396 | Cell Signaling Technology | Cat # 4947; RRID: AB_823547 |
| IRF3 | Cell Signaling Technology | Cat # 4302; RRID: AB_1904036 |
| phospho-p38 Thr180/Tyr182 | Cell Signaling Technology | Cat # 4511; RRID: AB_2139682 |
| p38 | Cell Signaling Technology | Cat # 9212; RRID: AB_330713 |
| phospho-SAPK/JNK Thr183/Tyr185 | Cell Signaling Technology | Cat # 9251; RRID: AB_331659 |
| JNK/SAPK | Cell Signaling Technology | Cat # 9252; RRID: AB_2250373 |
| Phospho-p44/42 MAPK (Erk1/2) (Thr202/Tyr204) | Cell Signaling Technology | Cat# 4370; RRID: AB_2315112 |
| p44/42 MAPK (Erk1/2) | Cell Signaling Technology | Cat # 9102; RRID: AB_330744 |
| phospho-Src Tyr416 | Cell Signaling Technology | Cat # 2101; RRID: AB_331697 |
| Src | Cell Signaling Technology | Cat # 2109; RRID: AB_2106059 |
| phospho-TBK1 (Ser172) | Cell Signaling Technology | Cat # 5483; RRID: AB_10693472 |
| TBK1/NAK (D1B4) | Cell Signaling Technology | Cat # 3504; RRID: AB_2255663 |
| Myc-Tag | Cell Signaling Technology | Cat # 2278; RRID: AB_490778 |
| HRS | Cell Signaling Technology | Cat # 15087; RRID: AB_2798700 |
| GAPDH | GeneTex | Cat # GTX627408; RRID: AB_11174761 |
| CD63 | Santa Cruz Biotechnology | Cat # sc-5275; RRID:AB_627877 |
| IKKα/β | Santa Cruz Biotechnology | Cat # sc-7607; RRID: AB_675667 |
| Ubiquitin (P4D1) | Santa Cruz Biotechnology | Cat # sc-8017; RRID: AB_628423 |
| GFP | Santa Cruz Biotechnology | Cat # sc-9996; RRID: AB_627695 |
| Syntenin 1 | Santa Cruz Biotechnology | Cat # sc-515538 |
| α-Tubulin | Sigma-Aldrich | Cat # T9026; RRID: AB_477593 |
| anti-FLAG M2 affinity gel | Sigma-Aldrich | Cat # A2220; RRID: AB_10063035 |
| Anti-c-Myc Agarose Affinity Gel | Sigma-Aldrich | Cat # A7470; RRID: AB_10109522 |
| Streptavidin protein purification beads | Croyez Bioscience | Cat # C07007 |
| Streptavidin | Croyez Bioscience | Cat # C08009 |
| HA-tag (3F10) | Roche | Cat # 11867431001; RRID: AB_390919 |
| LAMP2 | Abcam | Cat # ab13524; RRID: AB_2134736 |
| EEA1 | BD Biosciences | Cat # 610456; RRID: AB_397829 |
| ZNRF1 | (Lee et al., 2017) | N/A |
| phospho-ZNRF1 (Tyr97/Tyr103) | This paper | N/A |
| Calnexin | Cell Signaling Technology | Cat # 2679; RRID: AB_2228381 |
| GM130 | Cell Signaling Technology | Cat # 12480; RRID: AB_2797933 |
| S6K | Cell Signaling Technology | Cat # 9202; RRID: AB_331676 |
| LAMP1 | Santa Cruz Biotechnology | Cat # sc-19992 |
| Cathepsin D(H-75) | Santa Cruz Biotechnology | Cat # sc-10725; RRID: AB_2292414 |
| LBPA (clone 6C4) | Millipore | Cat # MABT837 |
| **Chemicals, Peptides, and Recombinant Proteins** | | |
| High-molecular-weight (HMW) polyinosinic-polycytidylic acid (poly(I:C)) | GE Healthcare | Cat # 27-4732-01 |
| protein A-conjugated Sepharose | GE Healthcare | Cat # 17-0963-03 |
| Cycloheximide | Sigma-Aldrich | Cat # C1988 |
| Chloroquine | Sigma-Aldrich | Cat # C6628 |
| PP2 | Sigma-Aldrich | Cat # P0042 |
| Macrophage colony-stimulating factor (M-CSF) | Peprotech | Cat # 315-02 |
| granulocyte-macrophage colony-stimulating factor (GM-CSF) | Peprotech | Cat # PEP315-03-100 |
| Murine IL-3 | Peprotech | Cat # 213-13 |
| Murine IL-6 | Peprotech | Cat # 216-16 |
| Murine SCF | Peprotech | Cat # 250-03 |
| CpG ODN1826 | InvivoGen | Cat # tlrl-1826 |
| 5’- triphosphate double stranded RNA (5’ppp-dsRNA) | InvivoGen | Cat # tlrl-3prna |
| Poly(I:C) (HMW) Rhodamine | InvivoGen | Cat # tlrl-picr |
| Recombinant Mouse IFN-β | R&D systems | Cat # 8234-MB |
| pHrodo Green AM Intracellular pH Indicator | Thermo Fisher Scientific | Cat # P35373 |
| DMEM, high Glucose | GIBCO | Cat # 11965092 |
| RPMI medium 1640 with L-glutamine | GIBCO | Cat # 11875-119 |
| Methionine-free medium | GIBCO | Cat # 21013 |
| Opti-MEM | GIBCO | Cat # 32985070 |
| L-glutamine 200mM | Thermo Fisher Scientiﬁc | Cat # 25030-164 |
| Penicillin-Streptomycin 10,000U/ml | GIBCO | Cat # 15140122 |
| amphotericin B (Antibiotic-Antimycotic) | GIBCO | Cat # 15240-062 |
| tosylsulfonyl phenylalanyl chloromethyl ketone (TPCK)-trypsin | Sigma-Aldrich | Cat # T1426 |
| β-estradiol | Sigma-Aldrich | Cat # E-2758 |
| Hexadimethrine bromide (Polybrene) | Sigma-Aldrich | Cat # H9268 |
| SeaPlaque GTG Agarose | Lonza | Cat # 50111 |
| NucleoZOL reagent | MACHEREY-NAGEL | Cat # MN-740404.200 |
| Turbofect transfection reagent | Thermo Scientiﬁc | Cat # MBIR0531 |
| puromycin | Gold Biotechnology | Cat # P600-100 |
| λPP | New England Biolabs | Cat # P0753S |
| DAPI Fluoromount-G | SouthernBiotech | Cat # 0100-20 |
| Brain Heart Infusion (BHI) | BD Biosciences | Cat # 90003-040 |
| **Critical Commercial Assays** | | |
| RevertAid H Minus First Strand cDNA Synthesis Kit | Thermo Scientiﬁc | Cat # K1631 |
| Maxima® SYBR Green/Fluorescein qPCR Master Mix | Thermo Scientiﬁc | Cat # 4367659 |
| Dual-Glo Luciferase Assay System | Promega | Cat # E2940 |
| ELISA Kit for Interferon Beta (IFNb) | Cloud-clone corp | Cat # SEA222Mu |
| DuoSet ELISA mouse IL-28B/IFN-λ3 | R&D systems | Cat # DY1789 |
| DuoSet ELISA mouse IL-6 | R&D systems | Cat # DY406 |
| DuoSet ELISA mouse IL-10 | R&D systems | Cat # DY417 |
| DuoSet ELISA mouse TNF-α | R&D systems | Cat # DY410 |
| Ubiquitinylation kit | Enzo Life Science | Cat # BML-UW9920 |
| Click-iT Protein Reaction Buffer Kit | Invitrogen | Cat # C10276 |
| Click-iT AHA (L-azidohomoalanine) | Invitrogen | Cat # C10102 |
| Biotin-alkyne | Invitrogen | Cat # B10185 |
| High-Sensitivity Cardiac Troponin-I | Beckman | Cat # B52699 |
| CK | Beckman | Cat # OSR6179 |
| CK-MB | Beckman | Cat # OSR61155 |
| **Bacterial and virus strains** | | |
| *Staphylococcus aureus* (Newman strain) | Dr. Yung-Chi Chang (Graduate Institute of Microbiology, National Taiwan University College of Medicine) |  |
| Encephalomyocariditis virus | Dr. Lih-Hwa Hwang (National Yang-Ming University, Taiwan) |  |
| SARS-CoV-2 virus (NTU03/TWN/human/2020) | (Cheng et al., 2020) | Accession ID EPI_ISL_413592 |
| Sendai virus | Dr. Helene Minyi Liu (Graduate Institute of Biochemistry and Molecular Biology, National Taiwan University College of Medicine) |  |
| **Experimental models: cell lines** | | |
| HEK293T | ATCC | CRL-3216 |
| RAW264.7 | ATCC | TIB-71 |
| Vero | Dr. Lih-Hwa Hwang (National Yang-Ming University, Taiwan) |  |
| Calu-3 | Dr. Helene Minyi Liu (Graduate Institute of Biochemistry and Molecular Biology, National Taiwan University College of Medicine) |  |
| CAL-1 | Dr. Kuo-I Lin (Genomics Research Center, Academia Sinica) |  |
| **Experimental Models: Organisms/Strains** | | |
| Mouse WT: C57BL/6J | The Jackson Laboratory |  |
| Mouse *Tlr3* ^t/t^ C57BL/6J | (Chen et al., 2021) |  |
| **Software and Algorithms** | | |
| GraphPad Prism 8 | Graphpad Inc | RRID: SCR_002798 |
| Fiji (ImageJ) | https://imagej.nih.gov/ij/ | RRID: SCR_001935 |
| FLowjo v10 | Treestar Inc | RRID: SCR_008520 |
| Microsoft Excel | Microsoft | RRID: SCR_016137 |
| **Plasmids** | | |
| pUNO1-HA-mTLR3 | Invivogen | Cat # puno1ha-mtlr3-dn |
| Lentiviral-based pLVX-AcGFP1-N1 | Clontech Laboratories | Cat # 632154 |
| pLVX-WT-mTLR3-AcGFP-N1 | This paper | N/A |
| pLVX-WT-mTLR3 (K813R)-AcGFP-N1 | This paper | N/A |
| pLVX-WT-mTLR7-AcGFP-N1 | This paper | N/A |
| pLVX-WT-mTLR9-AcGFP-N1 | This paper | N/A |
| Hox-Express Plasmid | (Redecke et al., 2013) | N/A |
| Packaging Plasmid: (Ecotropic (783)) | (Redecke et al., 2013) | N/A |
| pMD.G | National RNAi Core Facility, Academia Sinica, Taiwan | N/A |
| pCMVR8.91 | National RNAi Core Facility, Academia Sinica, Taiwan | N/A |
| pAll-Cas9.pPuro all-in-one CRISPR/Cas expression system | National RNAi Core Facility, Academia Sinica, Taiwan | N/A |
| pAll-Cas9. pPuro sgZNRF1#1 | (Shen et al., 2021) | N/A |
| pAll-Cas9. pPuro sgZNRF1#2 | (Shen et al., 2021) | N/A |
| pLKO AS2 shLuc | (Lee et al., 2017) | N/A |
| pLKO AS2 shZNRF1 #745 | (Lee et al., 2017) | N/A |
| pLKO AS2 shZNRF1 #746 | (Lee et al., 2017) | N/A |
| pAS4.1w.Pbsd-aOn lentivirus-based Tet-on system vector | National RNAi Core Facility, Academia Sinica, Taiwan | N/A |
| pAS4.1w. Pbsd-aOn WT-ZNRF1 | This paper | N/A |
| pAS4.1w. Pbsd-aOn ZNRF1 (C184A) | This paper | N/A |
| pAS4.1w. Pbsd-aOn ZNRF1 (Y103F) | This paper | N/A |
| pLKO.AS2.neo | (Lee et al., 2017) | N/A |
| pLKO.AS2.WT-ZNRF1-Flag | (Lee et al., 2017) | N/A |
| pLKO.AS2.ZNRF1 (C184A)-Flag | (Lee et al., 2017) | N/A |
| pLKO.AS2.ZNRF1 (Y103F)-Flag | This paper | N/A |
| pcDNA3.1 Vector | (Lee et al., 2017) | N/A |
| pcDNA3.1 WT-ZNRF1-Flag | (Lee et al., 2017) | N/A |
| pcDNA3.1 ZNRF1 (C184A)-Flag | (Lee et al., 2017) | N/A |
| pcDNA3.1 ZNRF1 (Y103F)-Flag | This paper | N/A |
| pcDNA 3.1 ZNRF1 (N-ter+Zinc)-Flag | (Lee et al., 2017) | N/A |
| pcDNA3.1 ZNRF1 (ΔZinc)-Flag | (Lee et al., 2017) | N/A |
| pcDNA3.1 ZNRF1 (Zinc+RING)-Flag | (Lee et al., 2017) | N/A |
| pcDNA3.1 ZNRF1-myc | This paper | N/A |
| pcDNA HA-K6 | Dr. Chihiro Sasakawa | N/A |
| pcDNA HA-K11 | Dr. Chihiro Sasakawa | N/A |
| pcDNA HA-K27 | Dr. Chihiro Sasakawa | N/A |
| pcDNA HA-K29 | Dr. Chihiro Sasakawa | N/A |
| pcDNA HA-K33 | Dr. Chihiro Sasakawa | N/A |
| pcDNA HA-K48 | Dr. Chihiro Sasakawa | N/A |
| pcDNA HA-K48R | Dr. Chihiro Sasakawa | N/A |
| pcDNA HA-K63 | Dr. Chihiro Sasakawa | N/A |
| pcDNA HA-K63R | Dr. Chihiro Sasakawa | N/A |
| pcDNA HA-ubiquitin | Dr. Chihiro Sasakawa | N/A |
| pGL4-IFN-β-Luc firefly luciferase vector | Dr. Pin Ling | N/A |
| pELAM-NFκB-Luc firefly luciferase vector | Dr. Pin Ling | N/A |
| pRL-TK *Renilla* luciferase vector | Promega | Cat # E2241 |
| pEGFP-N1_mZnrf1 | This paper | N/A |
| pFlag-CMV-1-hTLR1 | This paper | N/A |
| pFlag-CMV-1-hTLR2 | This paper | N/A |
| pFlag-CMV-1-hTLR3 | This paper | N/A |
| pFlag-CMV-1-hTLR4 | This paper | N/A |
| pFlag-CMV-1-hTLR7 | This paper | N/A |
| pFlag-CMV-1-hTLR9 | This paper | N/A |
| pRK5-Myc WT-Src | Dr. Ruey-Hwa Chen | N/A |
| pLKO.AS2.neo.HRS-Flag |  | N/A |
| pCMV-Flag-RIG-I | Dr. Helene Minyi Liu (Graduate Institute of Biochemistry and Molecular Biology, National Taiwan University College of Medicine) | N/A |
| pCMV-Flag-MDA5 | Dr. Helene Minyi Liu (Graduate Institute of Biochemistry and Molecular Biology, National Taiwan University College of Medicine) | N/A |
