# Supplementary figures and images for: The Src–ZNRF1 axis controls TLR3 trafficking and interferon responses to limit lung barrier damage
Source: J Exp Med. 2023 May 9;220(8):e20220727. doi: 10.1084/jem.20220727 (PMC10174191; doi:10.1084/jem.20220727)

Source Data Figure 1B

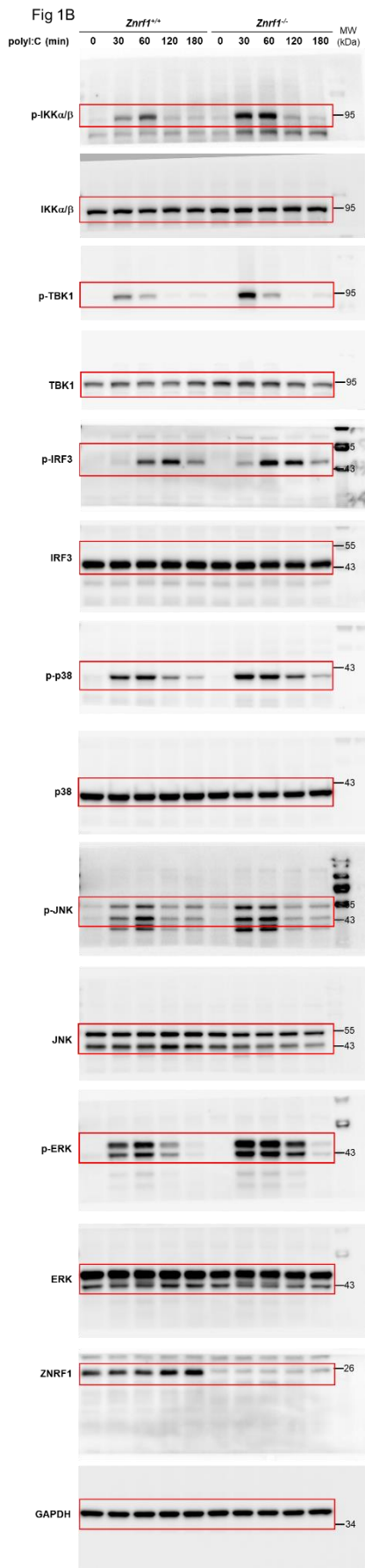

Source Data Figure 1F

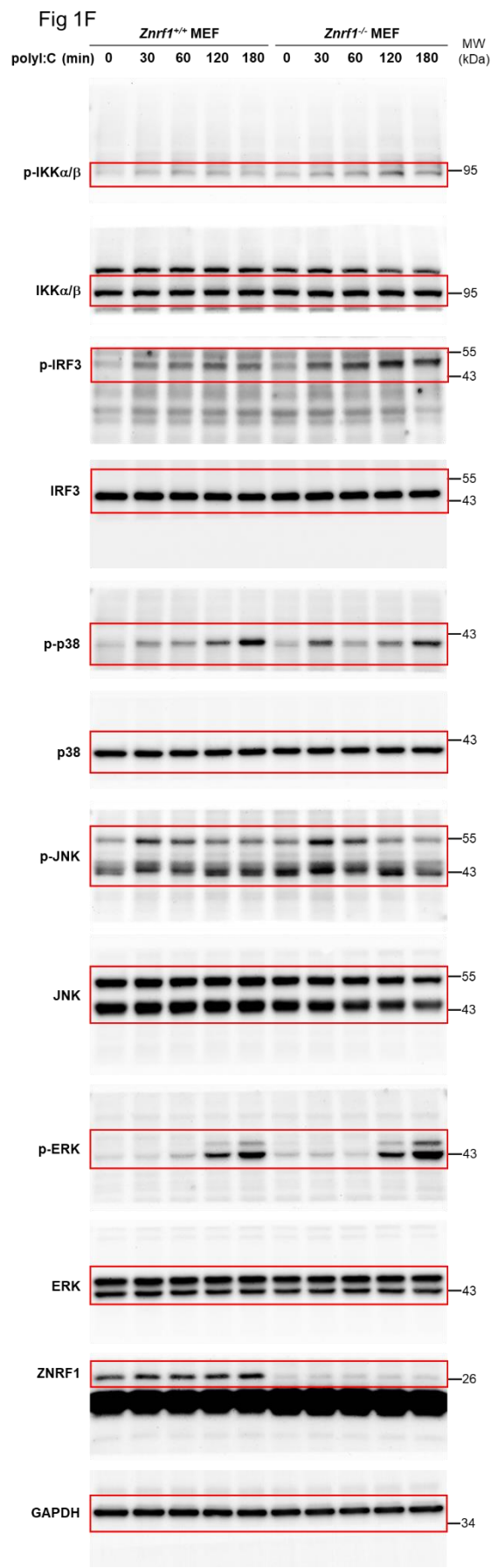

Source Data Figure 1G

Fig. 1G

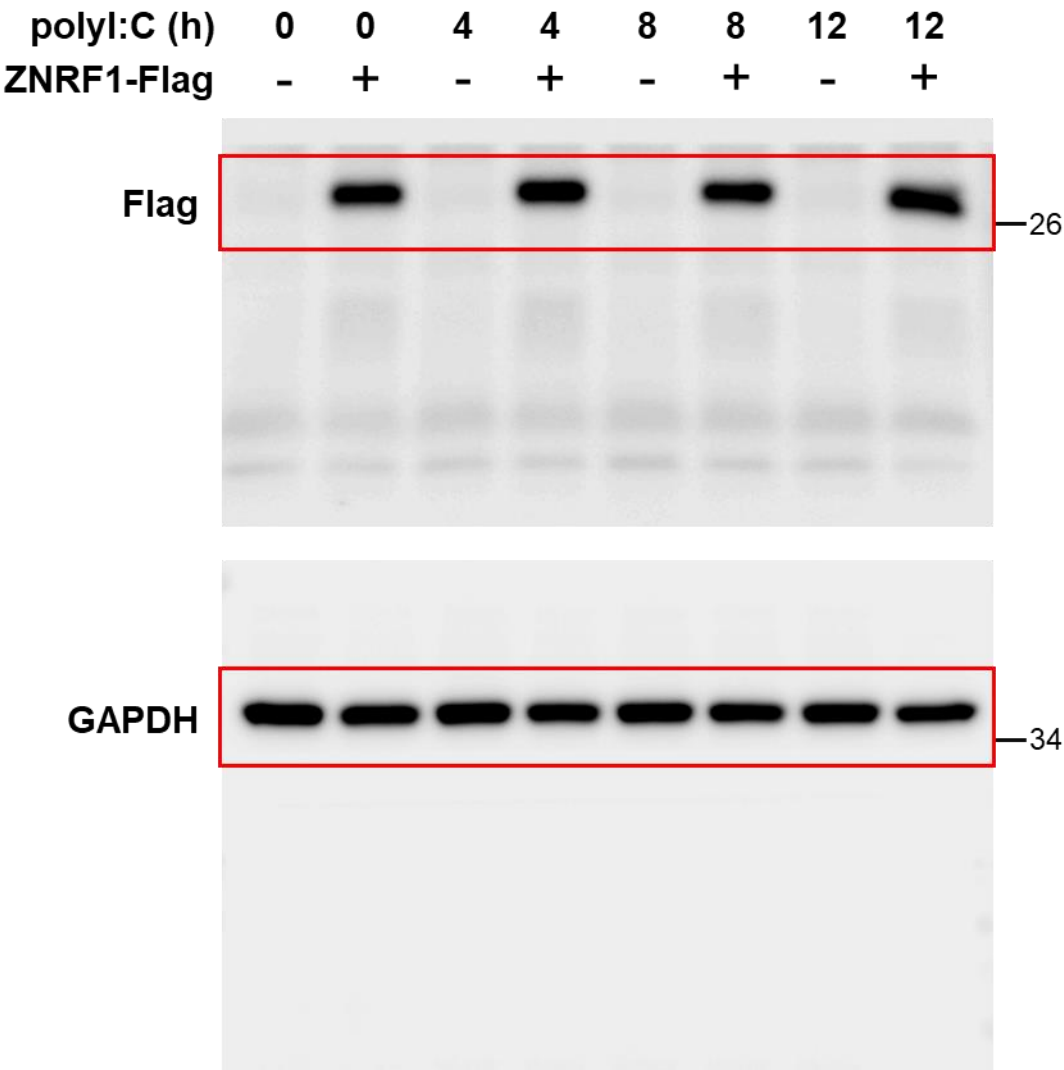

Source Data Figure 1H

Fig. 1H

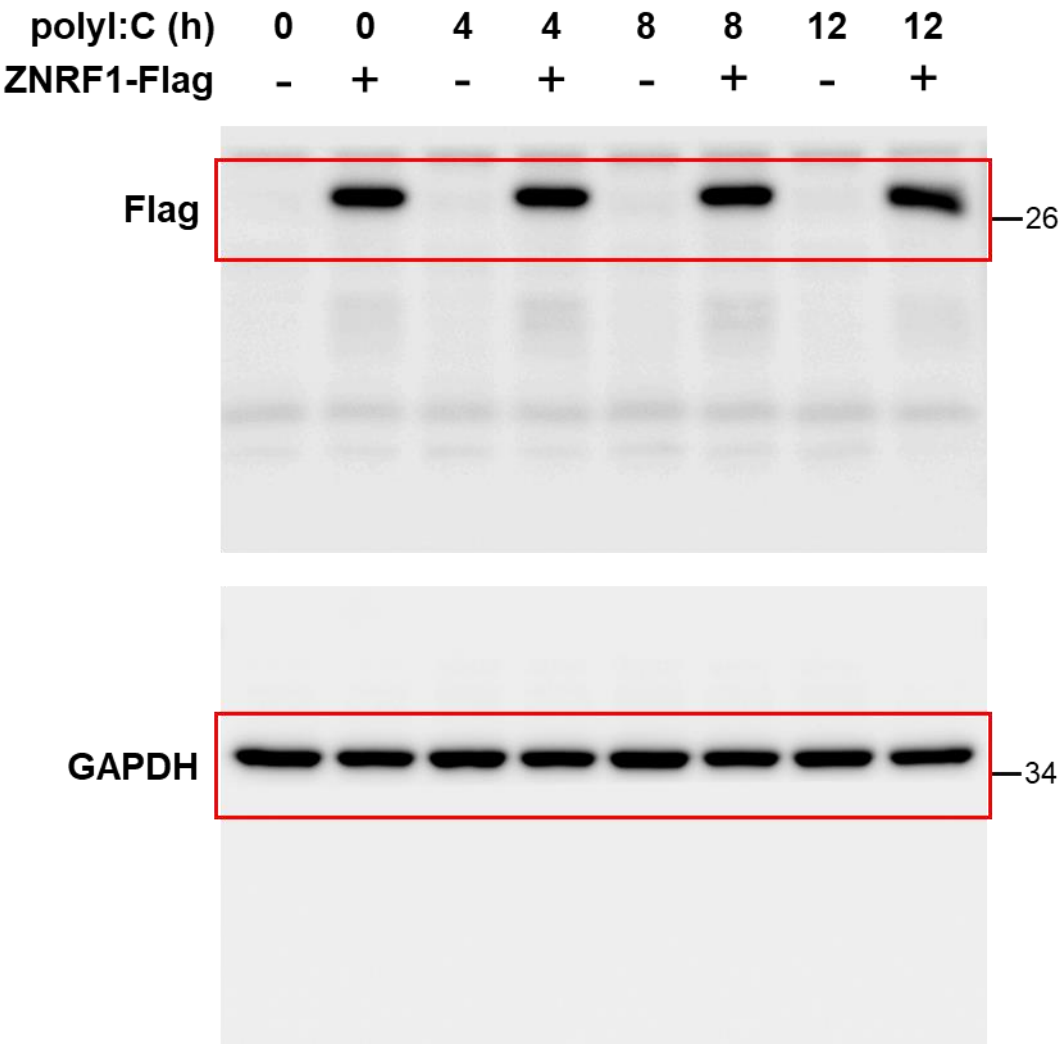

Supplement: SourceData F1 — is the source file for Fig. 1. [file JEM_20220727_SourceDataF1.pdf]

Source Data Figure 3D

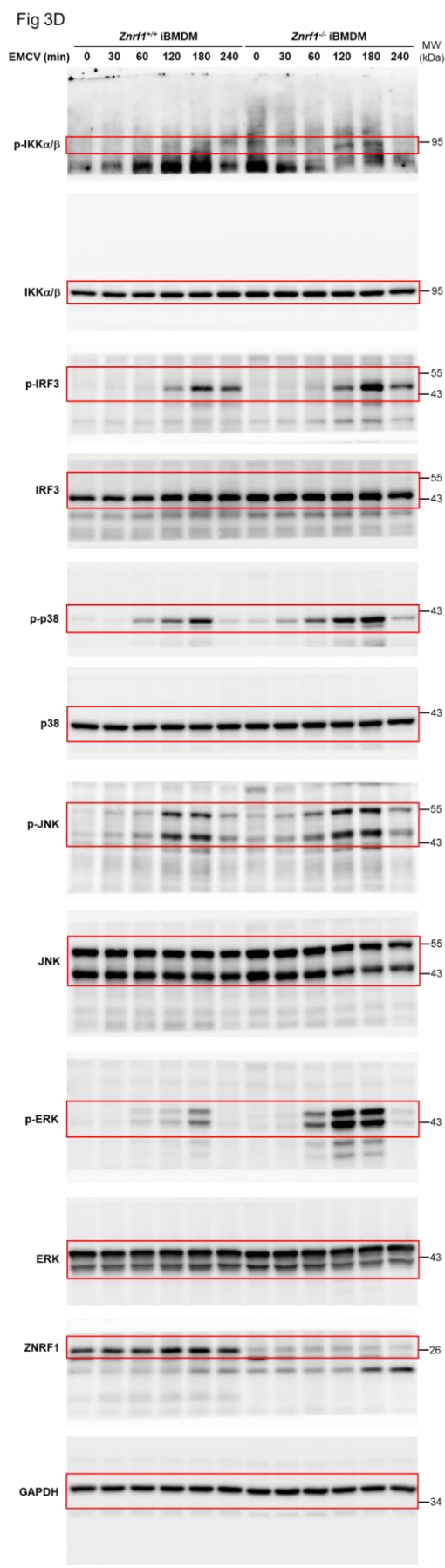

Supplement: SourceData F3 — is the source file for Fig. 3. [file JEM_20220727_SourceDataF3.pdf]

# Source Data Figure 4B

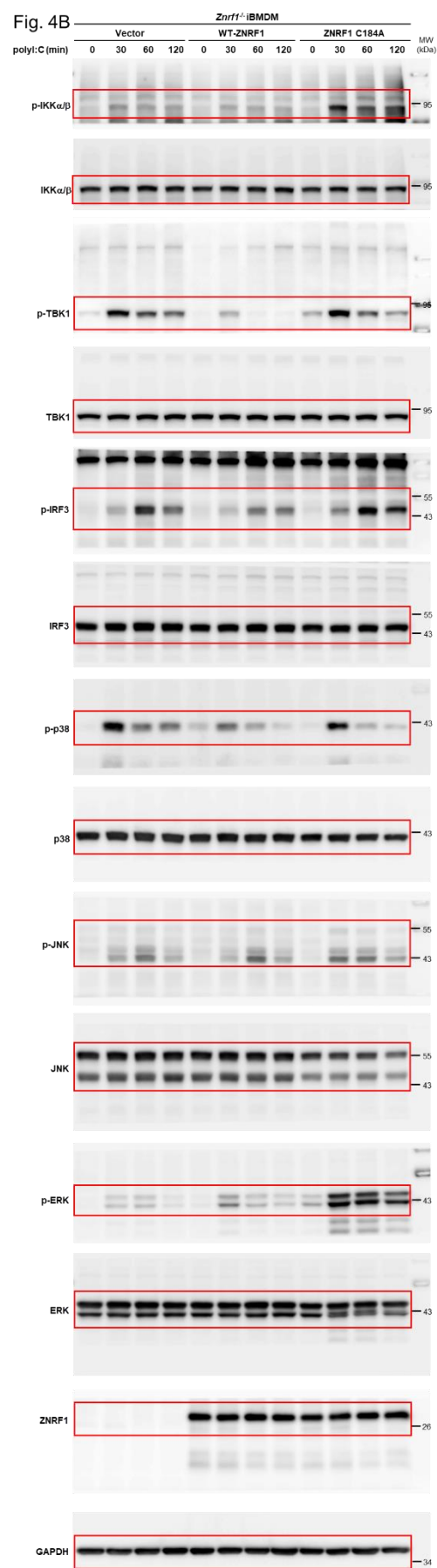

Source Data Figure 4D

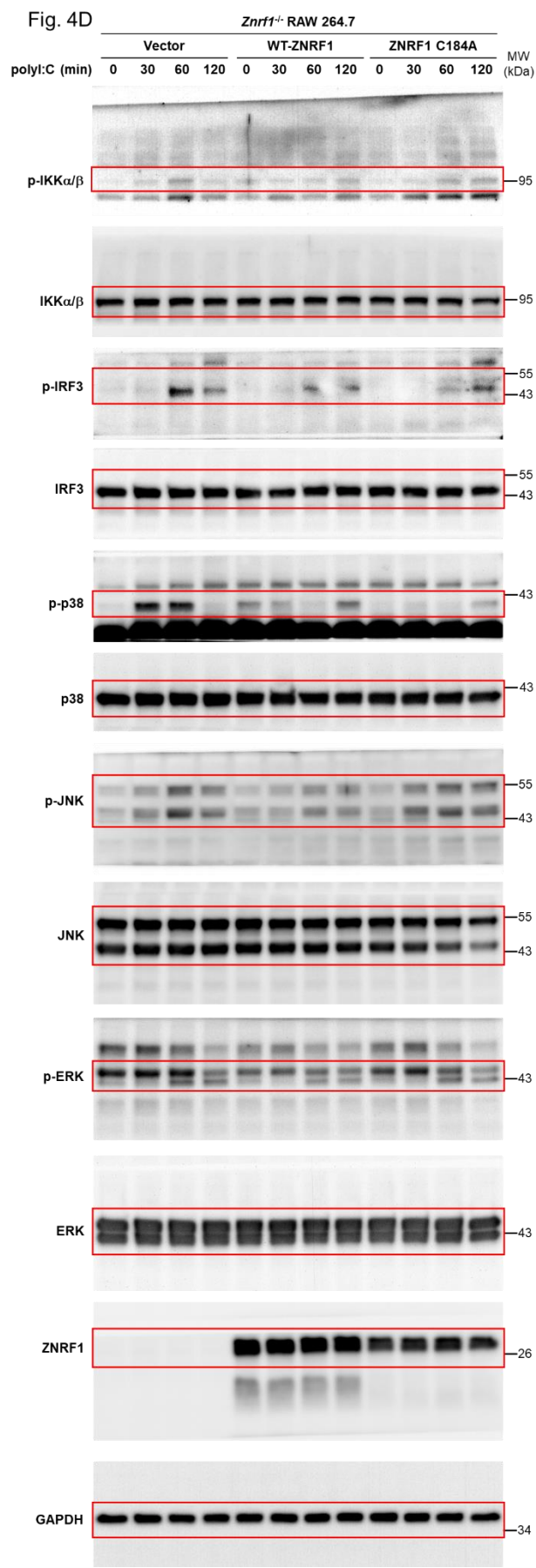

Supplement: SourceData F4 — is the source file for Fig. 4. [file JEM_20220727_SourceDataF4.pdf]

Source Data Figure 5B

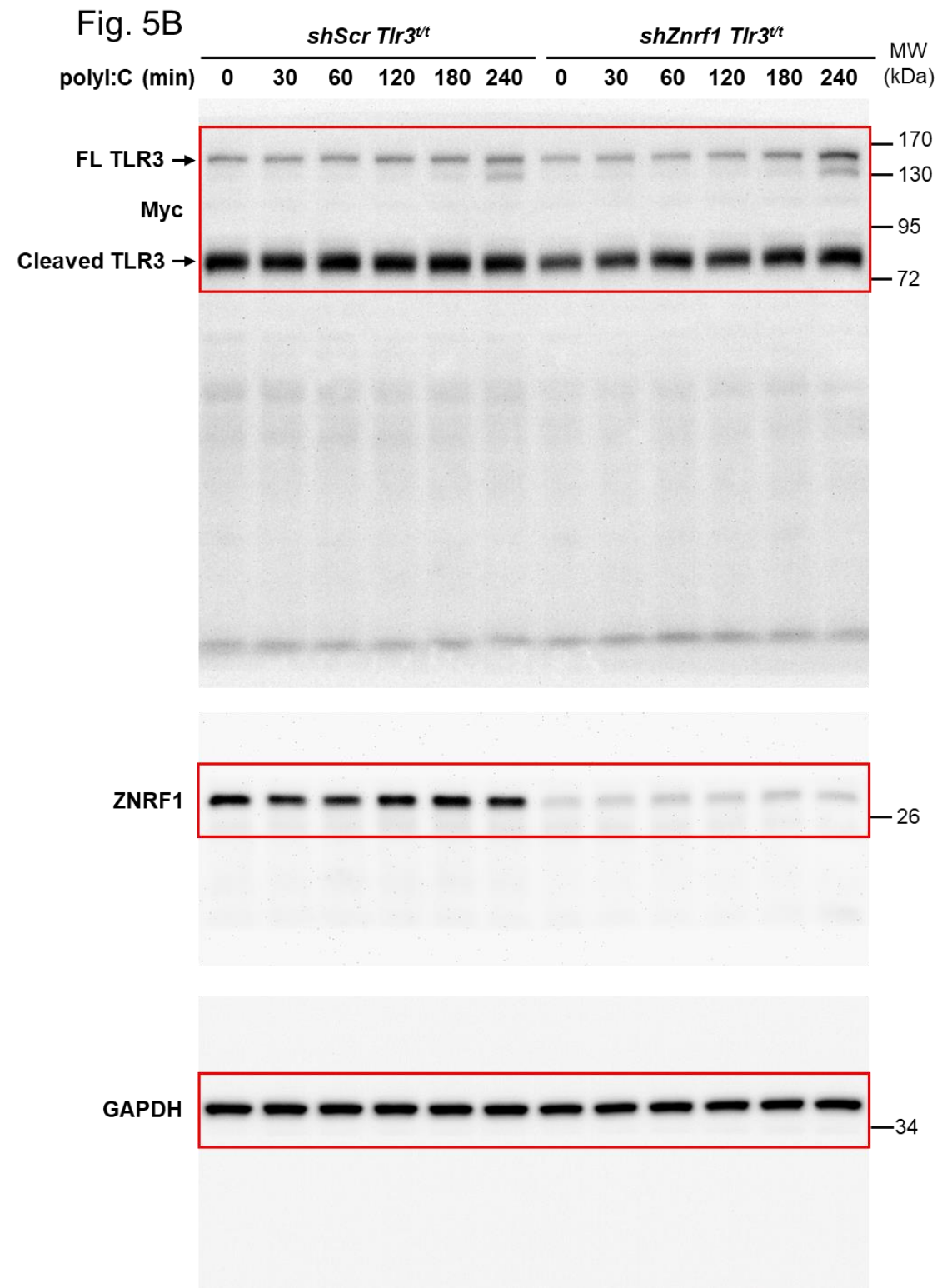

Supplement: SourceData F5 — is the source file for Fig. 5. [file JEM_20220727_SourceDataF5.pdf]
