# Supplementary material for: The Src–ZNRF1 axis controls TLR3 trafficking and interferon responses to limit lung barrier damage
Source: J Exp Med. 2023 May 9;220(8):e20220727. doi: 10.1084/jem.20220727 (PMC10174191; doi:10.1084/jem.20220727)

Source Data Figure 6C

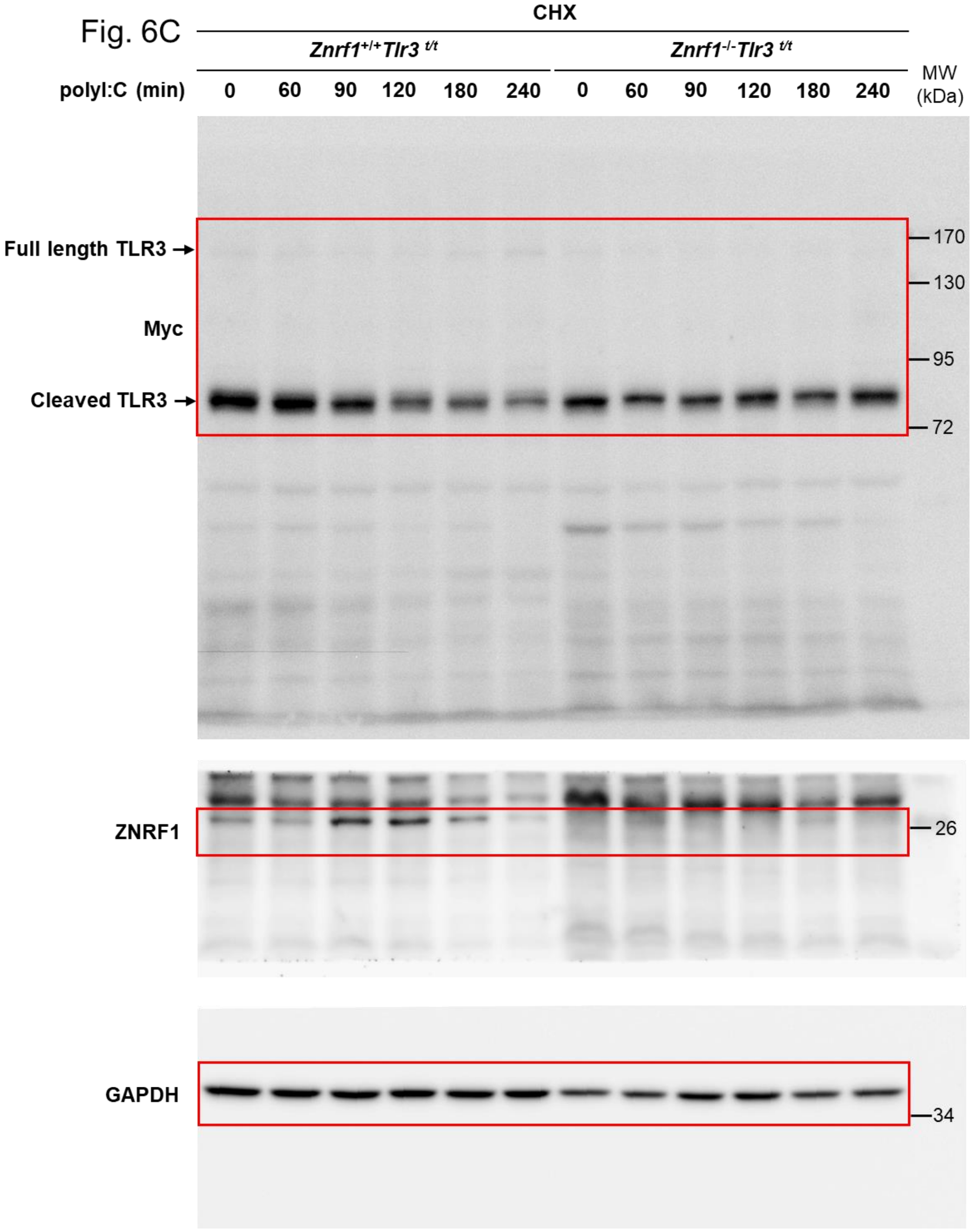

Source Data Figure 6E

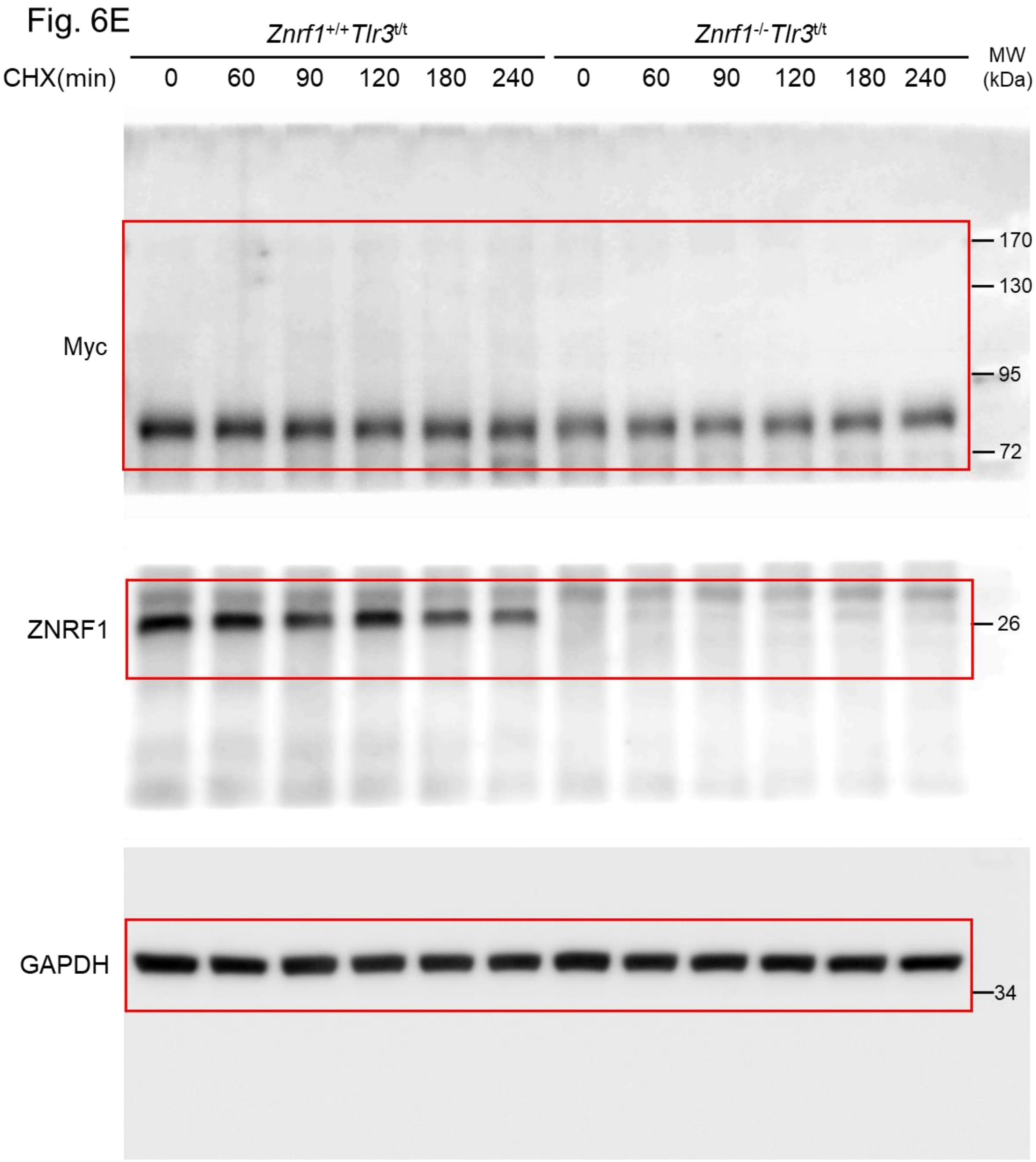

Source Data Figure 6G

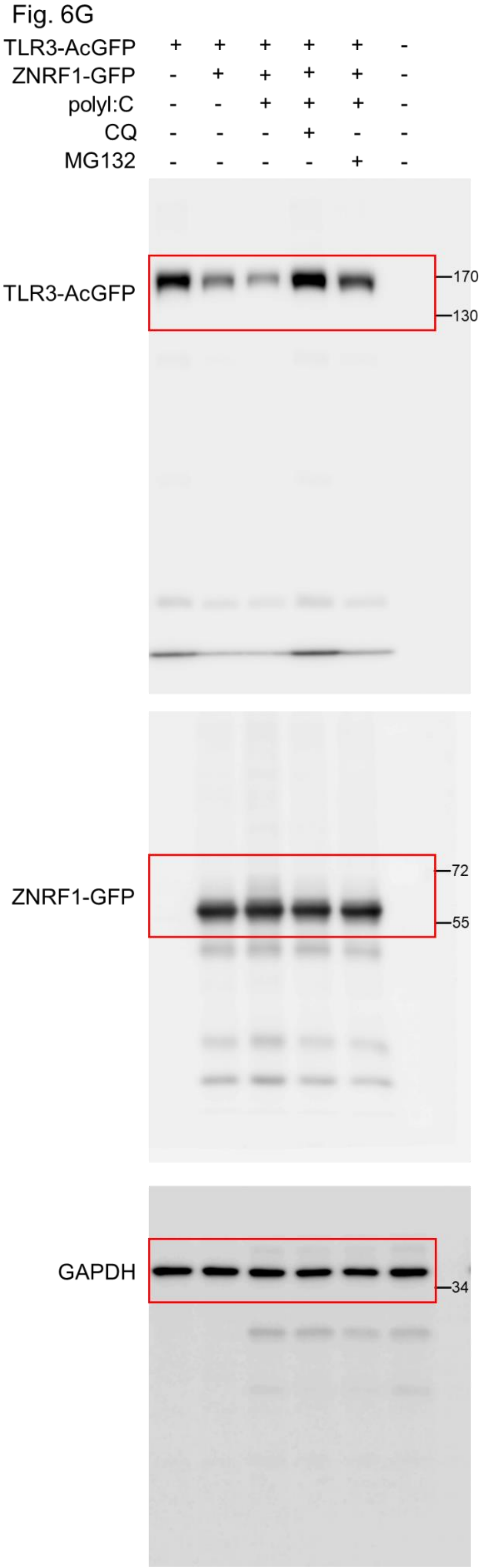

Source Data Figure 6H

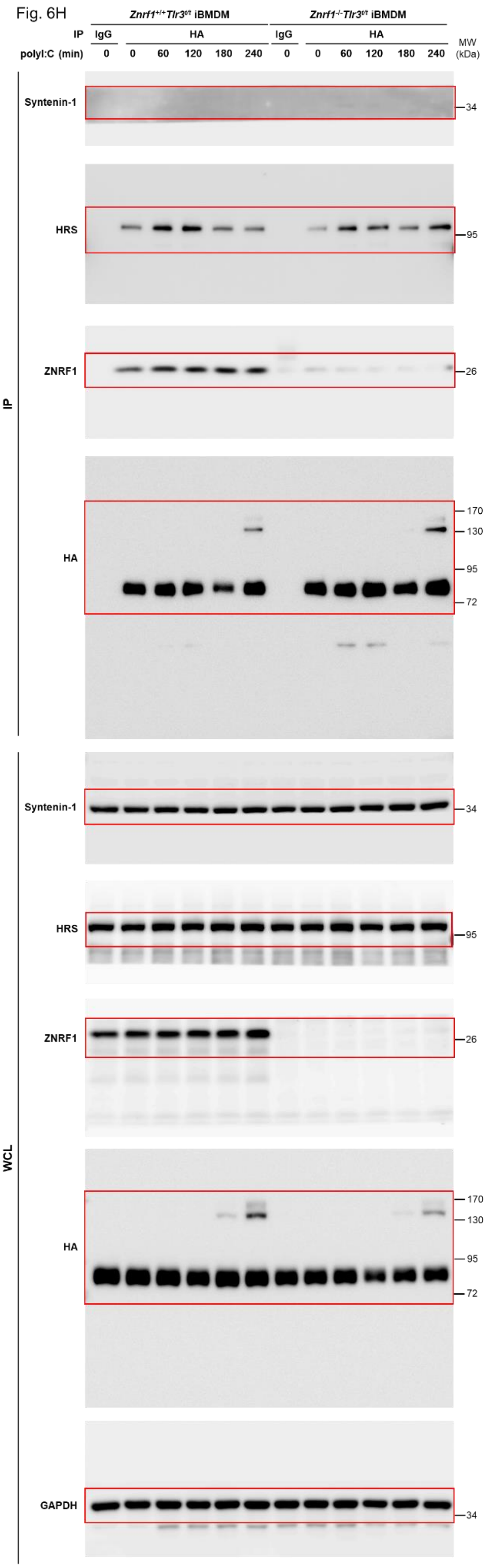

Supplement: SourceData F6 — is the source file for Fig. 6. [file JEM_20220727_SourceDataF6.pdf]
