# Supplementary material for: The Src–ZNRF1 axis controls TLR3 trafficking and interferon responses to limit lung barrier damage
Source: J Exp Med. 2023 May 9;220(8):e20220727. doi: 10.1084/jem.20220727 (PMC10174191; doi:10.1084/jem.20220727)

Source Data Figure 7A

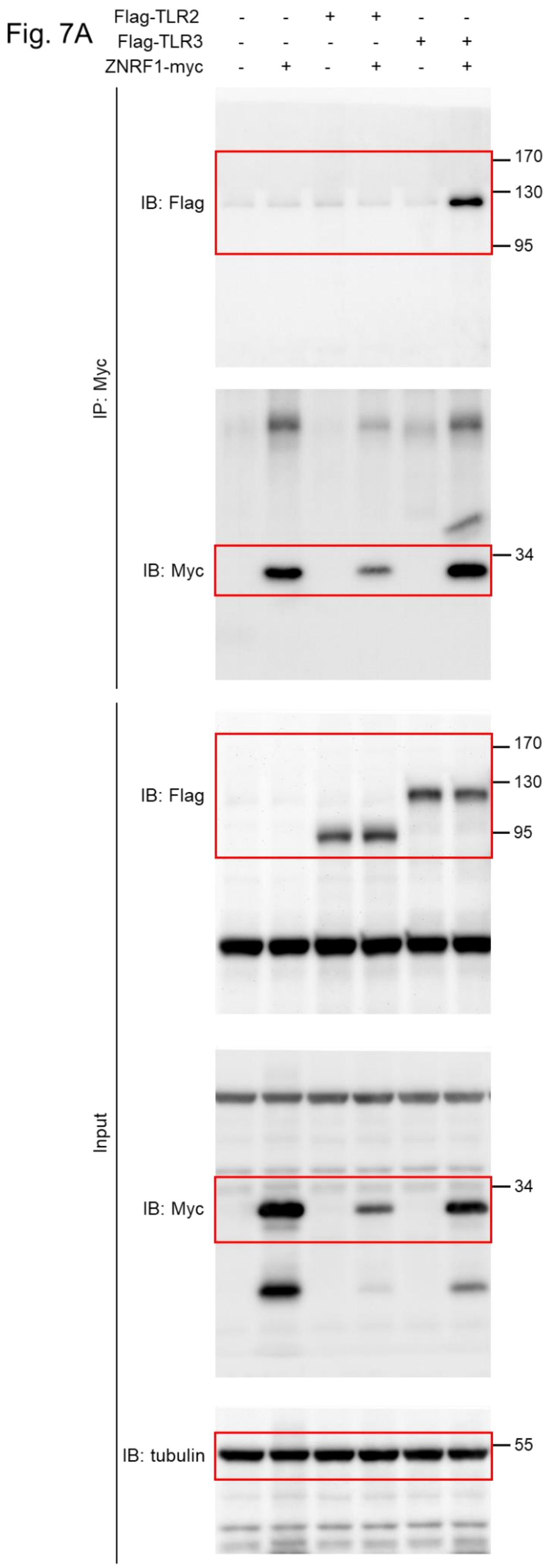

Source Data Figure 7B

Fig. 7B

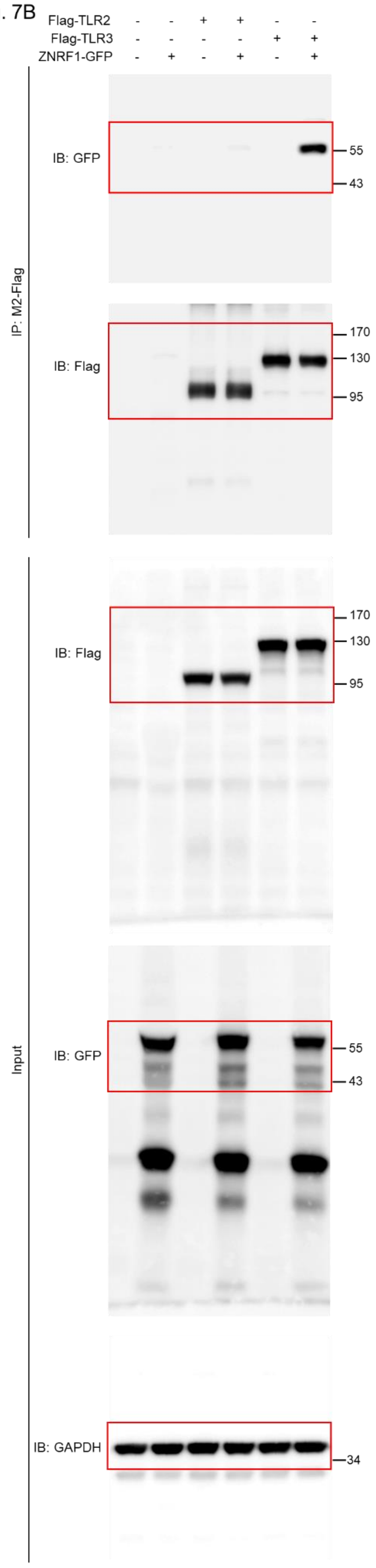

Source Data Figure 7C

Fig. 7C

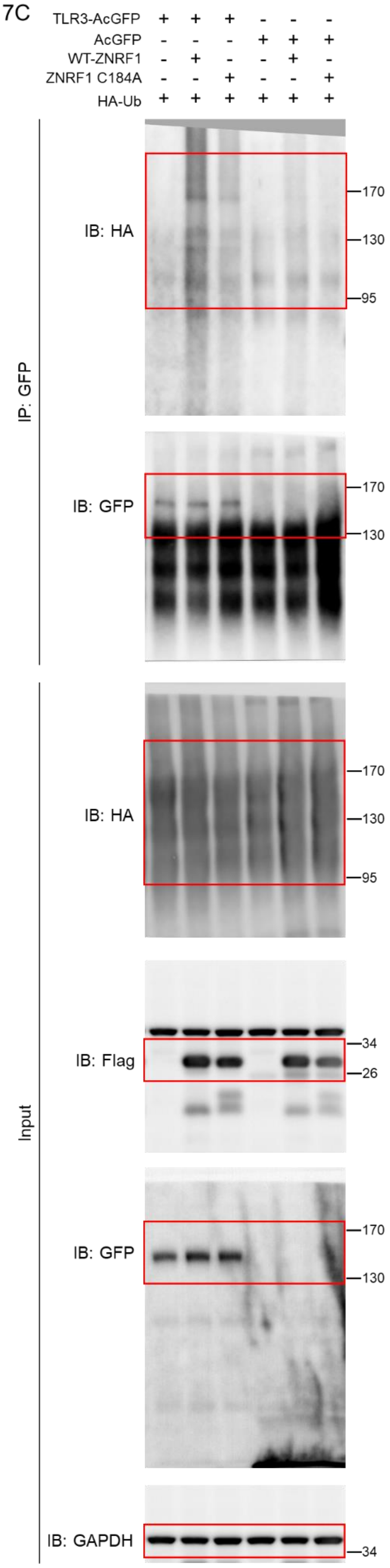

Source Data Figure 7D

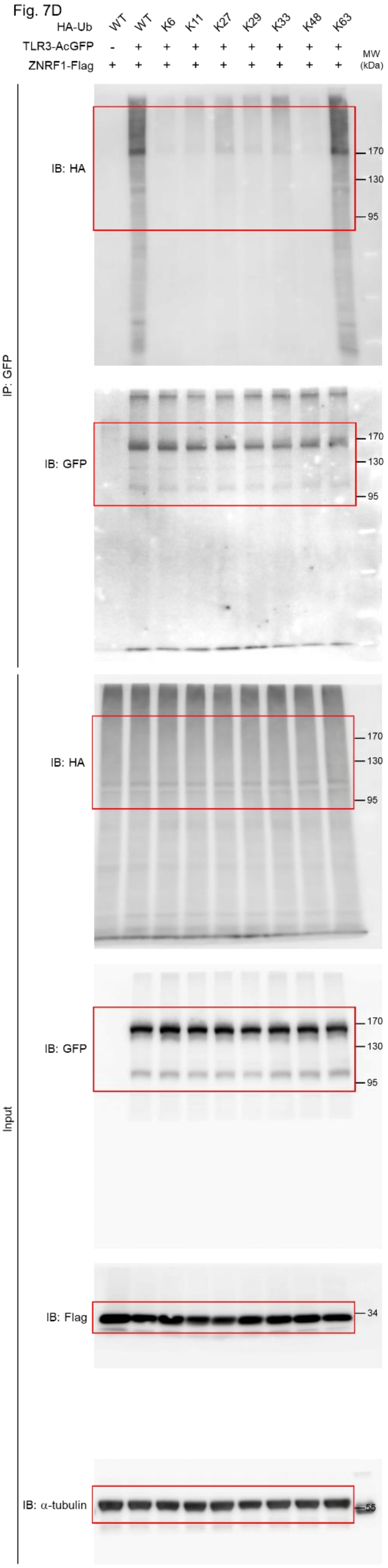

Source Data Figure 7E

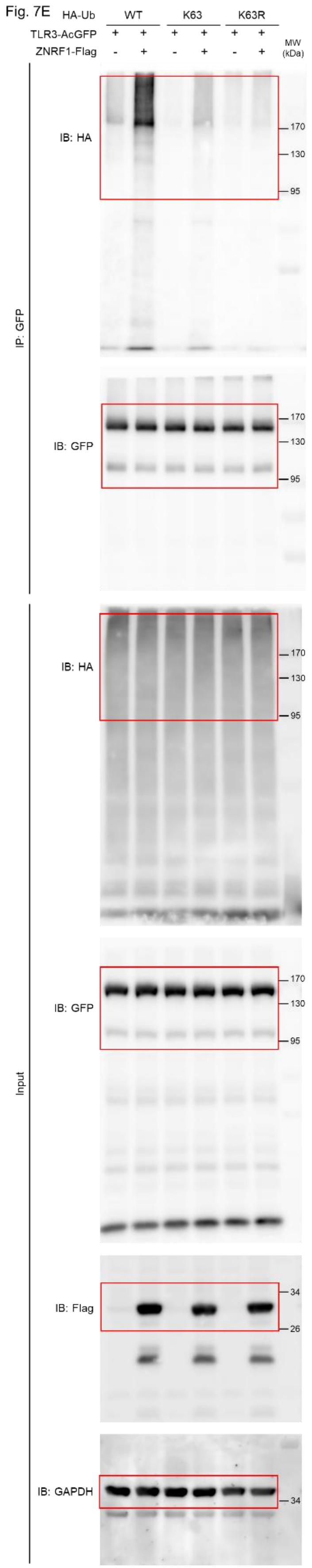

Source Data Figure 7F

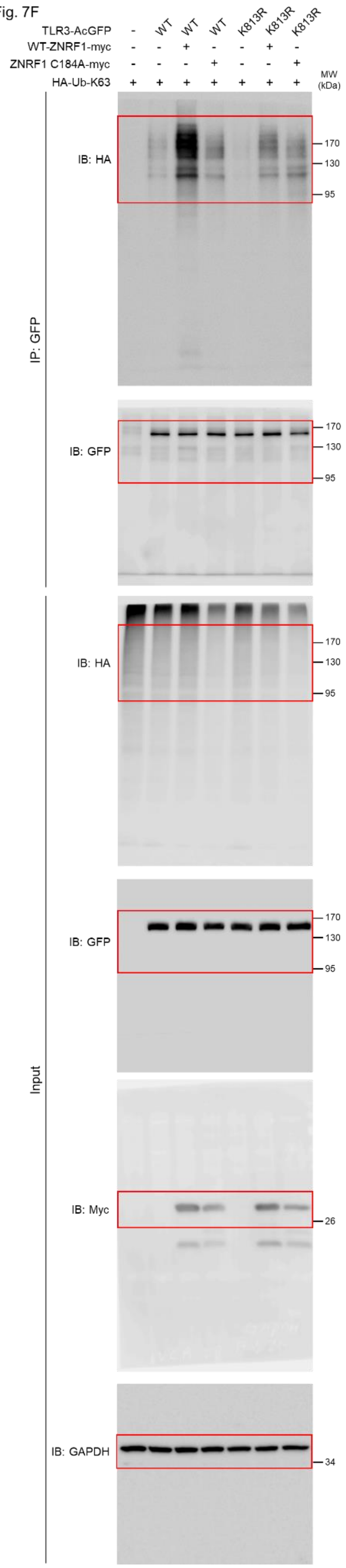

Source Data Figure 7G

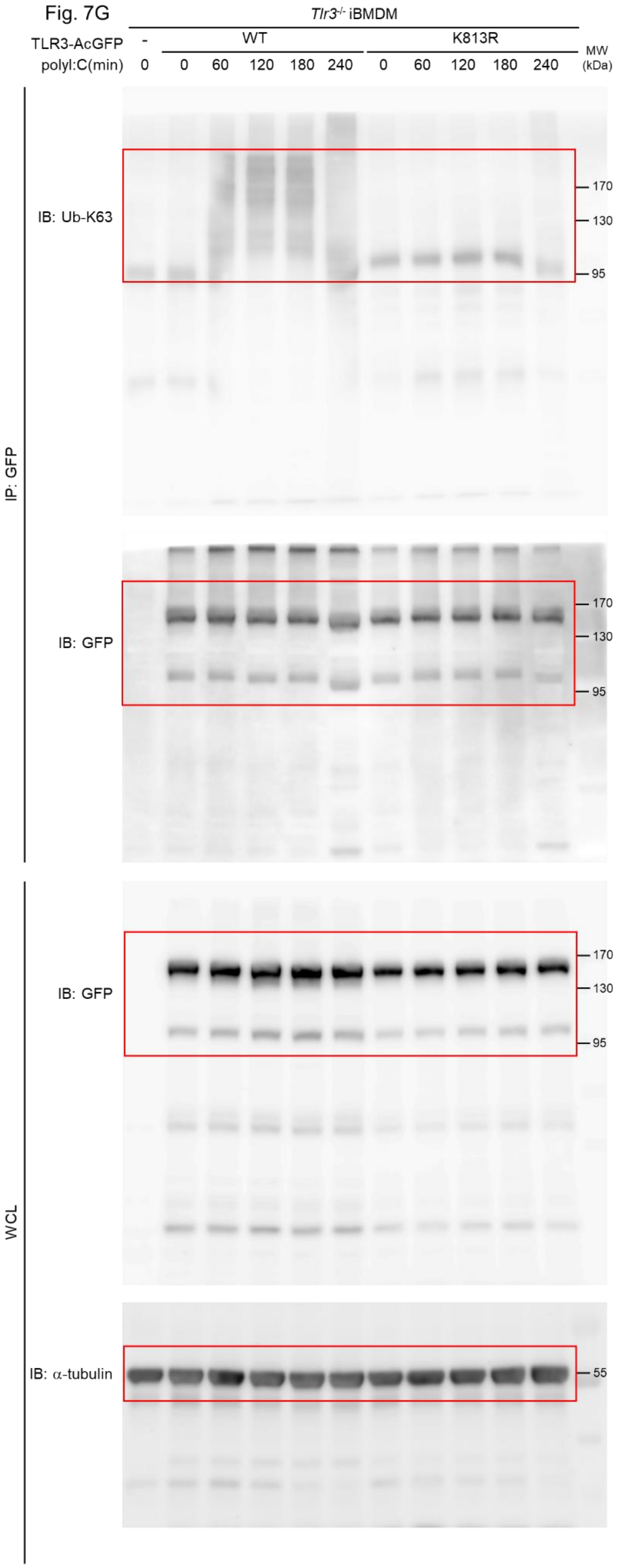

Supplement: SourceData F7 — is the source file for Fig. 7. [file JEM_20220727_SourceDataF7.pdf]
