# Supplementary material for: The Src–ZNRF1 axis controls TLR3 trafficking and interferon responses to limit lung barrier damage
Source: J Exp Med. 2023 May 9;220(8):e20220727. doi: 10.1084/jem.20220727 (PMC10174191; doi:10.1084/jem.20220727)

Source Data Figure 8C

Fig. 8C

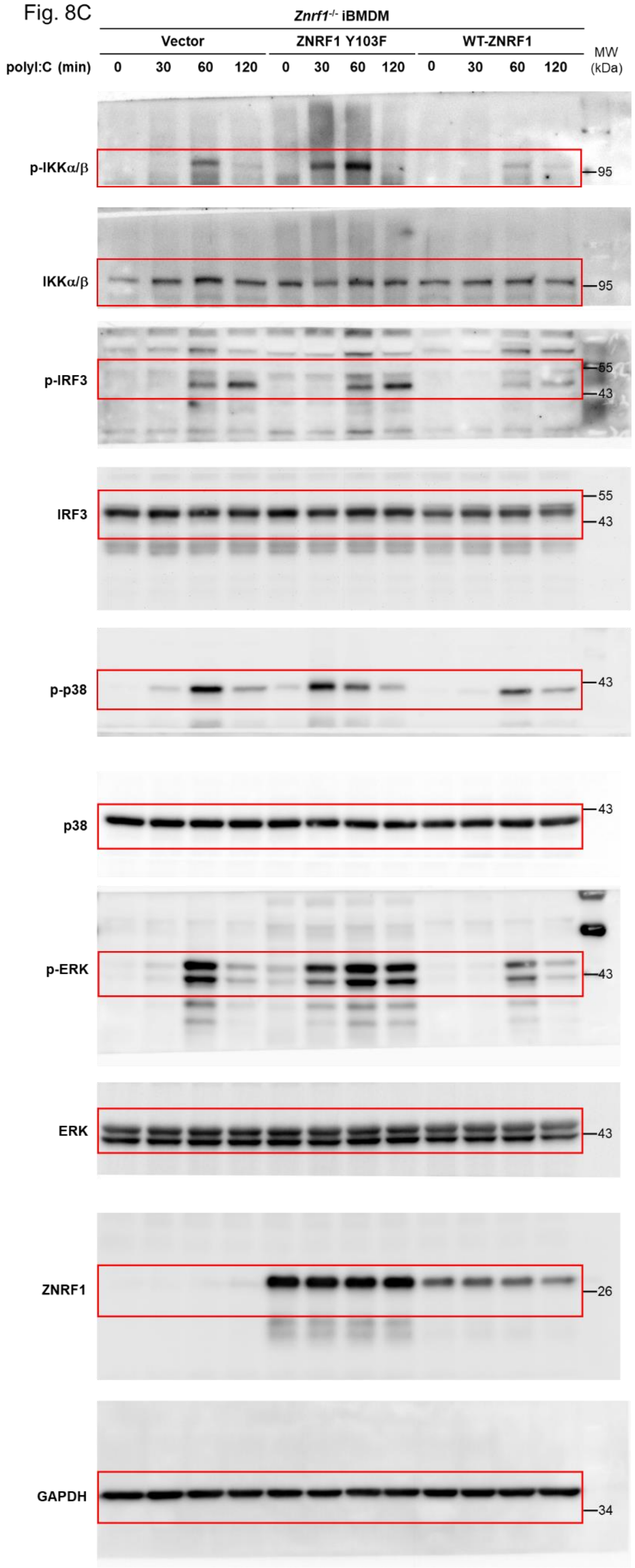

Source Data Figure 8D

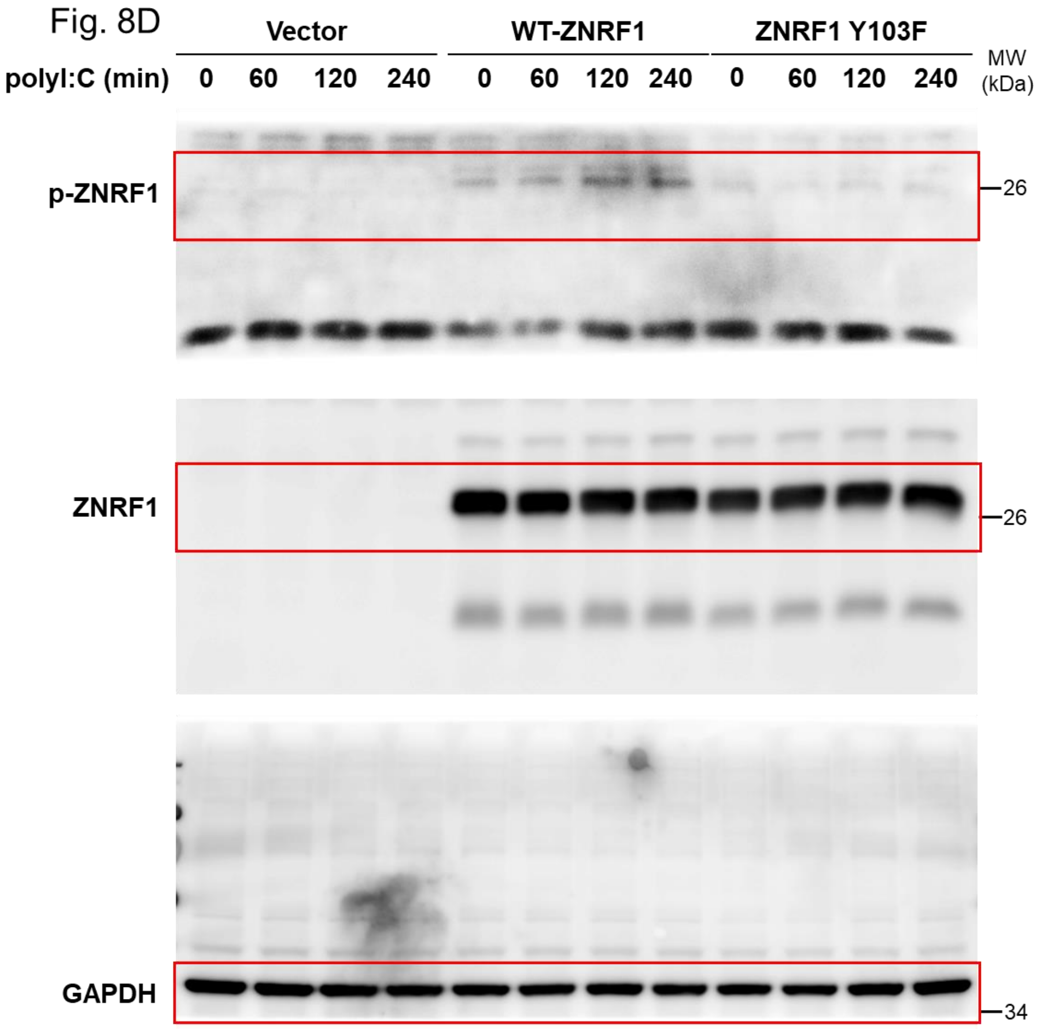

Source Data Figure 8E

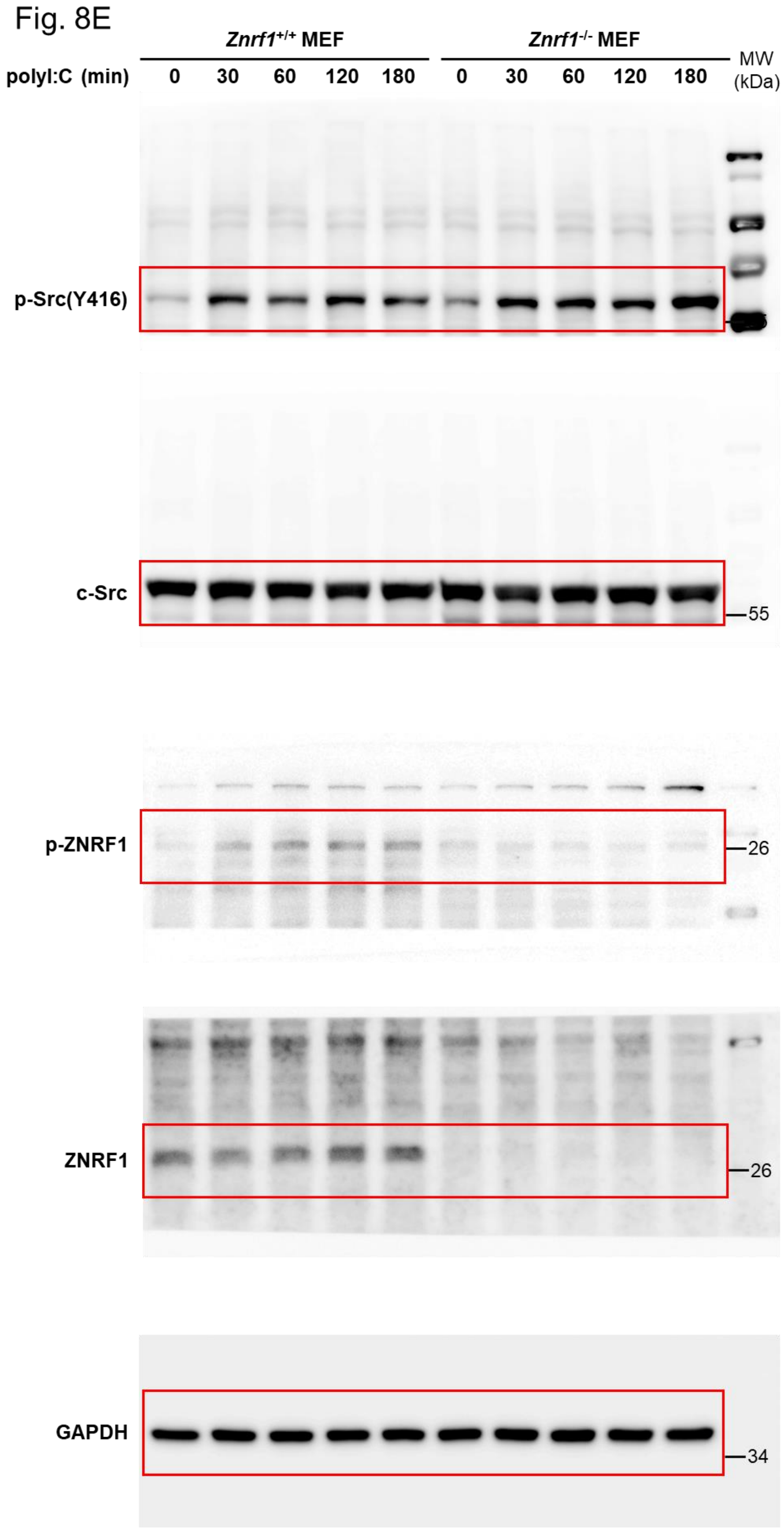

Source Data Figure 8F

Fig. 8F

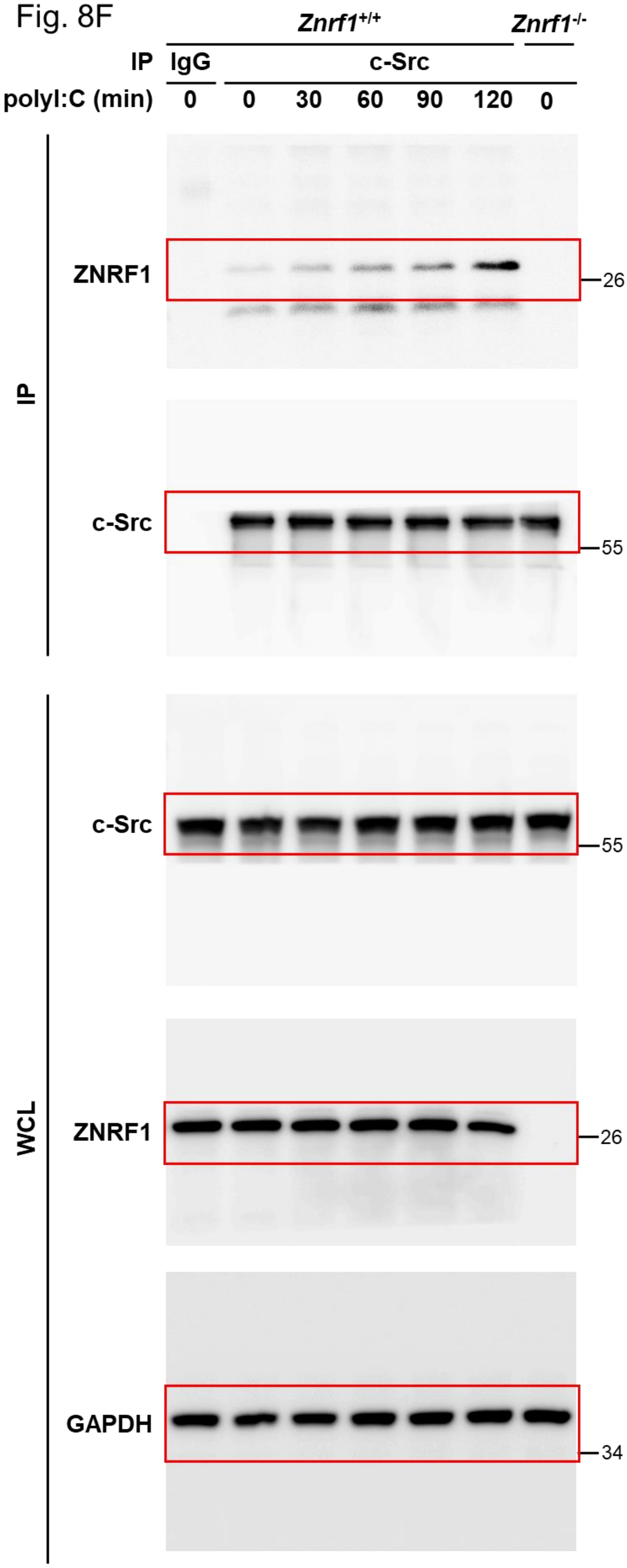

Source Data Figure 8G

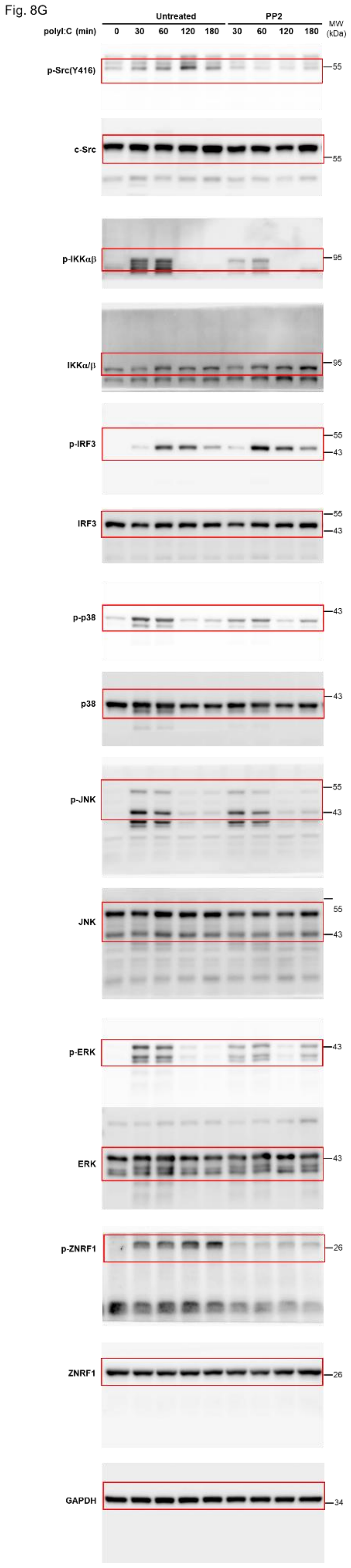

Source Data Figure 8H

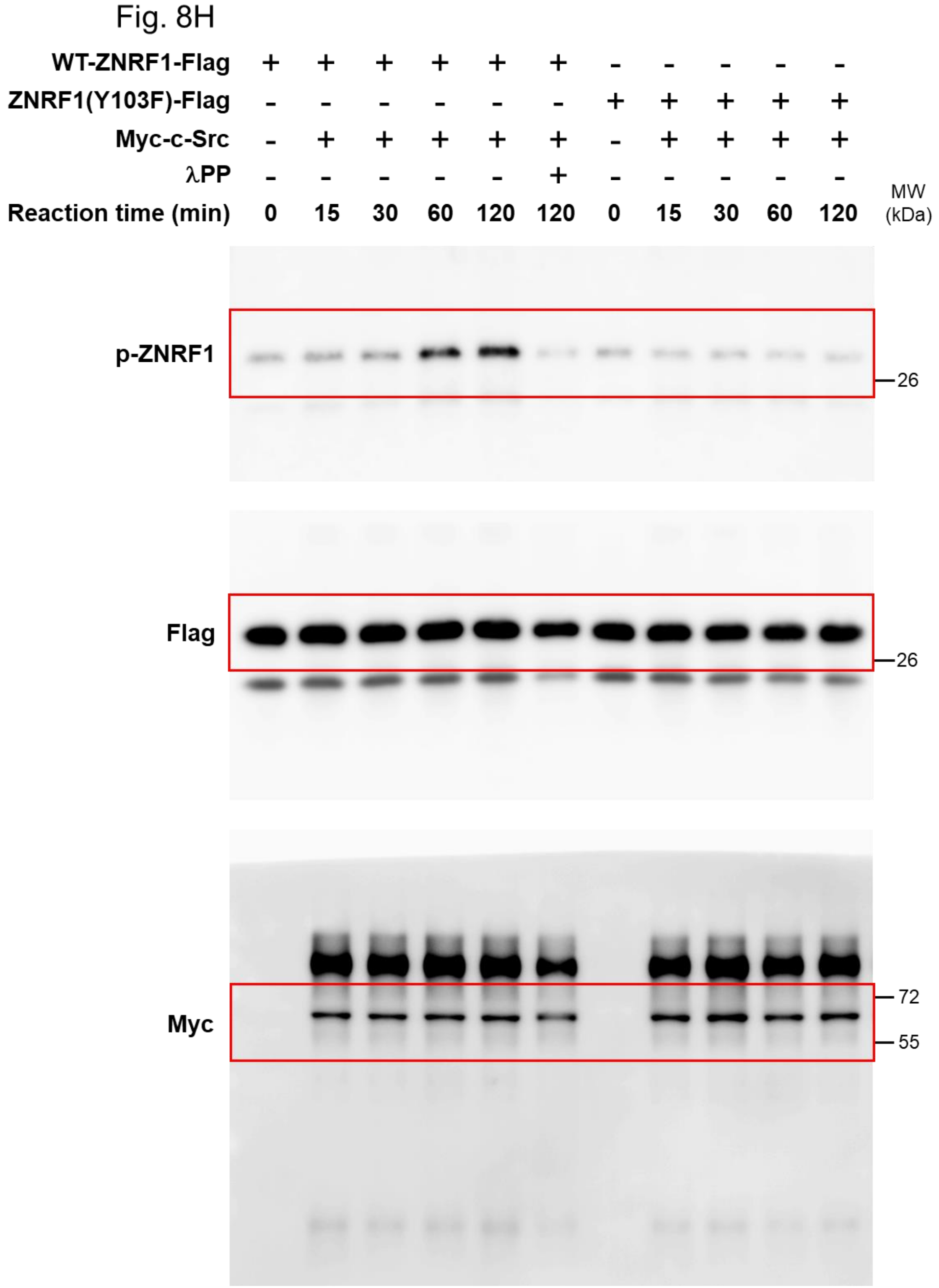

Source Data Figure 8I

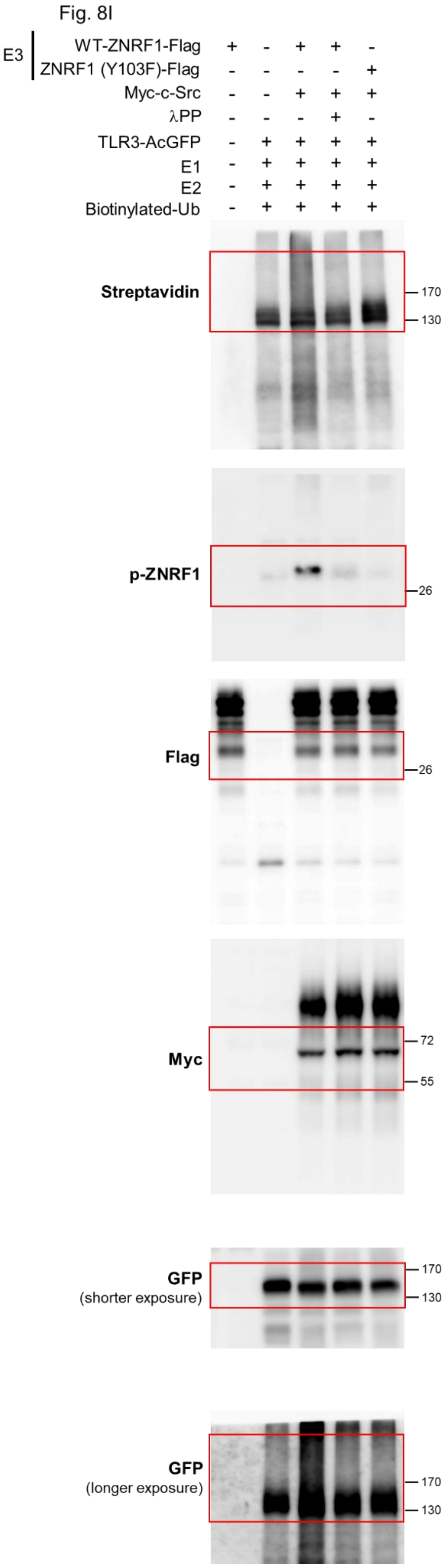

Supplement: SourceData F8 — is the source file for Fig. 8. [file JEM_20220727_SourceDataF8.pdf]
