# Supplementary material for: The Src–ZNRF1 axis controls TLR3 trafficking and interferon responses to limit lung barrier damage
Source: J Exp Med. 2023 May 9;220(8):e20220727. doi: 10.1084/jem.20220727 (PMC10174191; doi:10.1084/jem.20220727)

Source Data Supplementary Figure 1A

Fig. S1A

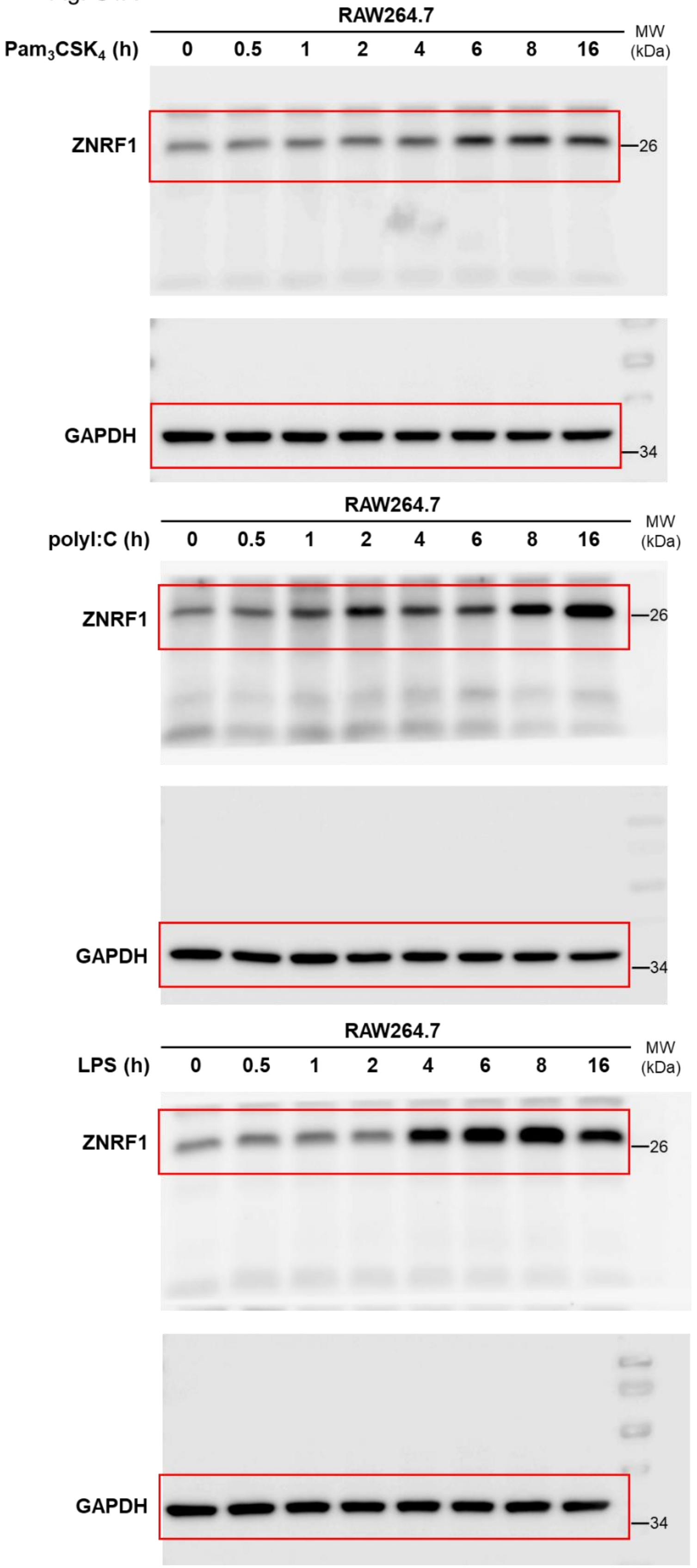

Source Data Supplementary Figure 1C

Fig. S1C

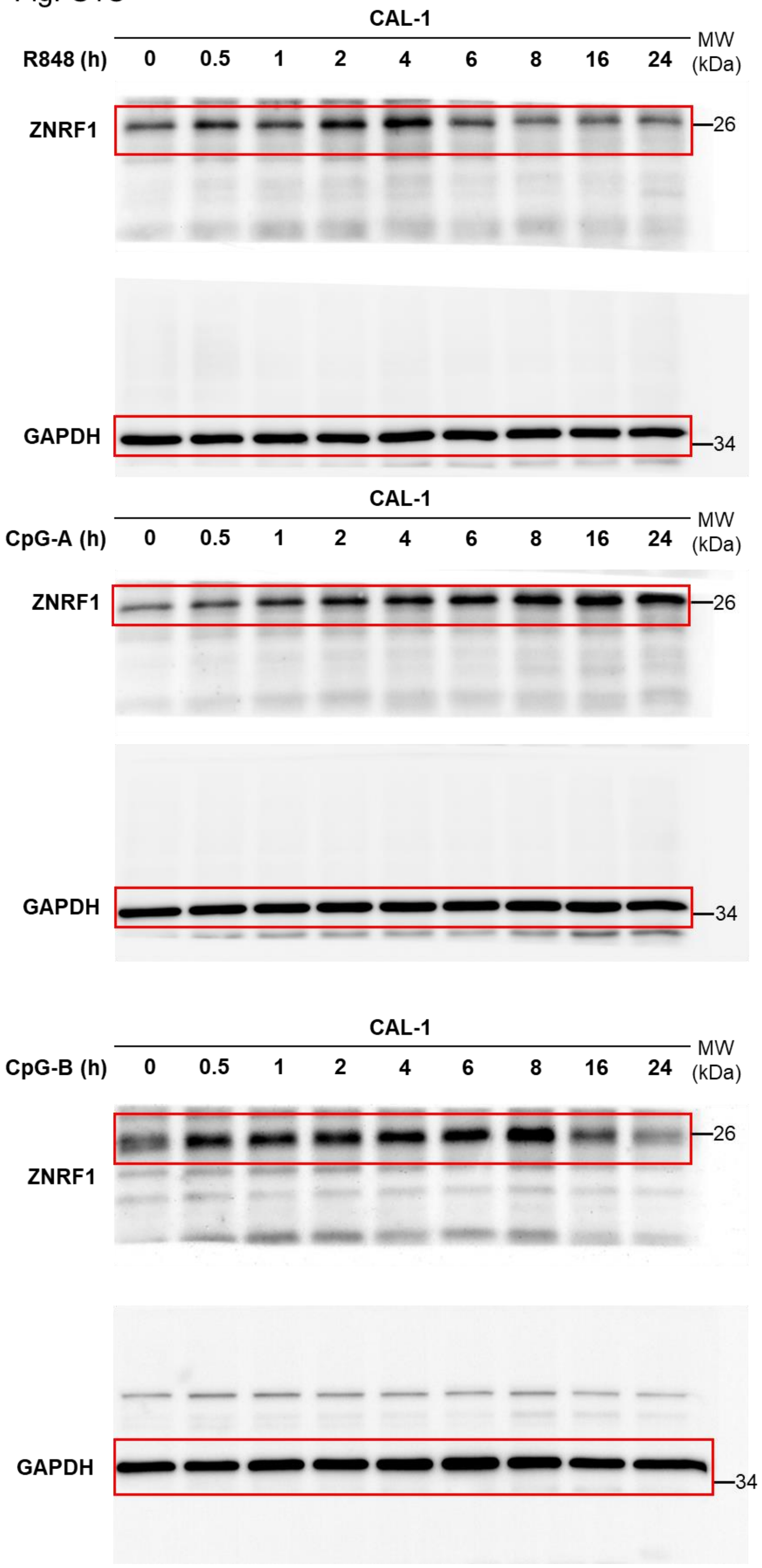

Source Data Supplementary Figure 1E

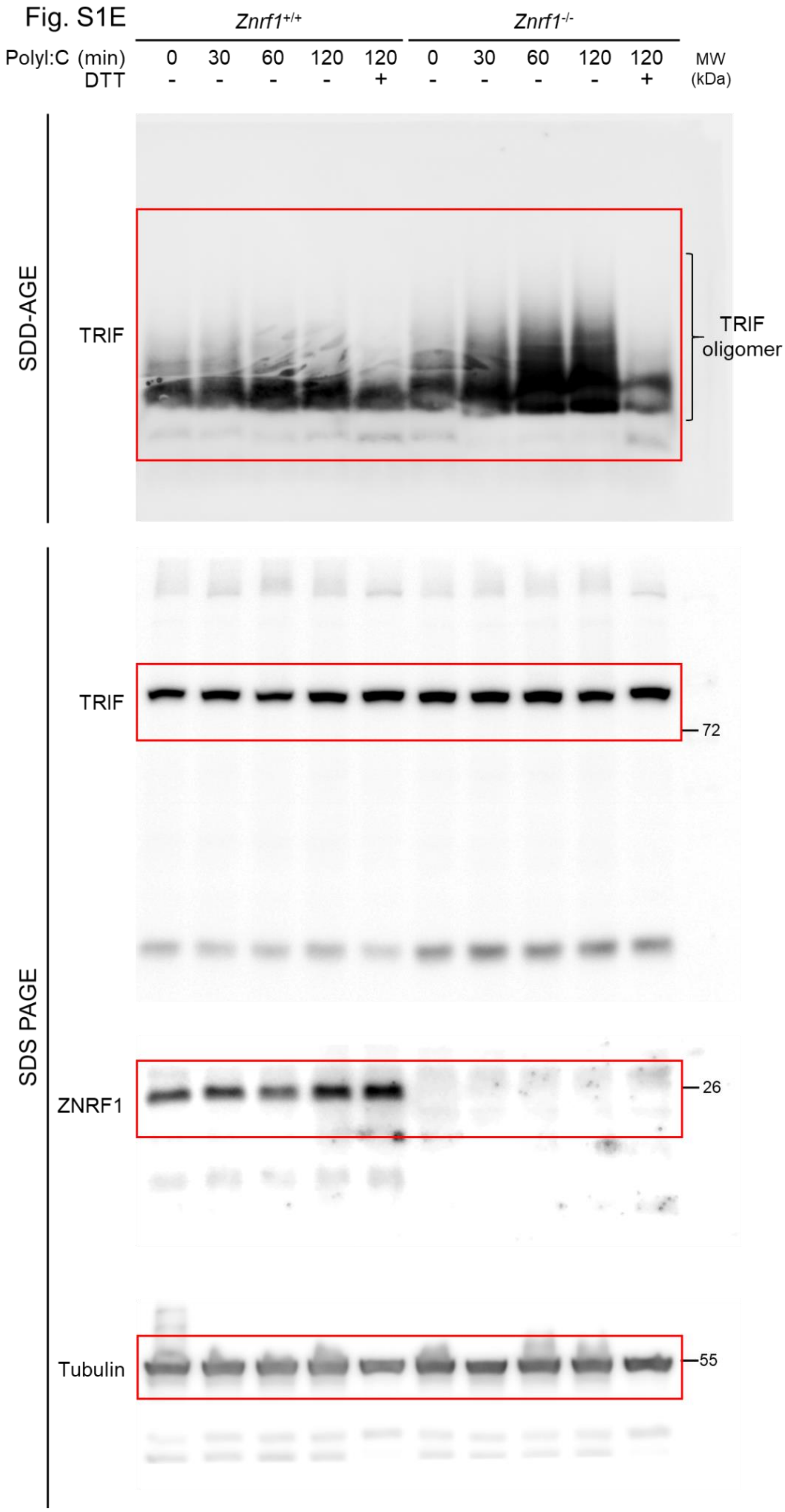

Source Data Supplementary Figure 1F

Fig. S1F

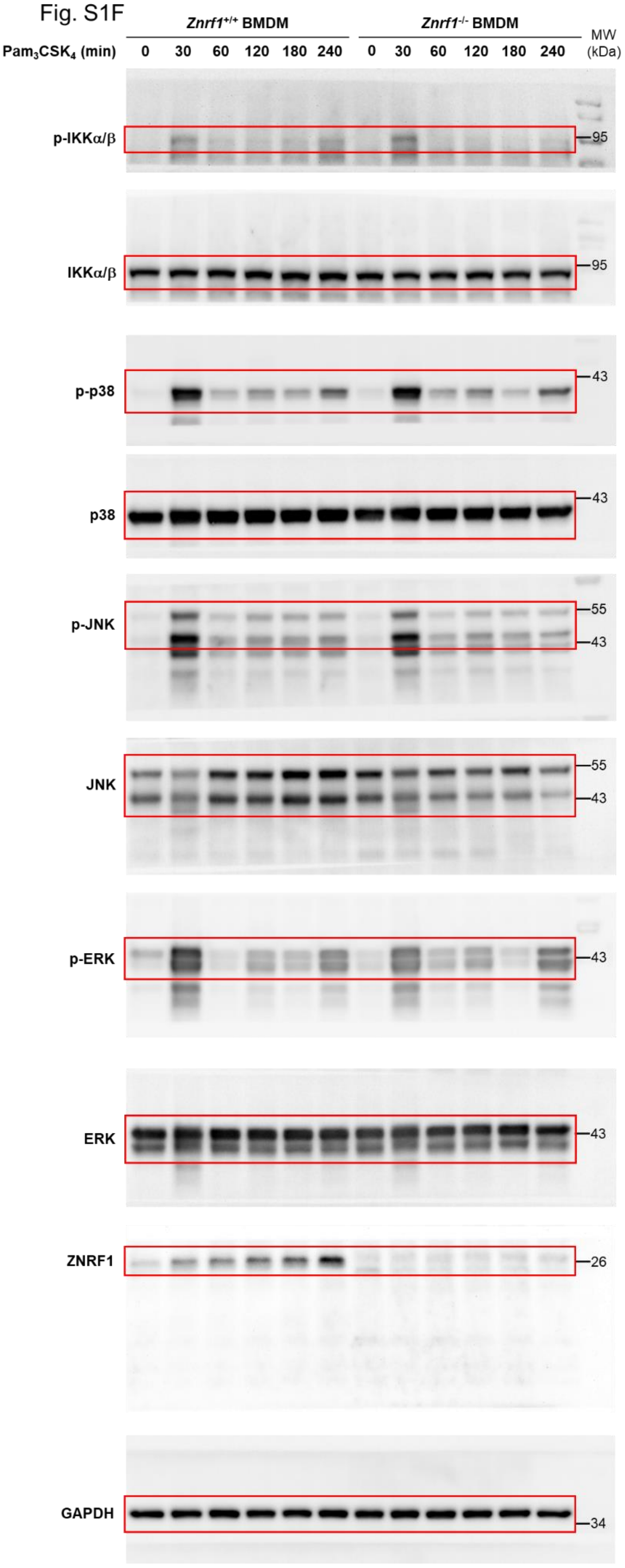

Supplement: SourceData FS1 — is the source file for Fig. S1. [file JEM_20220727_SourceDataFS1.pdf]
