# Supplementary material for: The Src–ZNRF1 axis controls TLR3 trafficking and interferon responses to limit lung barrier damage
Source: J Exp Med. 2023 May 9;220(8):e20220727. doi: 10.1084/jem.20220727 (PMC10174191; doi:10.1084/jem.20220727)

Source Data Supplementary Figure 2A

Fig. S2A

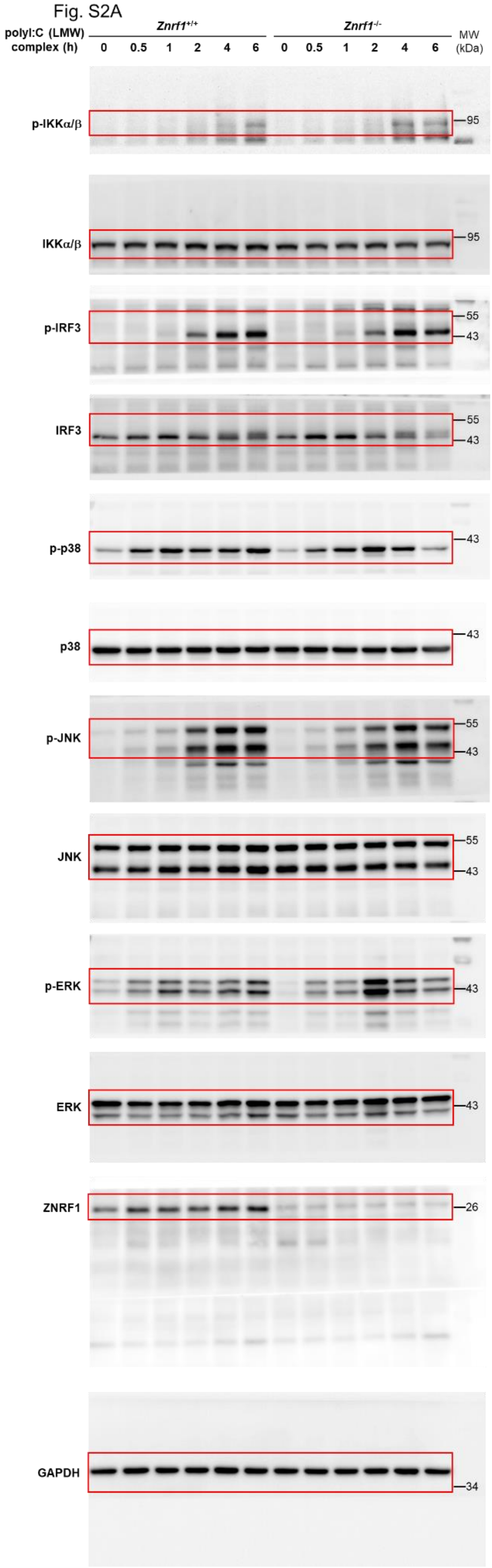

Source Data Supplementary Figure 2B

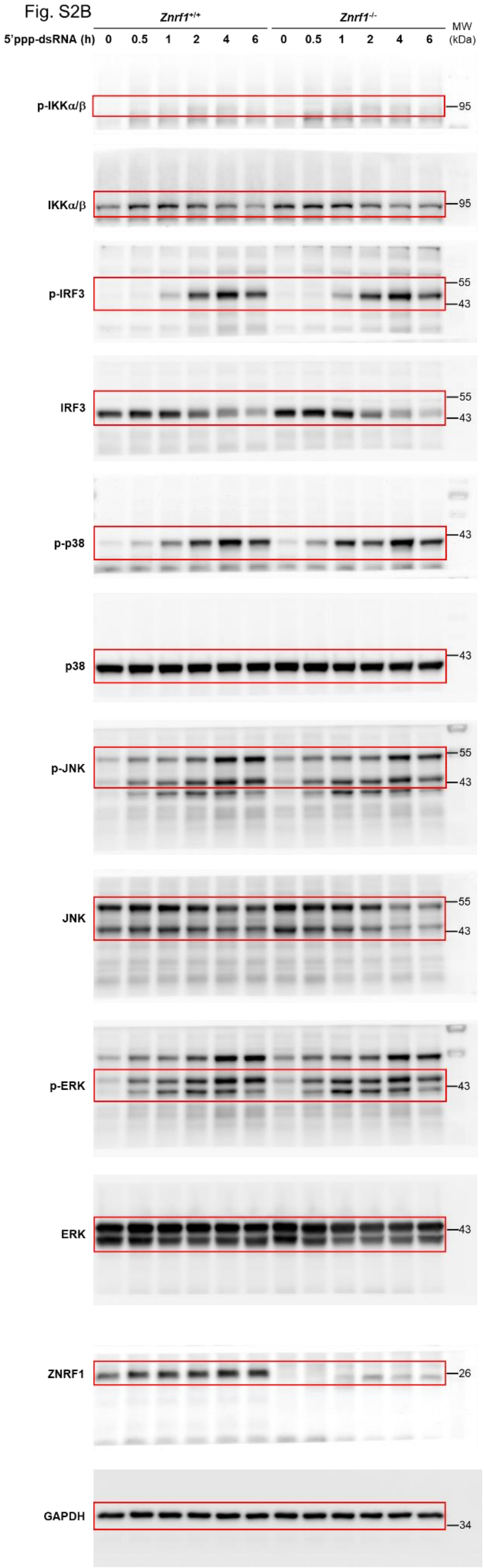

Source Data Supplementary Figure 2C

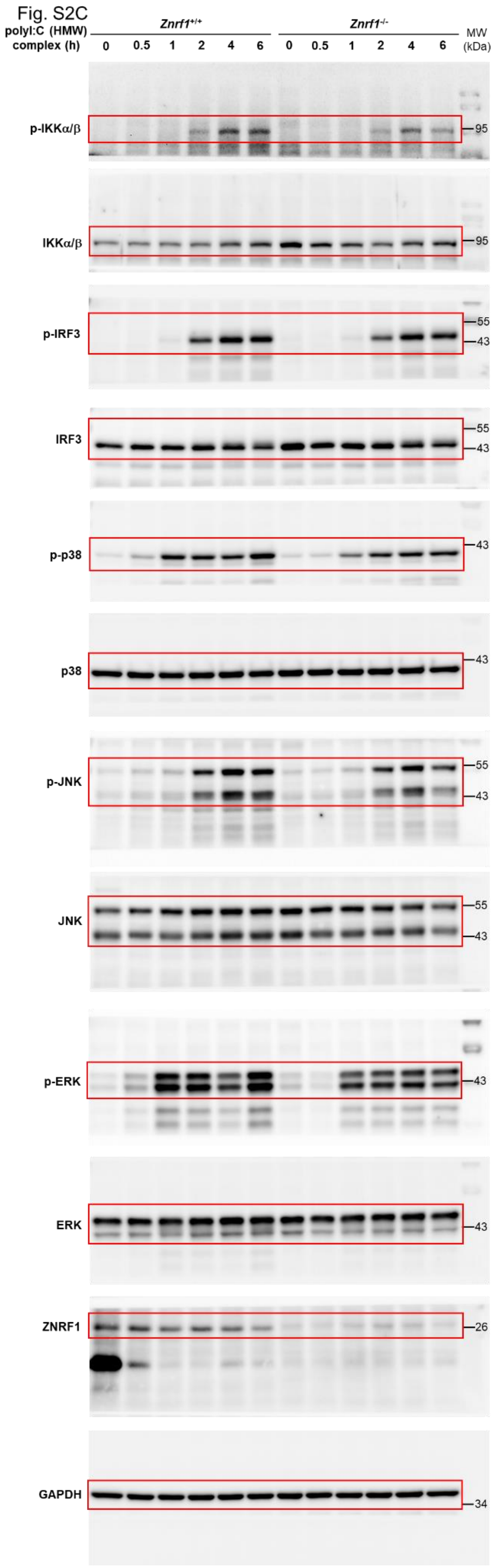

Source Data Supplementary Figure 2D

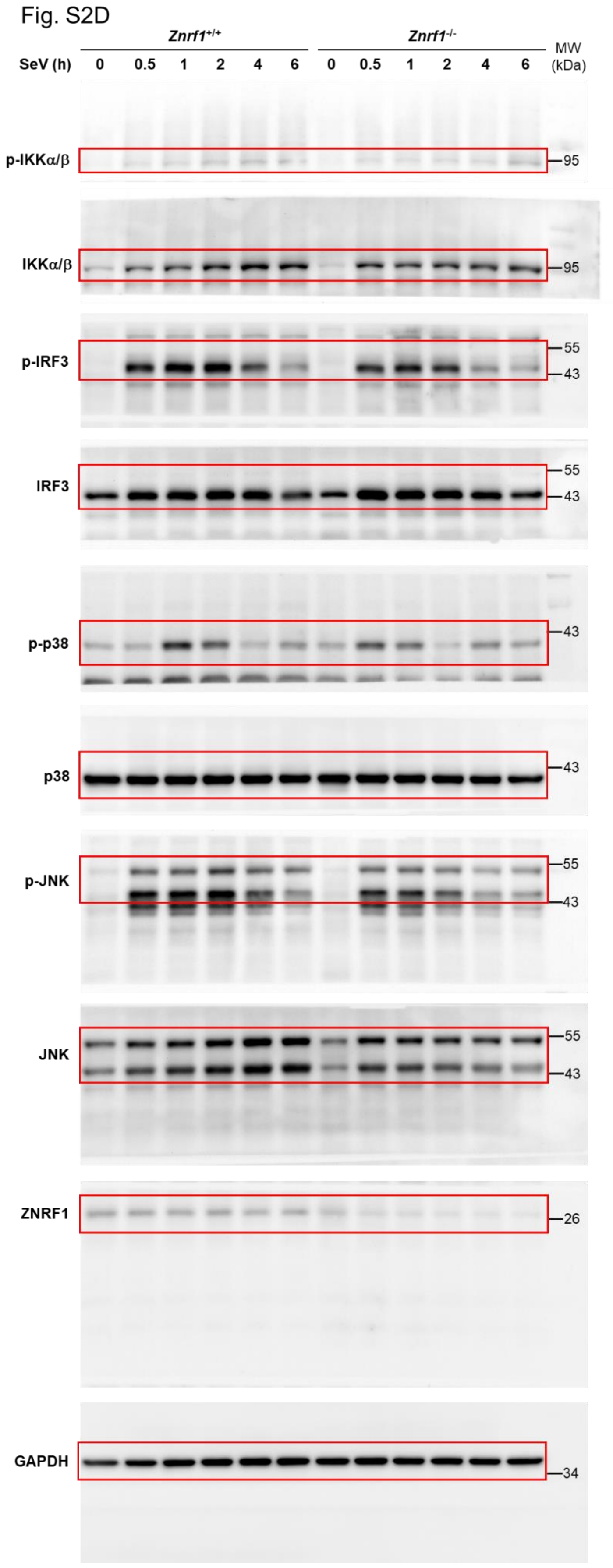

Source Data Supplementary Figure 2I

Fig. S2I

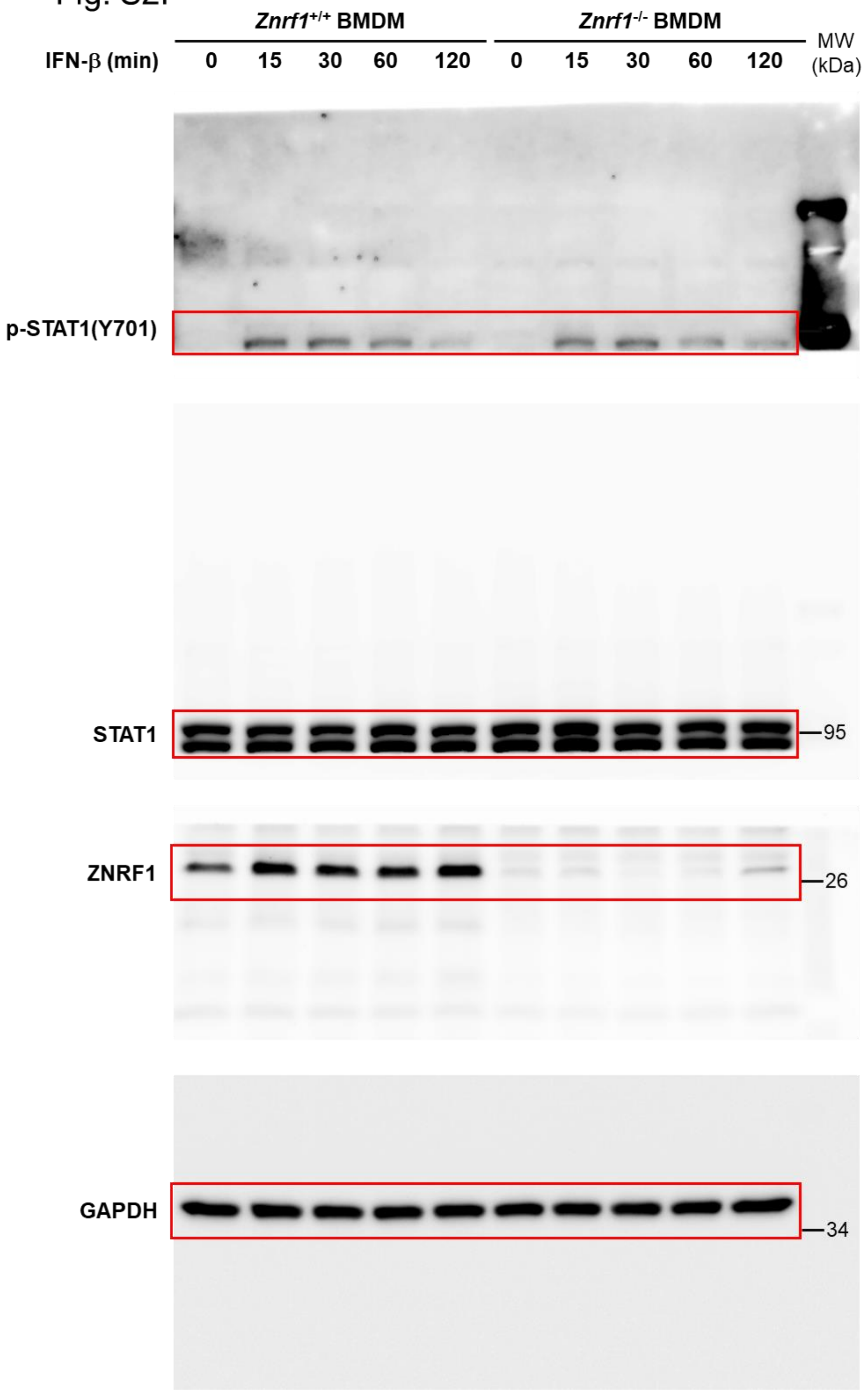

Supplement: SourceData FS2 — is the source file for Fig. S2. [file JEM_20220727_SourceDataFS2.pdf]
