# Supplementary material for: The Src–ZNRF1 axis controls TLR3 trafficking and interferon responses to limit lung barrier damage
Source: J Exp Med. 2023 May 9;220(8):e20220727. doi: 10.1084/jem.20220727 (PMC10174191; doi:10.1084/jem.20220727)

Source Data Supplementary Figure 3B

Fig. S3B

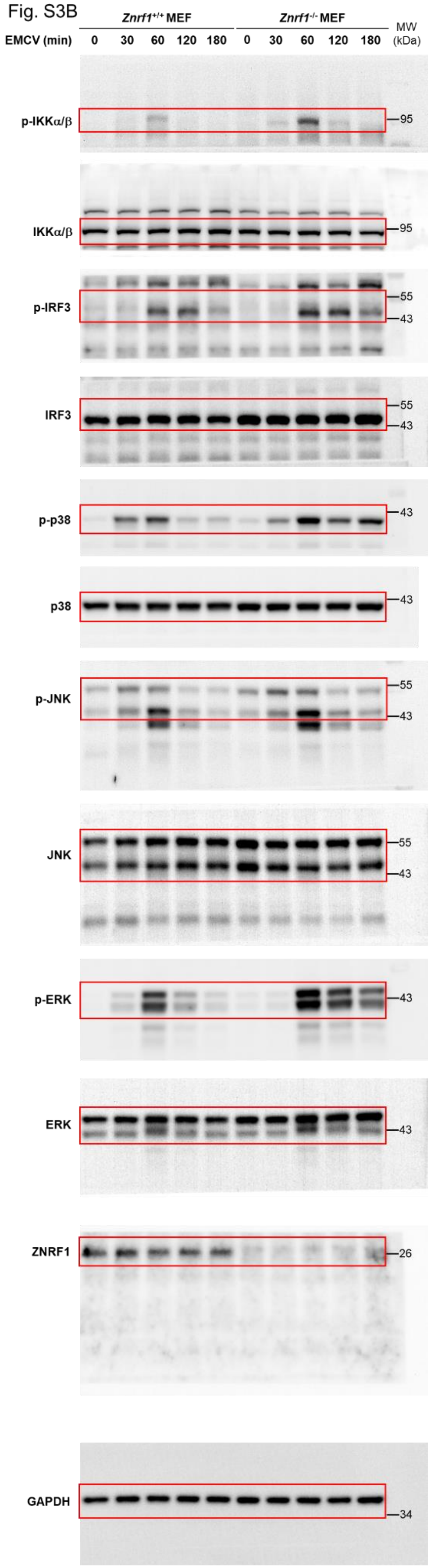

Source Data Supplementary Figure 3H

Fig. S3H

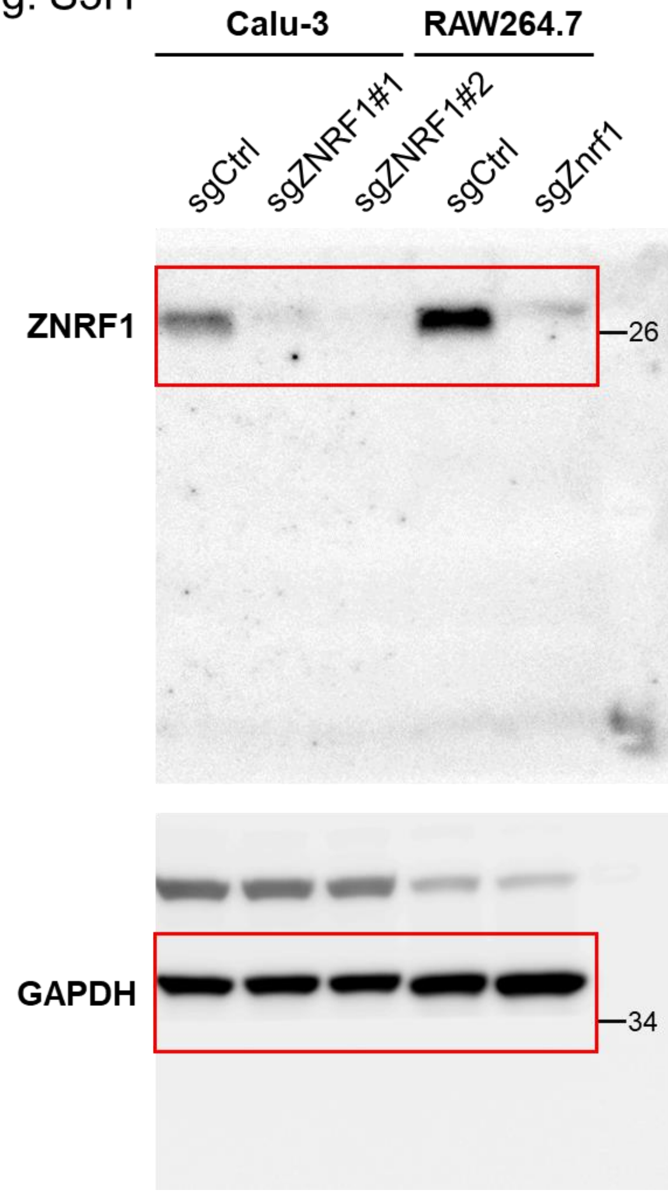

Source Data Supplementary Figure 3K

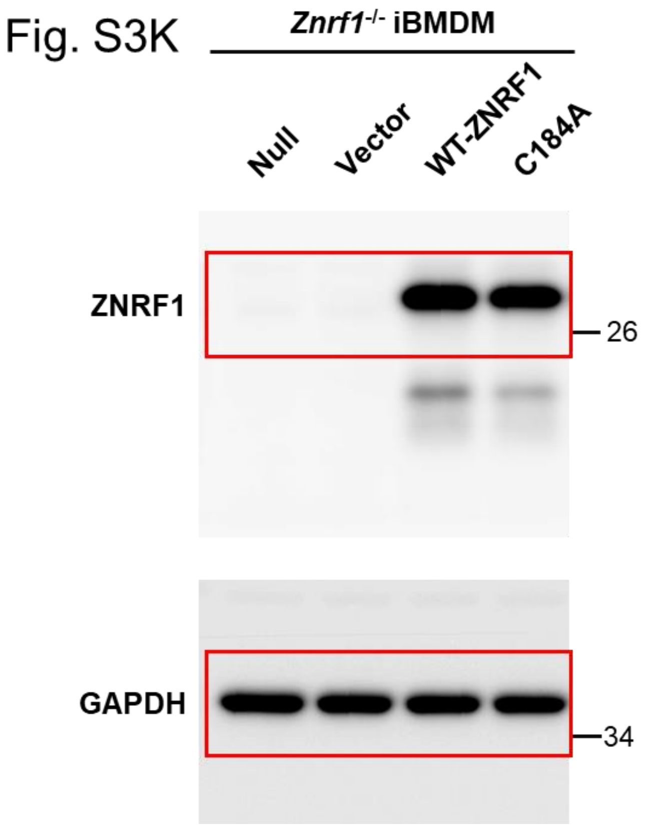

Source Data Supplementary Figure 3M

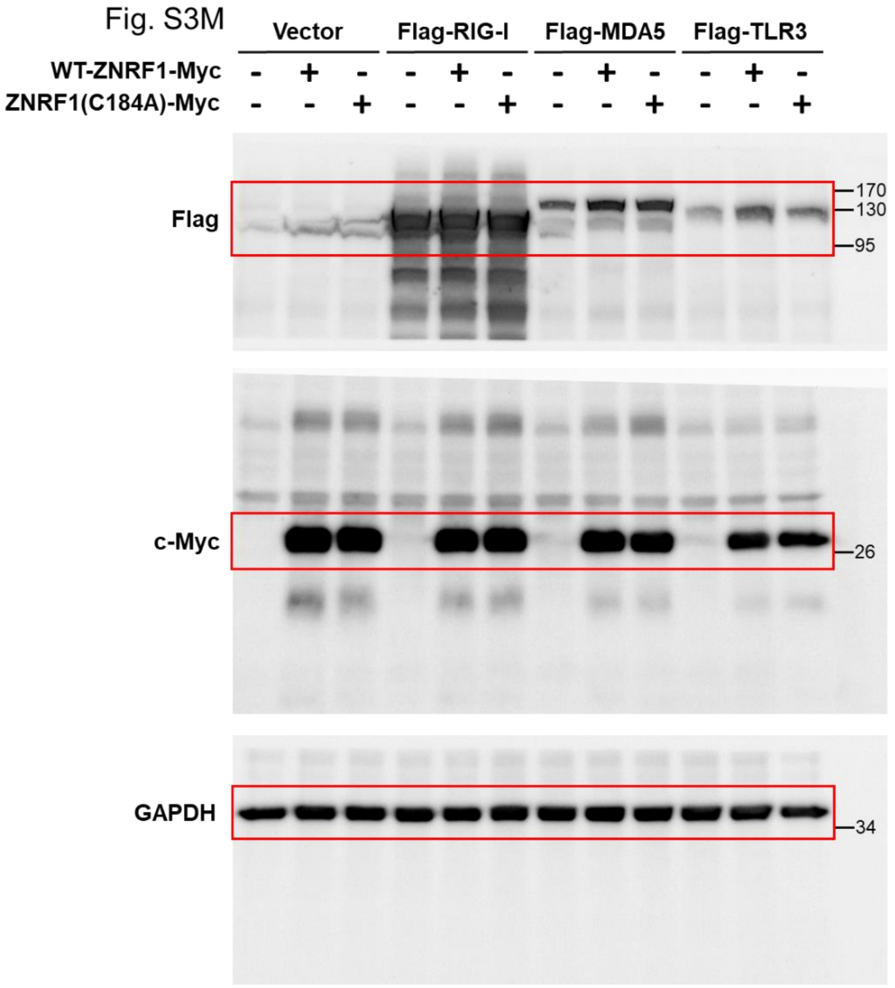

Source Data Supplementary Figure 3N

Fig. S3N

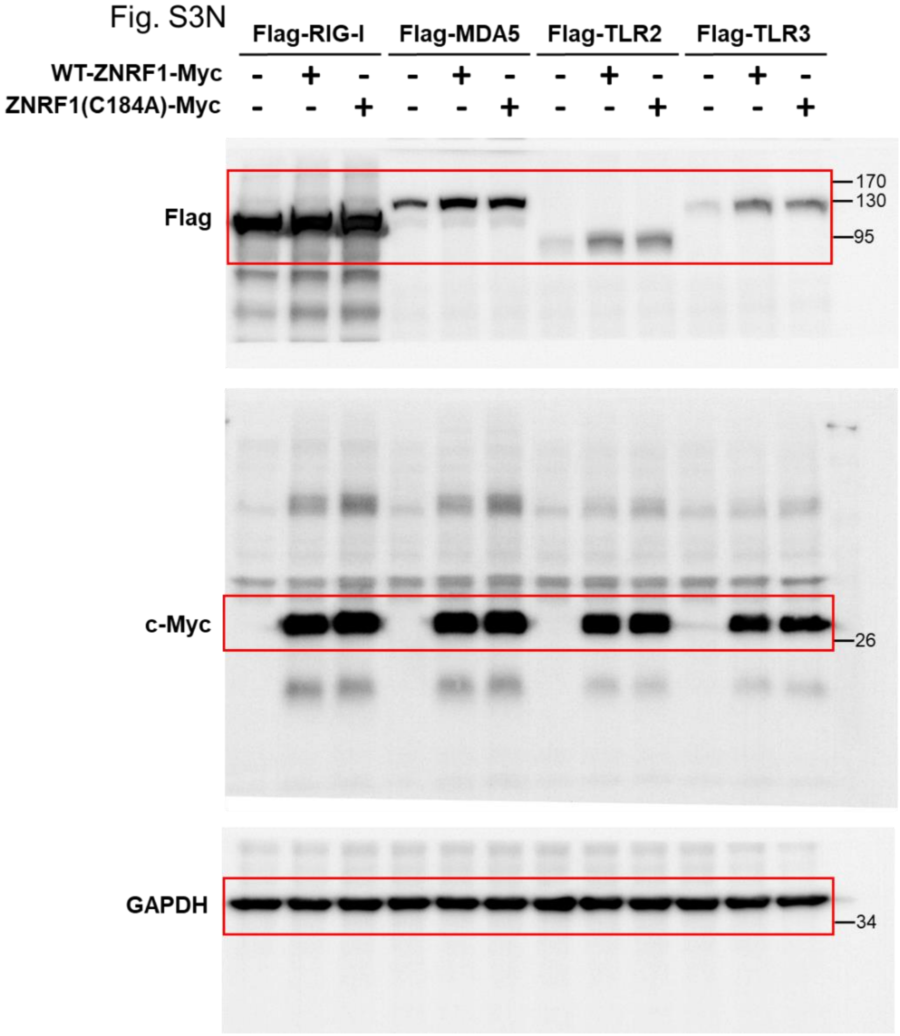

Supplement: SourceData FS3 — is the source file for Fig. S3. [file JEM_20220727_SourceDataFS3.pdf]
