# Supplementary material for: The Src–ZNRF1 axis controls TLR3 trafficking and interferon responses to limit lung barrier damage
Source: J Exp Med. 2023 May 9;220(8):e20220727. doi: 10.1084/jem.20220727 (PMC10174191; doi:10.1084/jem.20220727)

Source Data Supplementary Figure 4F

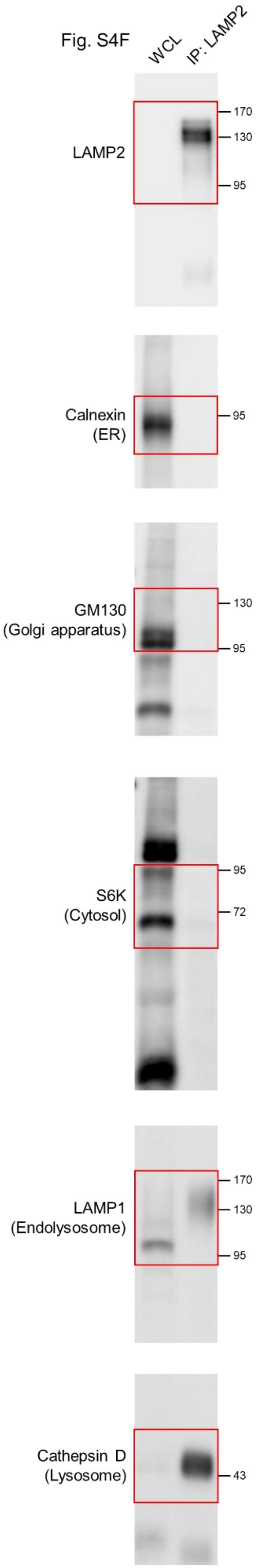

Source Data Supplementary Figure 4G

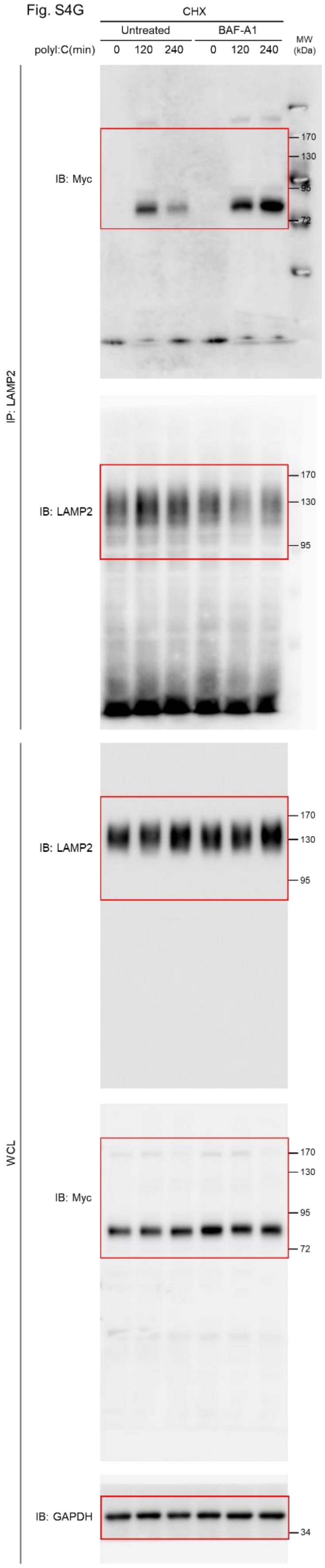

Source Data Supplementary Figure 4I

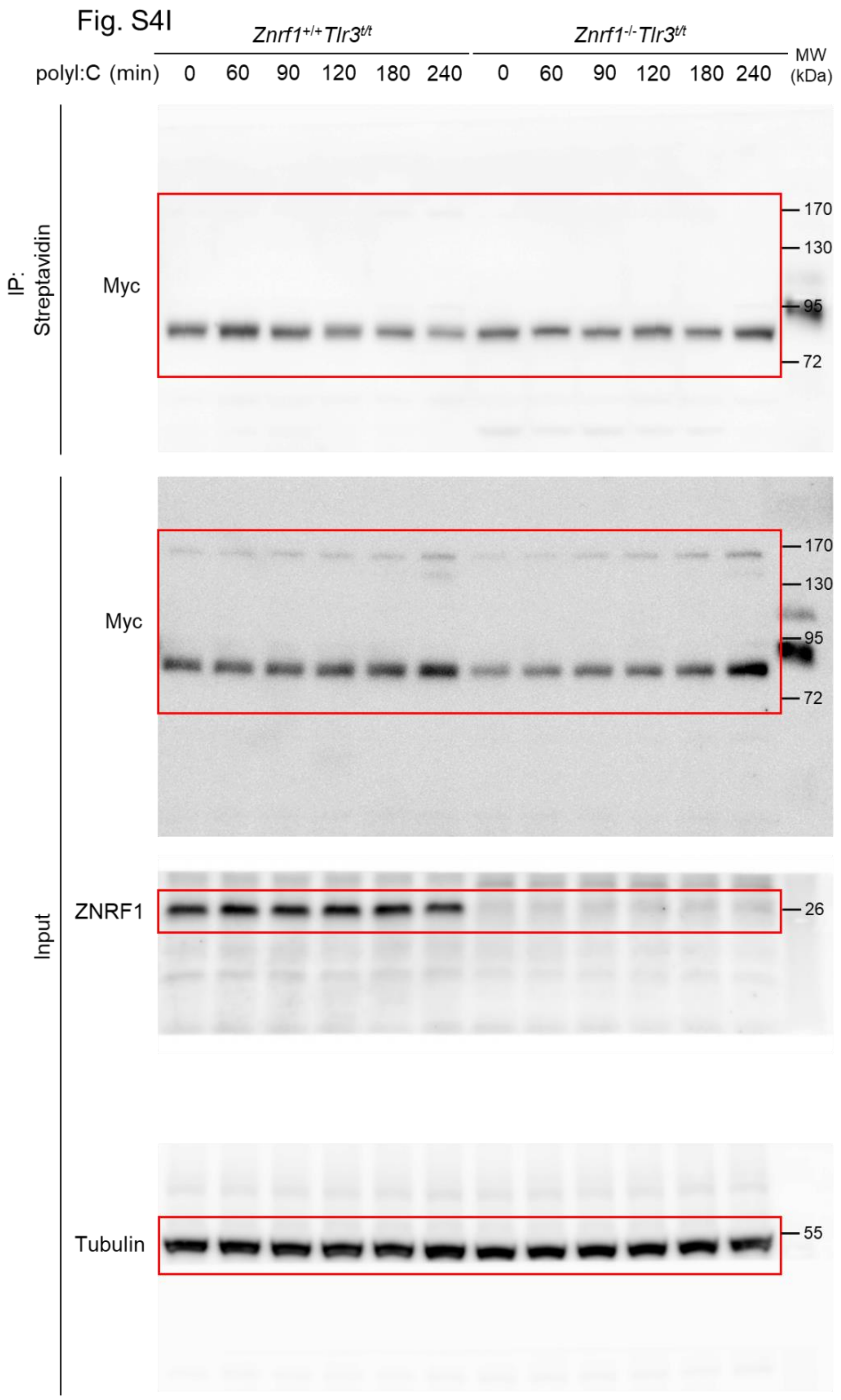

Source Data Supplementary Figure 4J

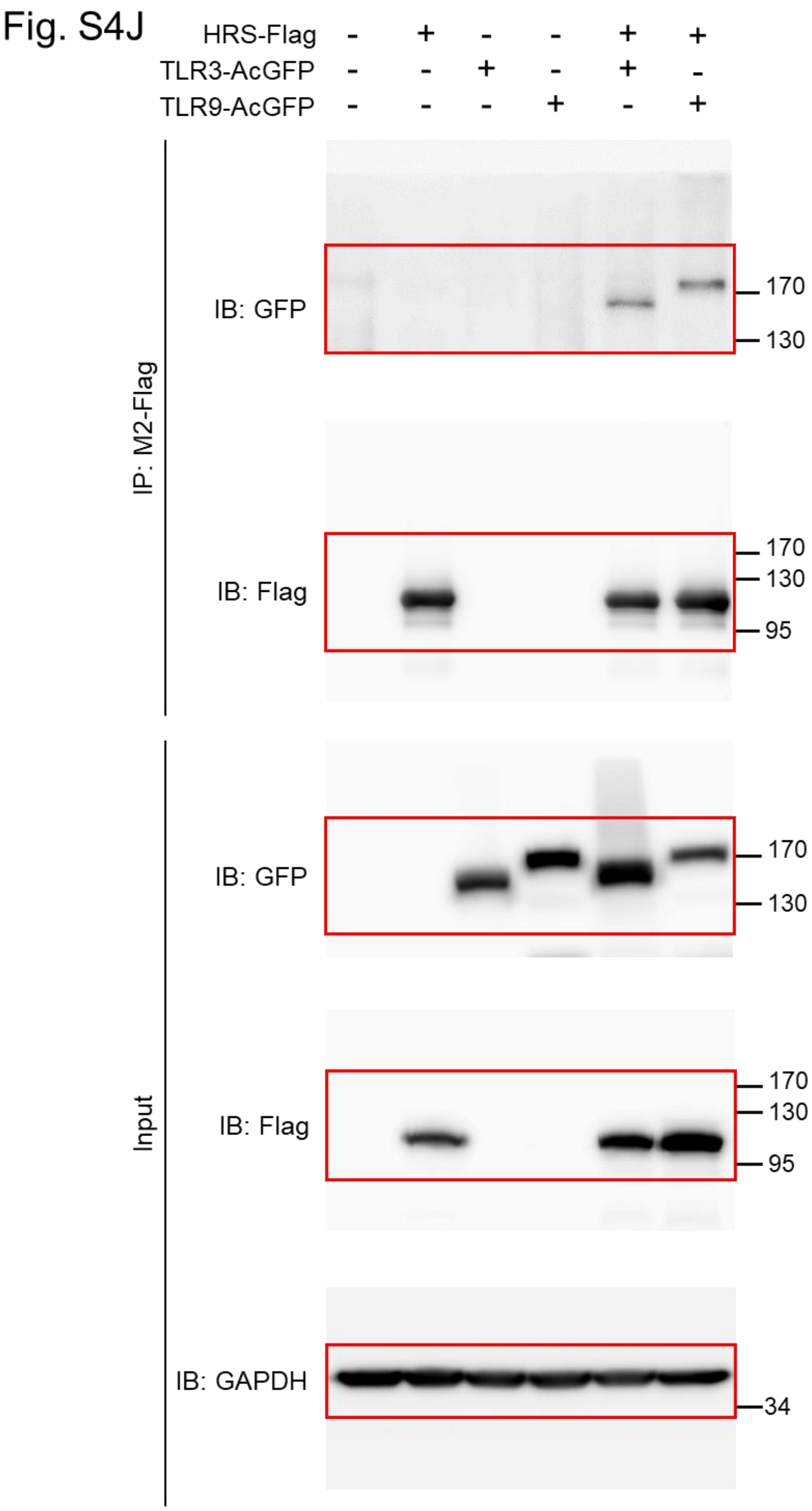

Source Data Supplementary Figure 4K

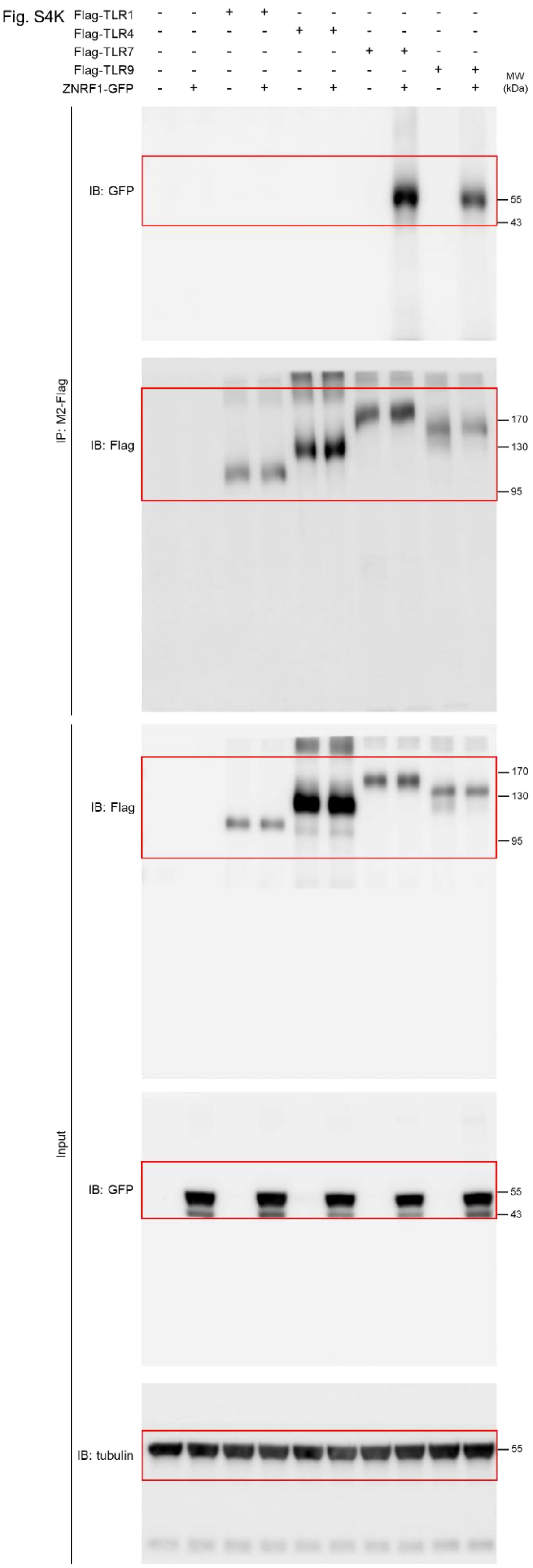

Source Data Supplementary Figure 4L

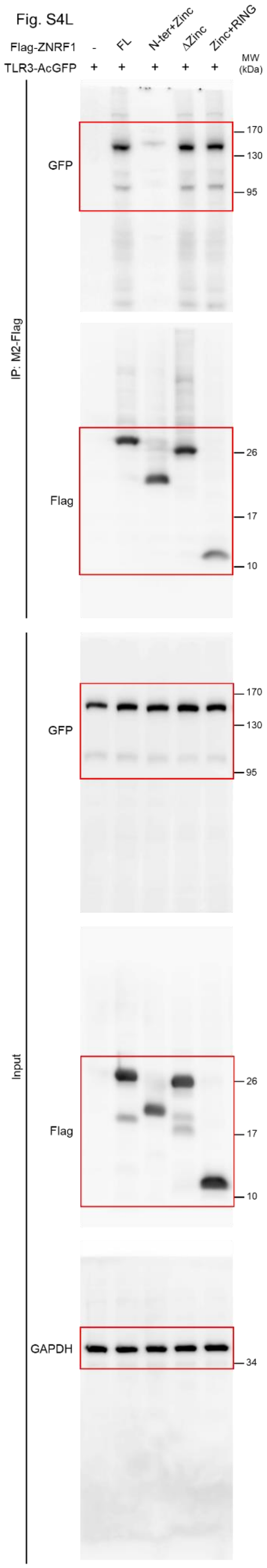

Source Data Supplementary Figure 4M

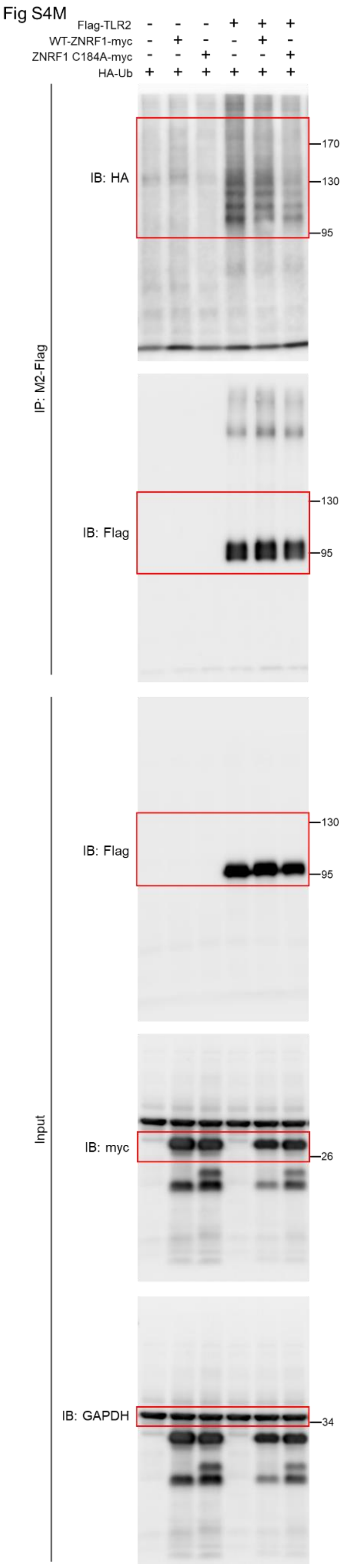

Source Data Supplementary Figure 4N

Fig. S4N

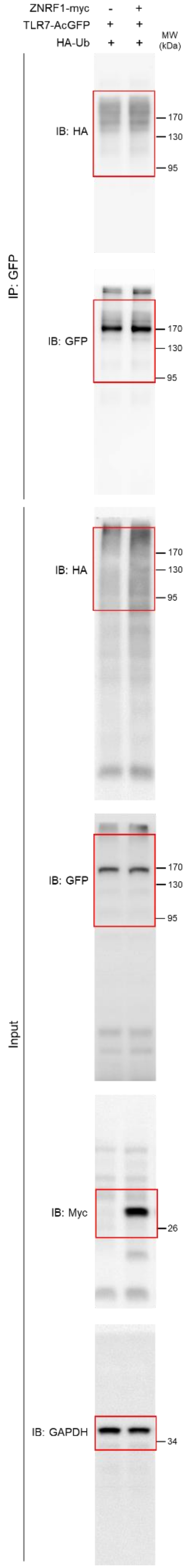

Source Data Supplementary Figure 4O

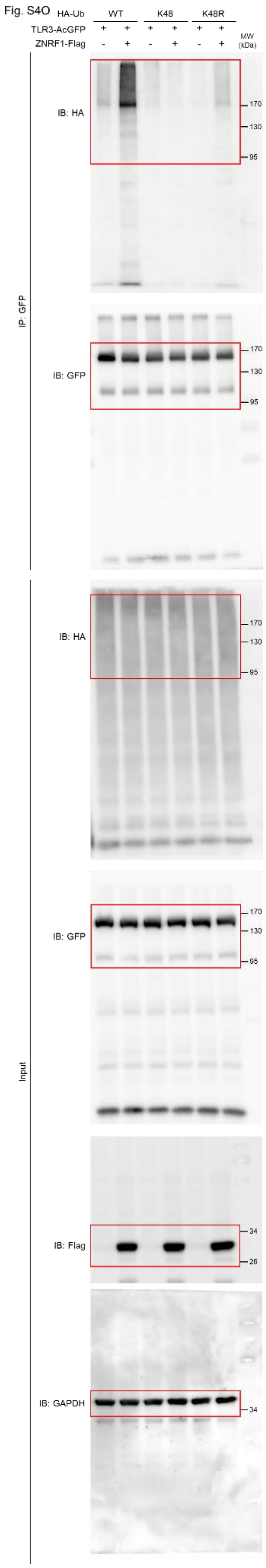

Supplement: SourceData FS4 — is the source file for Fig. S4. [file JEM_20220727_SourceDataFS4.pdf]
