# Supplementary material for: The Src–ZNRF1 axis controls TLR3 trafficking and interferon responses to limit lung barrier damage
Source: J Exp Med. 2023 May 9;220(8):e20220727. doi: 10.1084/jem.20220727 (PMC10174191; doi:10.1084/jem.20220727)

Source Data Supplementary Figure 5B

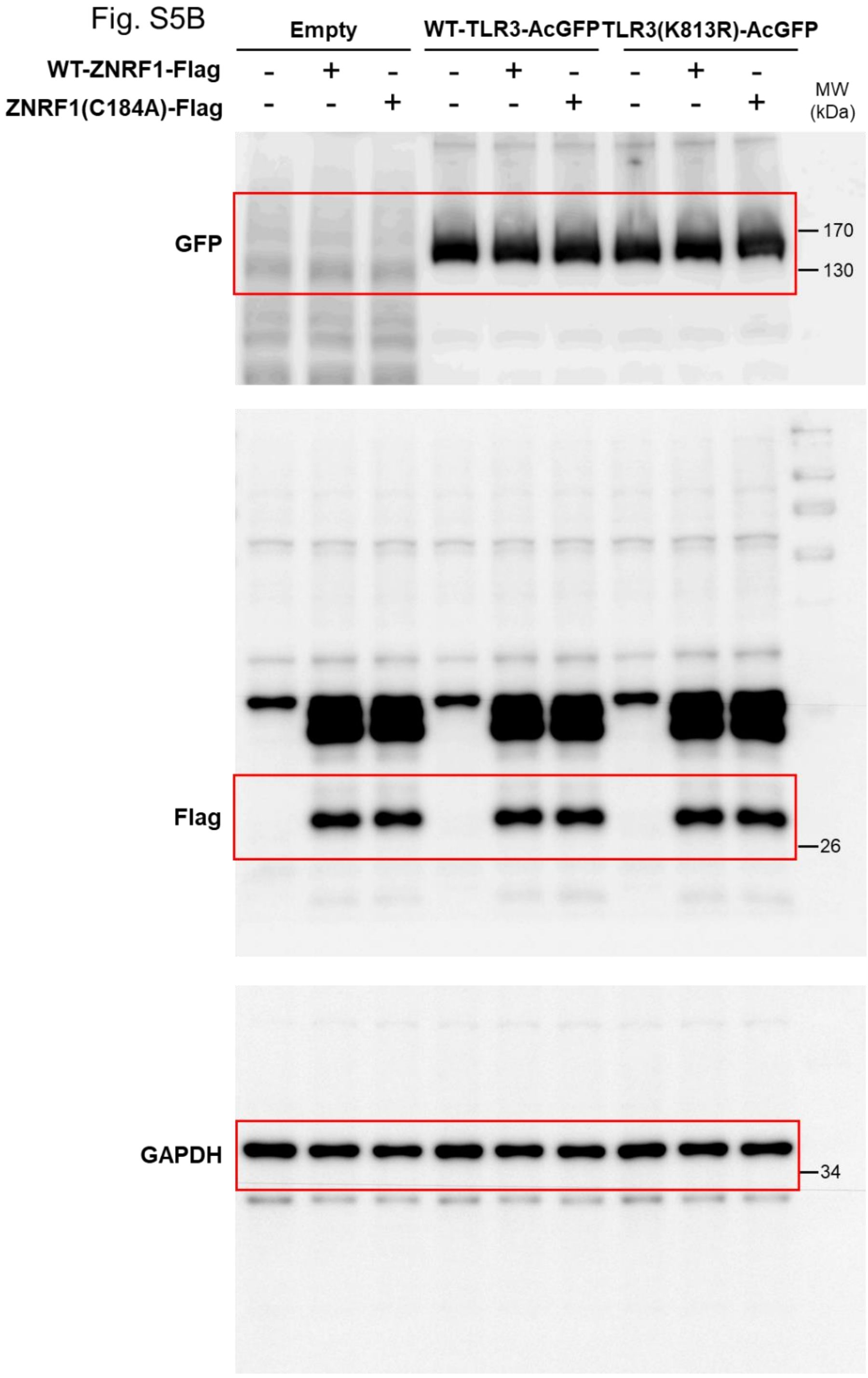

Source Data Supplementary Figure 5C

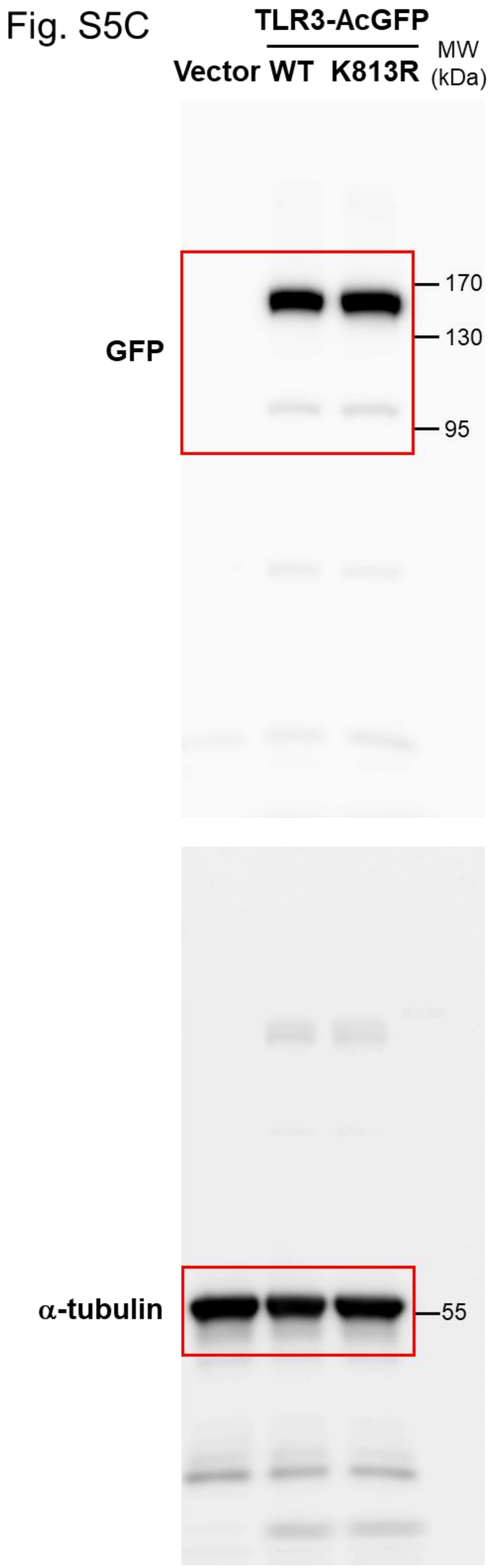

# Source Data Supplementary Figure 5D

Fig. S5D

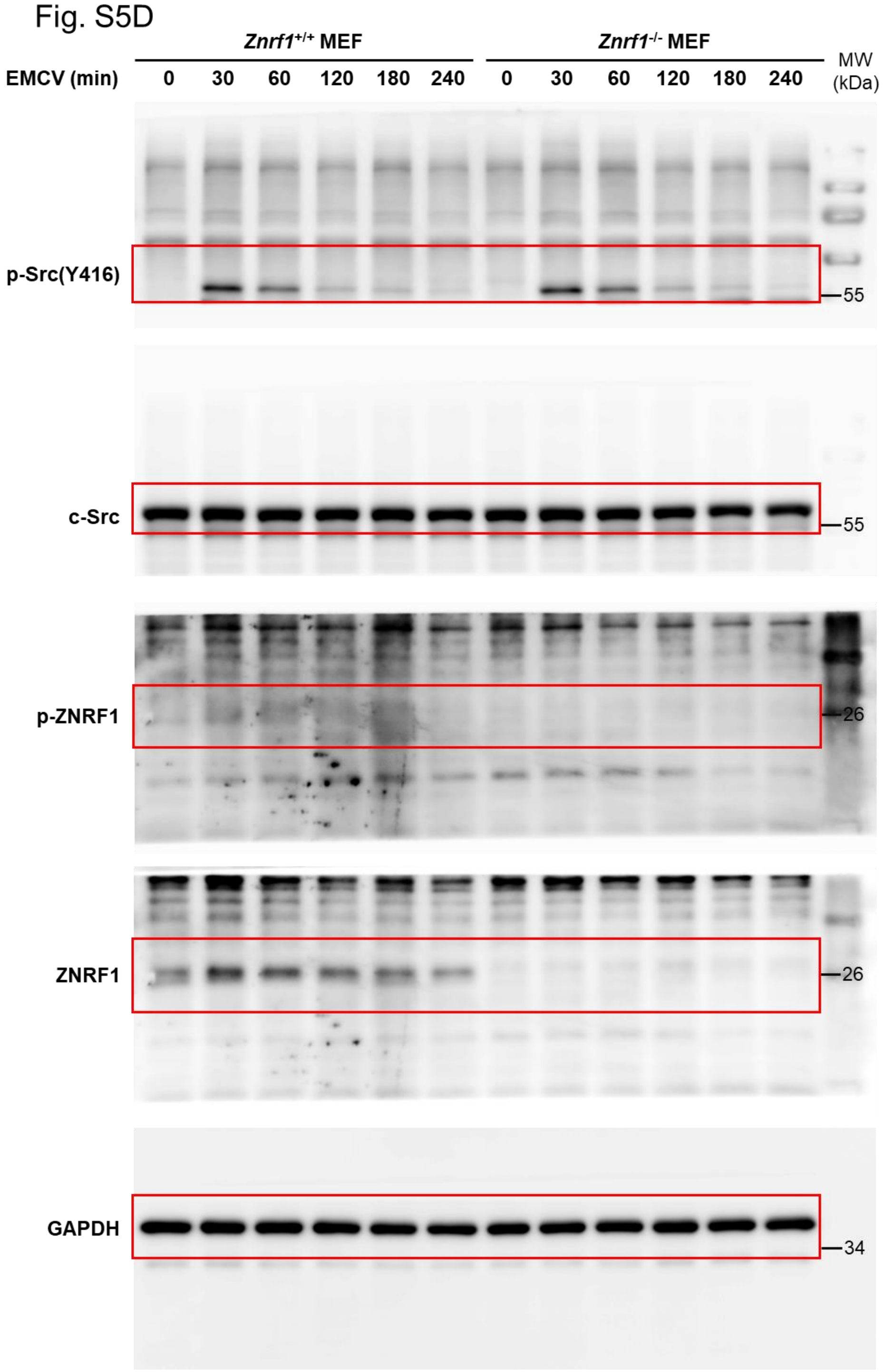

Source Data Supplementary Figure 5E

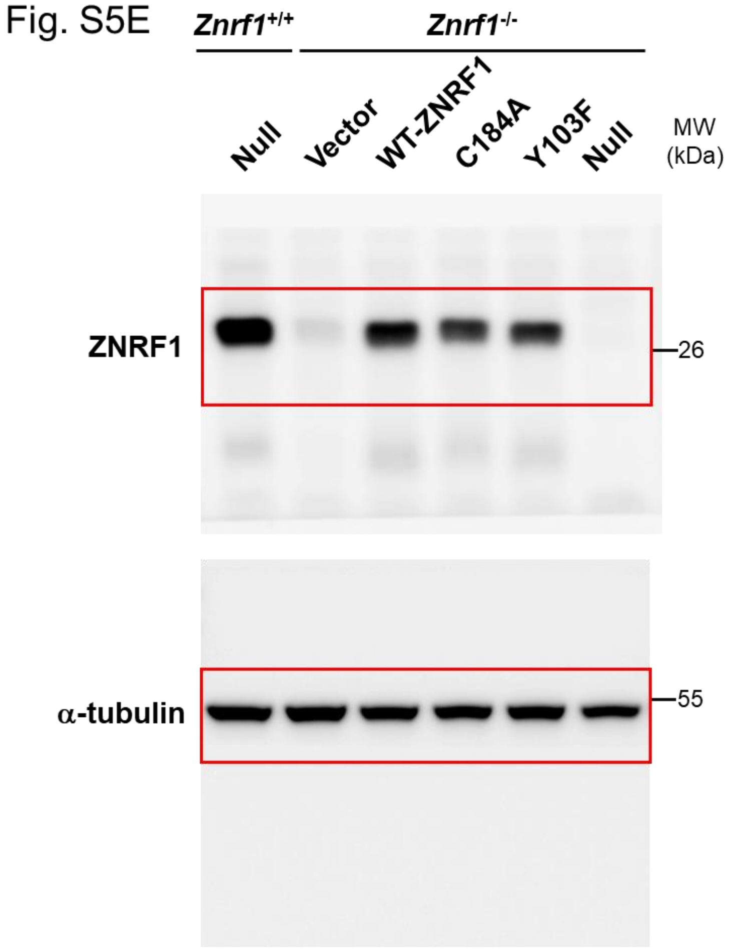

# Source Data Supplementary Figure 5I

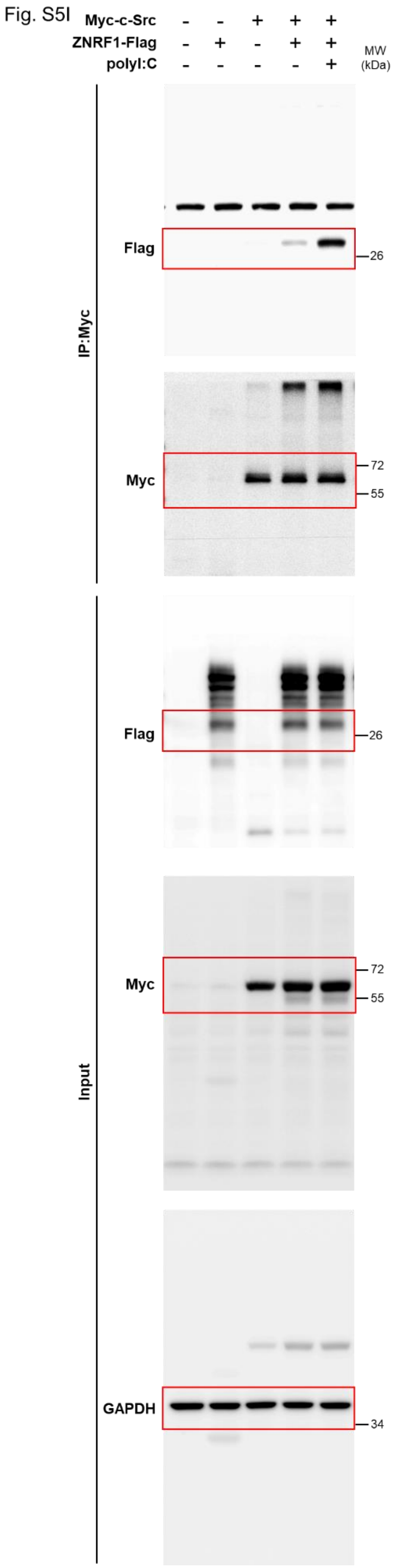

# Source Data Supplementary Figure 5J

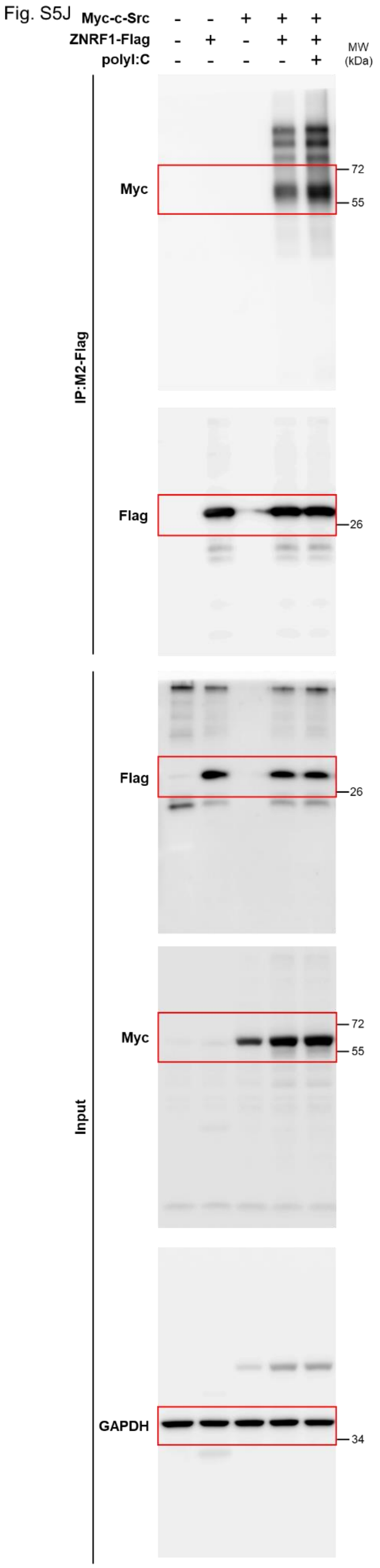

Supplement: SourceData FS5 — is the source file for Fig. S5. [file JEM_20220727_SourceDataFS5.pdf]
